# Sequencing of small RNAs of the fern *Pleopeltis minima* (Polypodiaceae) offers insight into the evolution of the microrna repertoire in land plants

**DOI:** 10.1371/journal.pone.0177573

**Published:** 2017-05-11

**Authors:** Florencia Berruezo, Flávio S. J. de Souza, Pablo I. Picca, Sergio I. Nemirovsky, Leandro Martínez Tosar, Mercedes Rivero, Alejandro N. Mentaberry, Alicia M. Zelada

**Affiliations:** 1 Laboratorio de Agrobiotecnología, Departamento de Fisiología, Biología Molecular y Celular, Facultad de Ciencias Exactas y Naturales, Universidad de Buenos Aires, Buenos Aires, Argentina; 2 Instituto de Investigaciones en Ingeniería Genética y Biología Molecular "Dr Héctor N. Torres" (INGEBI-CONICET), Buenos Aires, Argentina; 3 Departamento de Fisiología, Biología Molecular y Celular, Facultad de Ciencias Exactas y Naturales, Universidad de Buenos Aires, Buenos Aires, Argentina; 4 Departamento de Biodiversidad y Biología Experimental, Facultad de Ciencias Exactas y Naturales, Universidad de Buenos Aires, Buenos Aires, Argentina; 5 Instituto de Química Biológica de la Facultad de Ciencias Exactas y Naturales (IQUIBICEN, CONICET-UBA), Universidad de Buenos Aires, Buenos Aires, Argentina; 6 Instituto de Biodiversidad y Biología Experimental y Aplicada, Consejo Nacional de Investigaciones Científicas y Técnicas-Universidad de Buenos Aires (IBBEA, CONICET-UBA), Buenos Aires, Argentina; 7 Instituto de Agrobiotecnología de Rosario (INDEAR), Rosario, Santa Fe, Argentina; Dokuz Eylul Universitesi, TURKEY

## Abstract

MicroRNAs (miRNAs) are short, single stranded RNA molecules that regulate the stability and translation of messenger RNAs in diverse eukaryotic groups. Several miRNA genes are of ancient origin and have been maintained in the genomes of animal and plant taxa for hundreds of millions of years, playing key roles in development and physiology. In the last decade, genome and small RNA (sRNA) sequencing of several plant species have helped unveil the evolutionary history of land plants. Among these, the fern group (monilophytes) occupies a key phylogenetic position, as it represents the closest extant cousin taxon of seed plants, i.e. gymno- and angiosperms. However, in spite of their evolutionary, economic and ecological importance, no fern genome has been sequenced yet and few genomic resources are available for this group. Here, we sequenced the small RNA fraction of an epiphytic South American fern, *Pleopeltis minima* (Polypodiaceae), and compared it to plant miRNA databases, allowing for the identification of miRNA families that are shared by all land plants, shared by all vascular plants (tracheophytes) or shared by euphyllophytes (ferns and seed plants) only. Using the recently described transcriptome of another fern, *Lygodium japonicum*, we also estimated the degree of conservation of fern miRNA targets in relation to other plant groups. Our results pinpoint the origin of several miRNA families in the land plant evolutionary tree with more precision and are a resource for future genomic and functional studies of fern miRNAs.

## Introduction

Land plants (embryophytes) evolved from fresh water, streptophyte green algae during the Ordovician period, over 470 million years ago (MYA) [1, 2]. Major diversification events between the Silurian and Permian periods (385–470 MYA) gave rise to groups represented today by bryophytes *sensu lato* (liverworts, hornworts and mosses), lycophytes (clubmosses and spikemosses) and the euphyllophytes, comprising monilophytes (ferns) and spermatophytes (seed plants) [1]. Extant seed plants are divided into gymnosperms (cycads, conifers) and angiosperms. Early diversification in the Palaeozoic resulted in different strategies of alternating (gametophytic and sporophytic) generations and the origination of key anatomical features in different groups of plants, including vascular systems and various types of meristems, leaves and roots, as well as seeds [1, 3]. The origin and evolution of these and other features is studied with fossils and comparative anatomical and physiological studies, but comparative genomics of extant species can also help understand plant evolution. In the last decade, several angiosperm genomes have been sequenced, as well as the genomes of a moss (*Physcomitrella patens*), a lycopod (the spikemoss *Selaginella moellendorffii*) and some conifer trees [4–9]. Genomic analyses show that land plants share a common set of transcriptional regulators, including transcription factors and microRNAs, that set them aside from green algae [3, 10].

MiRNA genes are transcribed as long, primary RNA precursors (pri-miRNAs) that can form a foldback, hairpin structure. In plants, pri-miRNAs are processed by Dicer-like 1 (DCL1) protein to generate a miRNA/miRNA* duplex. The miRNA* ("star") strand is generally degraded, whereas the mature miRNA molecule is incorporated into a RNA-induced silencing complex (RISC) that contains Argonaute 1 protein (AGO1). The ~21 nucleotide (nt) miRNA associated with RISC serves as a guide to recognize specific messenger RNAs (mRNAs), interfering with their translation or leading to mRNA degradation [11, 12]. MiRNAs are, thus, negative regulators of gene expression. Analyses of land plant genomes and small-RNA transcriptomics have revealed that many miRNA genes are common to all plant groups, suggesting that they originated before or during the colonization of land by plant ancestors. Many of these deeply conserved miRNAs play important roles in plant development and have likely contributed to the diversification of land plant body plans during evolution [10, 13–16].

**Fig 1:**
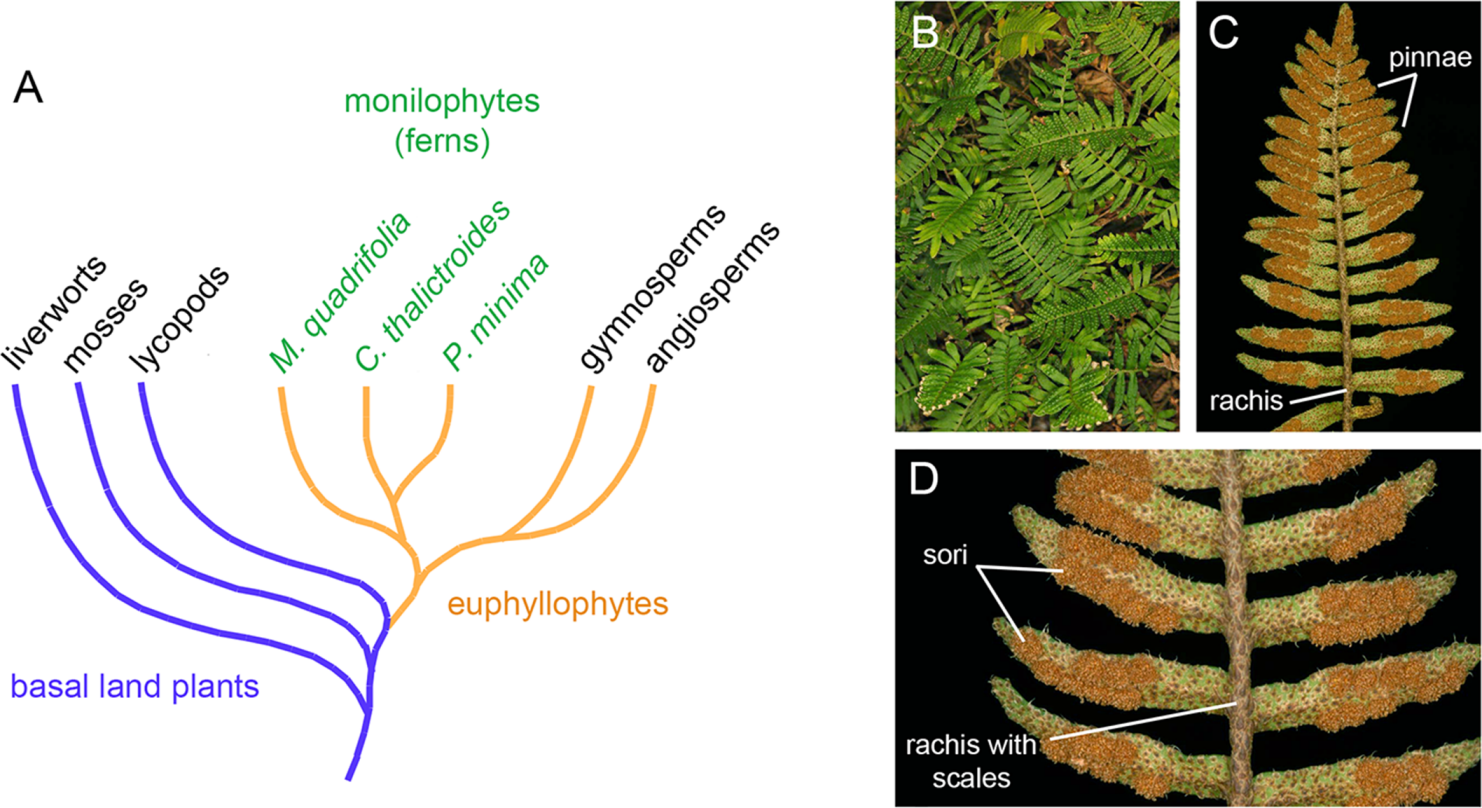
Phylogenetic position and habit of *Pleopeltis minima*. **(A)** Simplified summary of phylogenetic relationships among major plant lineages relevant for the present study [3, 17, 24]. Note that some molecular phylogenies recover a clade composed of liverworts and mosses as sister to vascular plants [25, 26]. The fern (“monilophytes”) clade is the sister group to seed plants, gymnosperms and angiosperms. Within ferns, *P*. *minima* belongs to the order Polypodiales and is more closely related to *Ceratopteris thalictroides*, while *Marsilea quadrifolia* belongs to the order Salviniales and *Lygodium japonicum* to the order Schizaeales. **(B)** Habit of *P*. *minima* growing as epiphyte upon a tree trunk. **(C)** Abaxial side of leaf surface showing form and organization. **(D)** Portion of fertile leaf showing position of sori and scales. Scale bars: 5 mm.

Our understanding of plant miRNA evolution is hampered by biased sampling, since most genomic and transcriptomic studies have been carried out in angiosperms. In particular, there is a dearth of knowledge on the miRNA repertoire of ferns. The fern clade (Monilophyta) [17] comprises today around 12,000 species. It occupies a special place in the plant phylogenetic tree as the sister group of seed plants (Fig 1A). Thus, the study of ferns should illuminate critical aspects of plant evolution, like the transition from homospory to strict heterospory, sporophyte dominance, as well as aspects of the development of leaves, roots and vascular systems that distinguish ferns and seed plants [3, 18]. No fern nuclear genome has been sequenced to date, although some genomic surveys and transcriptomic analyses have been carried out [19–21]. An early survey based on hybridization arrays indicated the presence of eight miRNAs in the fern *Ceratopteris thalictroides* that are also conserved in other land plants [22]. More recently, a broad survey of several land plants included an aquatic fern, *Marsilea quadrifolia*, which added some conserved miRNAs to the fern repertoire [23].

Here, we study a South American fern, *Pleopeltis minima* (Bory) J. Prado & R.Y. Hirai (synonyms: *Polypodium squalidum* Vell.; *Pleopeltis squalida* (Vell.) de la Sota) [27]. *P*. *minima* is a small, mostly epiphytic fern belonging to the Polypodiaceae family that occurs in forests in Argentina, Bolivia, Southern Brazil, Paraguay and Uruguay. Its scaly rhizome bears several 2–10 cm long compound leaves (fronds), divided into pinnae (leaflets). Fertile leaves possess exindusiate sori (cluster of sporangia) of round shape on the abaxial side (Fig 1B–1D). One particular characteristic of *P*. *minima* is its dissecation tolerance (poikilohydry) [28, 29]. We have sequenced the small RNA fraction of *P*. *minima* and searched for miRNAs phylogenetically conserved in other plants. We present a group of conserved fern miRNAs and their predicted targets, thereby advancing our understanding of miRNA evolution in the fern and land plant lineage.

## Results

### Sequencing and analysis of *P*. *minima*s RNAs

Small RNAs (sRNAs) were extracted from two samples of fertile fronds of *P*. *minima*, converted to cDNA and subjected to Illumina sequencing, generating a total of 25,947,729 reads, of which 20,989,537 were high-quality reads with lengths between 15 and 45 nt. Of these, 6,268,973 reads corresponding to mRNA fragments, tRNAs, rRNAs, snoRNAs, snRNAs and repetitive elements were discarded, leaving a total of 14,720,564 sRNA reads to be analysed (S1 Table).

**Fig 2:**
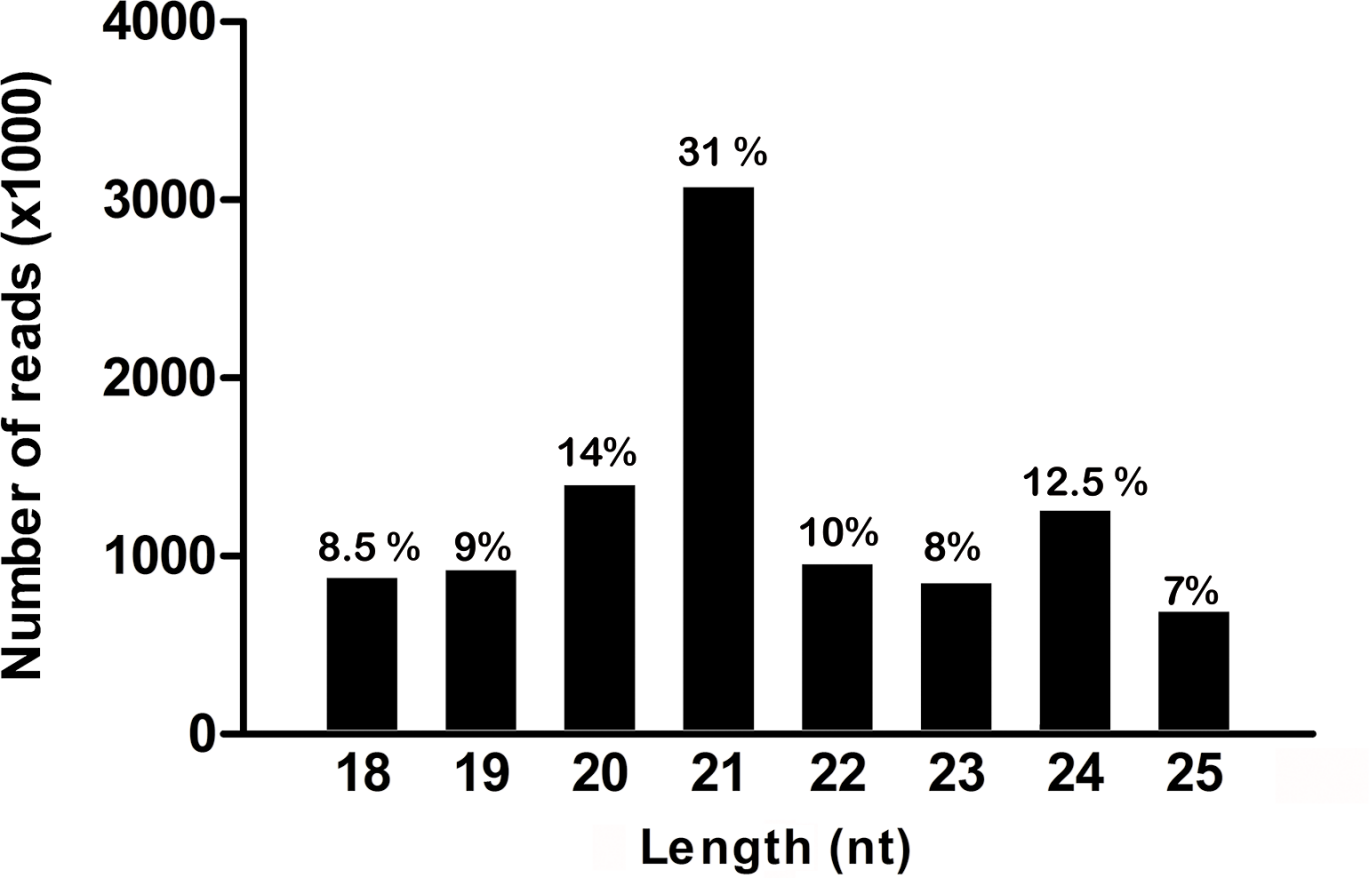
Size distribution of *P*. *minima* sRNA sequences. The graph displays the raw number of sRNA reads in the range of 18 to 25 nt. The percentage of reads in each size category in the range is indicated. A large peak at 21 nt and a smaller peak at 24 nt are noticeable.

The length of functional sRNAs are thought to lay in the 20–24 nucleotide (nt) range. In most angiosperms, sRNA length distribution typically displays two peaks at 21 and 24 nt, with miRNAs centred around 21 nt, while 24 nt small-interfering RNAs (siRNAs) are associated with silencing of retroposons by heterochromatinization [12]. In non-angiosperms, the presence of the 24-nt sRNA peak is not always evident, but it has recently been shown that the heterochromatic siRNA pathway is a primitive feature of land plants, being present in mosses [30]. In *P*. *minima*, we observed a prominent 21 nt peak and a smaller, but noticeable, peak at 24 nt (Fig 2). A similar distribution is found in the aquatic fern *M*. *quadrifolia* [23], showing that the formation of 24-nt sRNAs, probably associated with heterochromatin formation, is a common characteristic of ferns.

### Identification of conserved *P*. *minima* miRNAs

Since *P*. *minima* lacks a genome sequence and no fern genome is available for comparison, we focused on finding miRNAs in our samples that are conserved in other land plant genomes. To this end, the 14,720,564 sRNA reads of *P*. *minima* were compared to version 21 of miRBase [31], yielding 1,127,970 reads that could be aligned to miRNAs or MIRNA genes in the database, corresponding to 3,912 unique sequences (S1 Table).

It is known that the confidence on the existence of miRNA families in miRBase varies widely, and the evidence for many miRNA families is rather weak, specially for miRNAs coming from species lacking a genome sequence [11]. In view of this, to identify bona fide *P*. *minima* miRNAs we required the miRNA families 1) to be represented by at least 10 reads of 20–22 nt in our data; 2) to exhibit consistent 5' cleavage processing of the mature miRNA strand and 3) to be identical or very similar to highly confident sequences of mature miRNA or miRNA* strands of other plant species, as defined by [11]. For some miRNA families, namely miR162, miR395 and miR477, even though the total number of reads was under 10, we still considered them to be real miRNAs since the reads included both miRNA and miRNA* strands that were identical or nearly identical in sequence to the corresponding miRNA and miRNA* sequences from other plants (S2 Table).

The analysis revealed a total of 57 conserved miRNAs in *P*. *minima*, belonging to 23 miRNA families (Table 1). MiR156 is related in sequence and function to miR529 [32, 33], leading to them being often considered to be members of the same family [11], the same happening between miR159 and miR319 [34, 35]. Thus, if miR156/miR529 and miR159/miR319 are regarded as two families, the total number of conserved *P*. *minima* miRNA families would be 21 instead of 23. MiR1030 is a miRNA that has been recognised by this name in the moss, *P*. *patens* [13], as well as in the liverwort, *M*. *polymorpha* [36]. However, it is closely related in sequence to miR530 of seed plants, and we decided to consider it as an unique family, miR530/1030, as suggested by Taylor et al [11].

**Table 1:** Conserved *P*. *minima* miRNAs.

miRNA family	miRNA sequence	Given miRNA name	Length	Total reads	Reference miRNA	E-value
**miR156**	TTGACAGAAGATAGAGAGCAC	pmi-miR156_v1	21 nt	1898	ath-miR157a 5p	0.002
** **	TGACAGAAGATAGAGAGCAC	pmi-miR156_v2	20 nt	225	ath-miR157d 5p	0.004
** **	TGACAGAAGATAGAGAGCACTT	pmi-miR156_v3	22nt	14	ath-miR157a 5p	0.004
**miR159**	TTTGGATTGAAGGGAGCTCTA	pmi-miR159_v1	21 nt	38	ath-miR159a 3p	0.002
	TTTGGATTGAAGGGAGCTCC	pmi-miR159_v2	20 nt	29	ath-miR159c 3p	0.004
**miR160**	TTGCCTGGCTCCCTGCATGCC	pmi-miR160_v1	21 nt	23027	nta-miR160d 5p	0.004
	TTGCCTGGCTCCCTGCATGCA	pmi-miR160_v2	21nt	353	nta-miR160d 5p	0.004
	TGCCTGGCTCCCTGCATGCCA	pmi-miR160_v3	21nt	278	nta-miR160d 5p	0.002
**miR162**	GGAGGCAGCGGTTCATCGATC	pmi-miR162_v1	21 nt	2	aly-miR162a 5p	0.002
	TCGATAAACCTCTGCATCCAG	pmi-miR162_v2	21 nt	2	aly-miR162a 3p	0.002
**miR166**	TCGGACCAGGCTTCATTCCCC	pmi-miR166_v1	21 nt	290254	aly-miR166a 3p	0.002
	TCGGACCAGGCTTCATTCCCT	pmi-miR166_v2	21 nt	16666	osa-miR166m 3p	0.002
	TCGGACCAGGCTTCATTCCCA	pmi-miR166_v3	21 nt	1187	aly-miR166b 3p	0.004
	TCGGACCAGGCTTCATTCTT	pmi-miR166_v4	20 nt	298	cme-miR166i 3p	0.011
	TCGGACCAGGCTTCATCCCCC	pmi-miR166_v5	21 nt	186	ath-miR165a 3p	0.002
	TCGGACCAGGCTTCATTCCCCC	pmi-miR166_v6	22nt	53	hbr-miR166b 3p	6e10-4
	TCGGACCAGGCTTCATTGCCT	pmi-miR166_v7	21 nt	20	aly-miR166d 3p	0.008
**miR167**	TGAAGCTGCCAGCATGATCTGA	pmi-miR167_v1	22 nt	6	bdi-miR167d 5p	6e10-4
	TGAAGCTGCCAGCATGATCTGG	pmi-miR167_v2	22 nt	3	ath-miR167d 5p	6e10-4
	TGAAGCTGCCAGCATGATCTGC	pmi-miR167_v3	22 nt	2	ata-miR167c 5p	6e10-4
**miR168**	TCGCTTGGTGCAGGTCGGGAA	pmi-miR168_v1	21 nt	133	ath-miR168a 5p	0.002
	TCGATTGGTGCAGATCGGGA	pmi-miR168_v2	20 nt	15	osa-miR168a 5p	0.024
**miR169**	GGCAAGTGGTCCTTGGCTACCT	pmi-miR169_v2	22nt	25	ath-miR169a 3p	0.024
	CGGCAAGTTGTCCCTGGCTAC	pmi-miR169_v1	21 nt	19	ath-miR169a 3p	0.022
**miR171**	TTGAGCCGTGCCAATATCACAT	pmi-miR171_v1	22 nt	440	smo-miR171b 3p	0.002
**miR172**	CGAGAATCTTGATGATGCTGC	pmi-miR172_v1	21 nt	653	vvi-miR172d 3p	0.004
	CGAGAATCCTGATGATGCTGC	pmi-miR172_v2	21 nt	335	mtr-miR172a 3p	0.01
	TGAGAATCTTGATGATGCTGC	pmi-miR172_v3	21 nt	64	vvi-miR172d 3p	0.002
	GGAATCTTGATGATGCTGCAA	pmi-miR172_v4	21 nt	19	ath-miR172e 3p	0.004
	TGCAGCACCATCAAGATTCAC	pmi-miR172_v5	21 nt	25	aly-miR172b 5p	0.004
**miR319**	CTTGGACTGAAGGGAGCTCCC	pmi-miR319_v1	21 nt	1972	ppt-miR319d 3p	0.002
	TTGGACTGAAGGGAGCTCCT	pmi-miR319_v2	20 nt	1445	ath-miR319c 3p	0.004
	CTTGGACTGAAGGGAGCTCCT	pmi-miR319_v3	21 nt	113	mes-miR319h 3p	0.002
**miR390**	AAGCTCAGGAGGGATAGCGCC	pmi-miR390_v1	21 nt	9170	ath-miR390a 5p	0.002
	AAGCTCAGGAGGGATAGCGCCT	pmi-miR390_v2	22 nt	489	ath-miR390a 5p	0.002
	AAGCTCAGGAGGGATAGCGCTT	pmi-miR390_v3	22 nt	15	ath-miR390a 5p	0.004
	CGCTATCTATCCTGAGTTTCA	pmi-miR390_v4	21 nt	1	ath-miR390a 3p	0.008
**miR395**	CTGAAGTGTTTGGGGGAACTCT	pmi-miR395_v1	22 nt	2	aly-miR395e 3p	0.002
	CTGAAGTGTTTGGGGGAACTC	pmi-miR395_v2	21 nt	1	aly-miR395e 3p	0.002
	GTTCCTCTGAACACTTCATT	pmi-miR395_v3	20 nt	1	aly-miR395e 5p	0.024
**miR396**	CTCAAGAAAGCTGTGGGAAAA	pmi-miR396_v1	21 nt	414	ath-miR396b 3p	0.004
	TTCCACAGCTTTCTTGAACTG	pmi-miR396_v2	21 nt	145	ath-miR396a 5p	0.002
**miR403**	TTAGATTCACGCACAAACTCG	pmi-miR403_v1	21 nt	16	ath-miR403 3p	0.002
**miR408**	TGCACTGCCTCTTCCCTGGCT	pmi-miR408_v1	21 nt	88	smo-miR408 3p	0.002
	TGCACTGCCTCTTCCCTGGCTC	pmi-miR408_v2	22 nt	51	smo-miR408 3p	0.002
**miR477**	AGAAGCCTTTGGGGGAGAGGG	pmi-miR477_v1	21 nt	5	ppt-miR477a 3p	0.022
	CTCTCCCTCAAAGGCTTCCA	pmi-miR477_v2	20 nt	3	ppt-miR477a 5p	0.004
**miR529**	AGAAGAGAGAGAGCACAGCCC	pmi-miR529_v1	21 nt	289	ppt-miR529d 5p	0.002
	AGAAGAGGGAGAGCACAGCCC	pmi-miR529_v2	21 nt	256	ppt-miR529d 5p	0.008
	AGAAGAGAGAGAGCACAGCCT	pmi-miR529_v3	21 nt	23	bdi-miR529 5p	0.004
	GCTGTGCTCACTCTCTTCTGG	pmi-miR529_v4	21 nt	163	ppt-miR529d 3p	0.057
**miR530/1030**	TCTGCATCTGCACCTGCACCC	pmi-miR530_v1	21 nt	17663	ppt-miR1030a 5p	0.004
	TCTGCATCTGCACCTGCACCT	pmi-miR530_v2	21 nt	816	ppe-miR530 5p	0.004
**miR535**	TGACGACGAGAGAGAGCACGC	pmi-miR535_v1	21 nt	19872	ppe-miR535b 5p	0.002
**miR536**	TCGTGCCAAGCTGTGTGCAACC	pmi-miR536_v1	22 nt	26	ppt-miR536a 3p	0.002
**miR1024**	TCTGGTTGGATTGTAGGCCT	pmi-miR1024_v1	20 nt	21	ppt-miR1024a 3p	0.004
**miR1083**	TAGCCTGGAACGAAGCACGGA	pmi-miR1083_v1	21 nt	1755	smo-miR1083 5p	0.004

Reference miRNA refers to a miRBase miRNA that is most similar to a given pmi-miRNA; E-value, complementarity score between pmi-miRNAs and miRBase reference miRNAs as estimated by the BLASTN program.

For five families (pmi-miR162, pmi-miR390, pmi-miR395, pmi-miR477 and pmi-miR529), sequences that represent the putative miRNA/miRNA* duplex strands (i.e., the 5p and 3p strands of the miRNAs) were identified (S2 Table). Sixty-one percent (35/57) of the conserved *P*. *minima* miRNAs, belonging to 18 of the 23 miRNA families, have a 5' terminal uridine residue (Table 1), a conserved feature of miRNAs recognized by the AGO1 protein, while miRNAs from the pmi-miR390, pmi-477 and pmi-miR529 families have mostly a 5' terminal adenine residue, a feature found in miRNAs recognized preferentially by AGO2 and AGO4 in angiosperms [37].

All miRNAs identified in the fern *C*. *thalictroides* in a pioneering array-based survey [22] were also identified in *P*. *minima*, namely miR156, miR160, miR166, miR168, miR169, miR171, miR172 and miR390 (Table 1).

### Ancient land plant miRNA families

MiRBase contains miRNAs from well-characterized plant species for which genome information is available, like the moss *P*. *patens*, the lycopod *S*. *moellendorfii* and the angiosperms *Arabidopsis thaliana* and *Oryza sativa* [11, 13], but miRNAs coming from many recent sequencing surveys are not present in the database. To expand the scope of our phylogenetic analysis, we compared the conserved *P*. *minima* miRNAs to those of *Marsilea quadrifolia* [23], a water fern that diverged from polypod ferns like *P*. *minima* around 220 MYA [17, 24]. The miRNAs of *M*. *quadrifolia* and seed plants identified by Chávez-Montes et al. [23] were reanalysed to identify *bona fide* conserved miRNAs (see Materials and Methods).

We also compared *P*. *minima* sequences with the miRNAs of various gymnosperms and basal angiosperms [23, 38–41], as well as conserved miRNAs identified recently in the liverworts *Pellia endiviifolia* [42] and *Marchantia polymorpha* [36, 43]. This allowed us to trace the history of miRNA origin and conservation in land plants, with emphasis on the fern miRNA repertoire.

**Fig 3:**
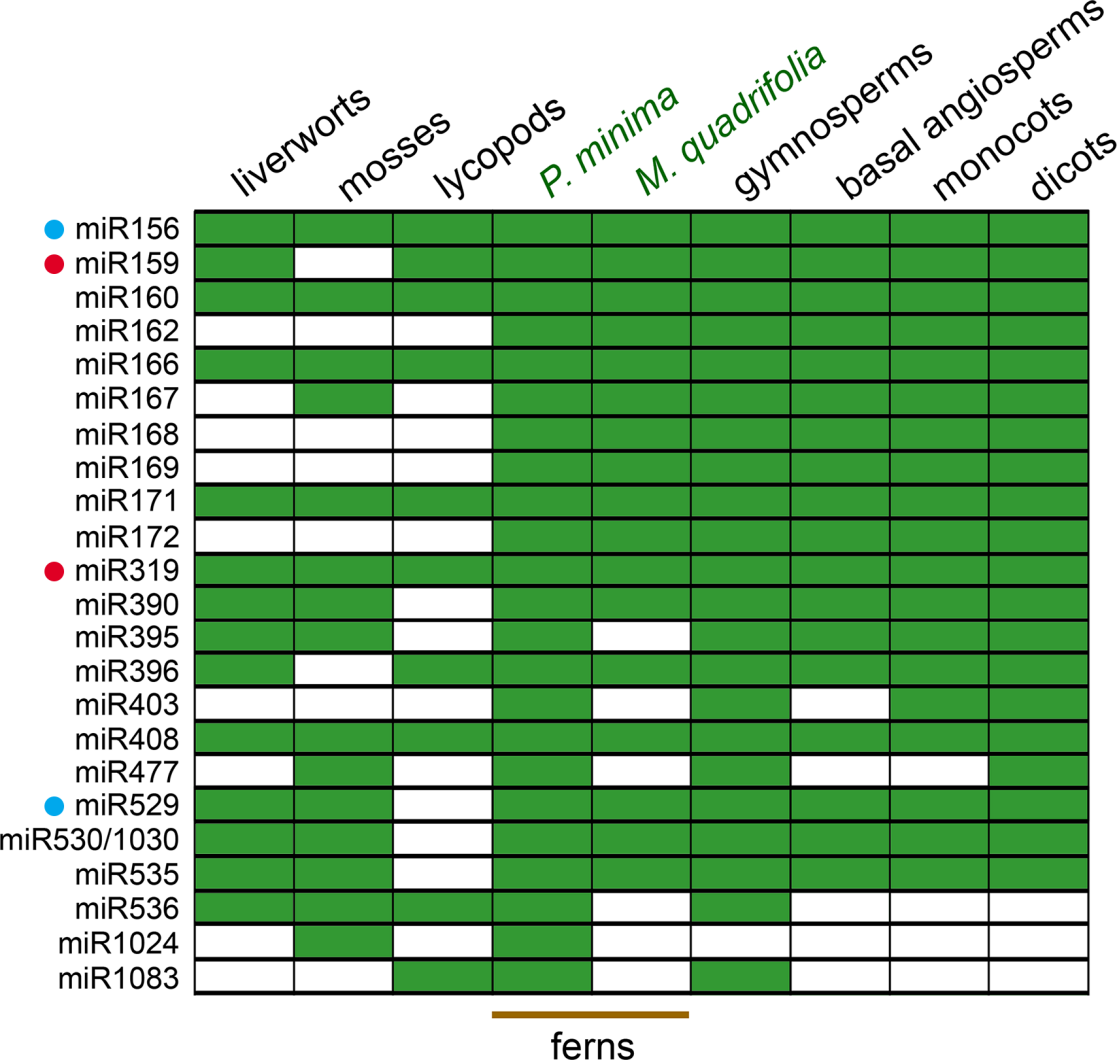
Phylogenetic distribution of conserved fern miRNAs. The grid indicates the presence (green) or absence (white) of miRNA families in liverworts (*M*. *polymorpha* and/or *P*. *endiviifolia*), mosses (*P*. *patens*), lycopods (*S*. *moellendorffii*), ferns (*P*. *minima* and *M*. *quadrifolia*) as well as gymnosperms, basal angiosperms, monocots and dicots. For gymnosperms we took into account miRNAs of *P*. *abies* [6, 41], *C*. *lanceolata* [38] and *T*. *mairei* [39] and species reported in [23]; for angiosperms we included miRNAs of species reported in [23] and in miRBase release 21.0 [31]. Pairs of miRNAs that can be considered from the same family are indicated by coloured dots. Note that miRNAs are not necessarily present in all members of a group. Among liverworts, miR530/1030, miR529 and miR536 are only found in *M*. *polymorpha*, while miR156, miR395 and miR535 are only found in *P*. *endiviifolia* [36, 42, 43]. MiR403 and miR529 are absent from most non-dicot angiosperms [44] and eudicots [32, 33], respectively.

Particularly noteworthy are miRNA families miR156/529, miR159/319, miR160, miR166, miR171 and miR408, which are found in all land plant groups, including liverworts and mosses, which represent the earliest-branching groups in land plant evolution (Figs 1A and 3). Some ancestral miRNAs have a more patchy phylogenetic distribution and are missing in a few basal groups. For instance, miR396 is absent from moss, miR167 is missing from liverworts and lycopod, while miR530/1030, miR535 and miR390 are specifically missing in the lycopod. In any case, the *P*. *minima* miRNA repertoire is conserved compared to that of basal land plant groups, since all 14 ancient miRNAs present in liverworts (*P*. *endiviifolia* and/or *M*. *polymorpha*) and all 15 ancient miRNAs of the moss, are present in *P*. *minima*. Similarly, all 10 ancient miRNAs found in the lycopod are also present in *P*. *minima* (Fig 3).

Some miRNA families are only present in a few basal groups. One is miR1024, a miRNA from the moss *P*. *patens* [13] which is found in *P*. *minima* and in no other species, not even *M*. *quadrifolia* (Fig 3). This miRNA is present in two copies in the *P*. *patens* genome and is considered to be a high-confidence miRNA, based on RNA-seq analyses and precursor structure [13, 30]. Although this miRNA has only 21 reads in our samples, it displays 100% identity over 20 nt to *P*. *patens* miR1024, indicating that it is a real miRNA. Thus, it might represent an ancient miRNA that has been independently lost from several lineages, including seed plants. Another case is that of miR1083, which is found in *P*. *minima* (but not in *M*. *quadrifolia*, see Materials and Methods) with over 1,700 reads (Table 1). This miRNA is present in the lycopod, *S*. *moellendorffii* [13], is abundant in gymnosperms [23, 39, 41], but cannot be identified in angiosperms (Fig 3; see Materials and Methods). Thus, miR1083 seems to be an ancient tracheophyte miRNA that has been specifically lost from angiosperms.

### Euphyllophyte miRNAs

We found that some miRNAs families are only common to ferns (*P*. *minima*, *M*. *quadrifolia* or both) and seed plants (gymnosperms and angiosperms), suggesting that these miRNAs originated in the early evolution of euphyllophytes. These are miR162, miR168, miR169, miR172 and miR403 (Fig 3). All of these miRNAs, except for miR403, are also found in *M*. *quadrifolia* [23], while miR172 is also found in *C*. *thalictroides* [22].

Although miR403 has been reported as being present in *M*. *quadrifolia* [23], we were unable to find this miRNA in the miRNAome of this fern and did not include it in Fig 3 (see Materials and Methods). MiR403 is an unusual miRNA in that it seems to have a very patchy phylogenetic distribution. It is usually considered to be missing in non-dicot angiosperms [44], but it has been detected in some monocots [23]. MiR403 has also been detected in some gymnosperms like the Chinese fir, *Cunninghamia lanceolata* [38], the spruce, *Picea abies* [41] and the Maire yew, *Taxus mairei* [39], although not in *Ginkgo biloba* [40]. All of this suggests a complex evolutionary history for this miRNA family, and its presence in ferns should be confirmed in the future with further RNA-seq data from other fern species and the sequencing of fern genomes.

### Conserved fern miRNA targets

Extensive experimental evidence from several angiosperms and the moss *P*. *patens*, have unveiled that the mRNAs targeted by ancestral miRNAs are often also deeply conserved [10, 13, 14, 45]. Recently, degradome data has shown that the targets of liverwort ancestral miRNAs are also partially conserved with that of other lineages [41–43]. As for ferns, the only available information consists of putative mRNA targets for *C*. *thalictroides* miR160, miR171 and miR172, which were also found to be conserved with those of other plant lineages [22].

To confirm and expand the evidence for conservation of ancestral miRNA targets in ferns, we computationally predicted the mRNA targets of conserved *P*. *minima* miRNAs. Since no *P*. *minima* transcriptome is available, we made use of the extensive transcriptome data obtained for the fern *Lygodium japonicum* [20]. *L*. *japonicum* belongs to the Schizaeales order, which places it as a distant cousin to polypod ferns like *P*. *minima* [17, 24]. We searched the *L*. *japonicum* transcriptome for potential targets of the conserved *P*. *minima* miRNA repertoire using the psRNATarget platform [46] in order to identify putative fern miRNA targets. Since functional data on fern miRNA targets is extremely limited, we restricted our analyses to targets which have been experimentally determined as targets in other plant groups.

**Fig 4:**
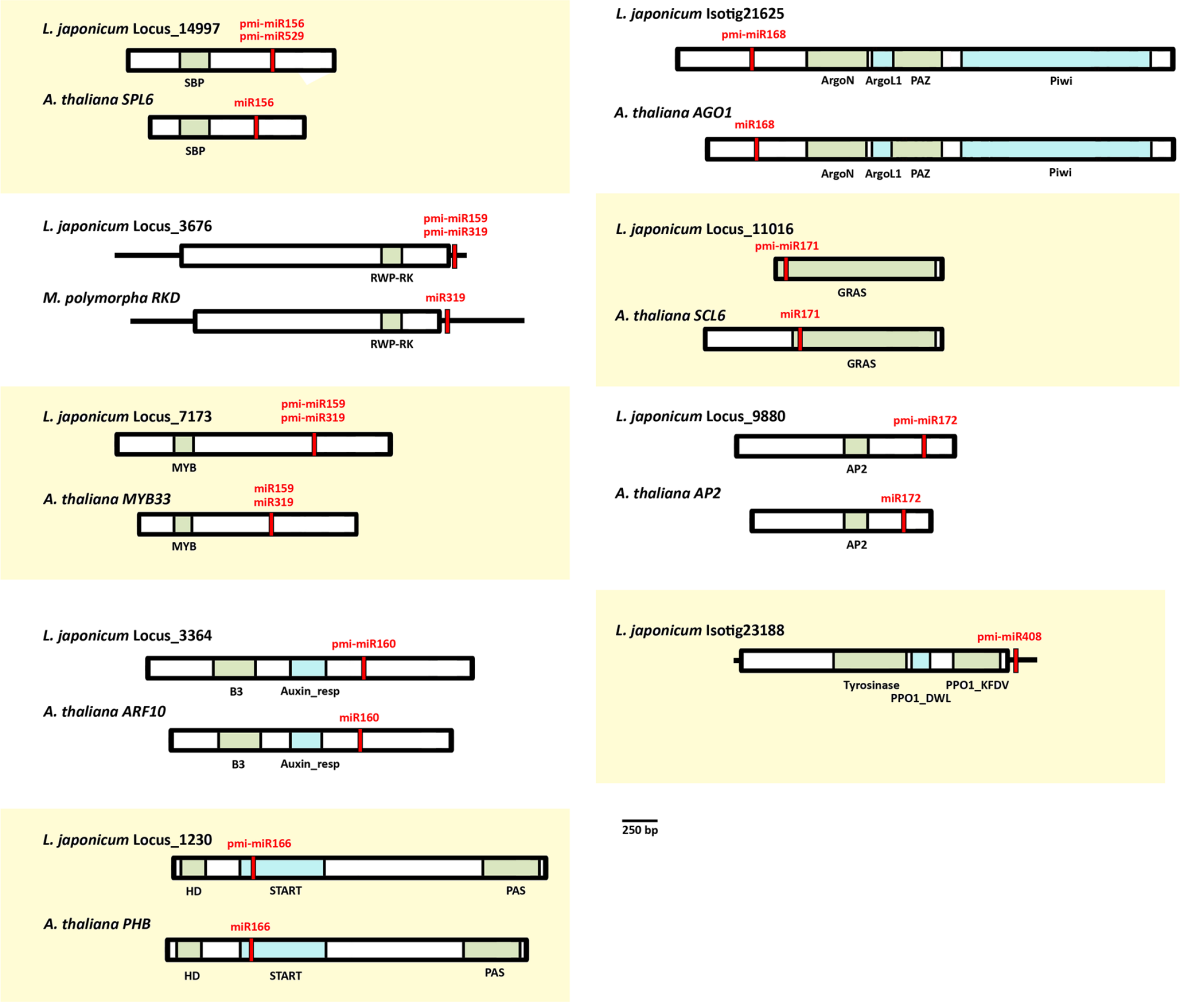
Conserved targets of fern miRNAs. Schematic representations of *L*. *japonicum* mRNAs predicted to be targeted by *P*. *minima* miRNAs. For comparison, homologous mRNAs and miRNAs from other land plants (*A*. *thaliana* or *M*. *polymorpha*) are also shown. Coding regions are shown as rectangles and UTR regions as thick black lines. Protein domains within coding regions are coloured. Regions predicted to be targeted by pmi-miRNAs are indicated by red stripes. Note that the positions of miRNA-targeted regions in mRNAs are conserved between *L*. *japonicum* and other plants. For pmi-miR408 the sequence for the homologous *PPO* mRNA from *P*. *endiviifolia* is not available and is not shown.

The analysis indicates that 11 out of the 23 *P*. *minima* miRNA families have the potential to target *L*. *japonicum* RNAs that are also targeted in other species (Table 2). Thus, pmi-miR156 and the related pmi-miR529 target a mRNA that encodes a Squamosa Promoter Binding (SPB) transcription factor; pmi-miR159 and the related pmi-miR319 target mRNAs that encode transcription factors of the MYB family; pmi-miR160 targets mRNAs encoding transcription factors of the Auxin Response Factor (ARF) family; pmi-miR166 targets a mRNA encoding a transcription factor of the class III homeodomain-leucine zipper (Class III HD-Zip) family; pmi-miR171 targets a GRAS-domain transcription factor mRNA; miR172 targets the mRNA of a Apetala-2 transcription factor (Table 2). Targets homologous to these have been observed in experimental work done in angiosperms, moss, or both [13, 14, 45]. Importantly, the *L*. *japonicum* mRNA regions targeted by pmi-miRNAs are located in the same positions as in the targets of other species, strongly suggesting that these are ancient, conserved targets of land plant miRNAs (Fig 4). In addition, sequence alignments show that the RNA regions targeted by miRNAs are strongly conserved between mRNAs of *L*. *japonicum* and homologous mRNAs from other species; indeed the miRNA-targeted elements are generally more conserved than mRNA regions that encode conserved protein domains (S1–S10 Figs). The *P*. *minima* miR160, miR171 and miR172 predicted targets confirm those of Axtell and Bartel [22] for *C*. *thalictroides*. Recently, a thorough survey found that ferns possess three clades of Class III HD-Zip genes [47], and we found that pmi-miR166 can potentially target all Class III HD-Zip paralogues of the fern *Psilotum nudum* identified in that study (S5 Fig and data not shown).

**Table 2:** Putative conserved fern targets of *P*. *minima* miRNAs.

miRNA family	*Lygodium* transcript	E	Transcript function	Target conservation
**pmi-miR156**	Locus_14997	1	SBP transcription factor	liverworts, moss, gymnosperms, angiosperms
**pmi-miR159**	Locus_7173	1.5	MYB transcription factor	gymnosperms, angiosperms
** **	Locus_3676	2	RWP-RK domain transcription factor	same target as miR319 in liverworts
**pmi-miR160**	Locus_6056	0	Auxin response factor (ARF)-B3 DNA binding domain	liverworts, moss, *Ceratopteris*, gymnosperms, angiosperms
** **	Locus_3364	1	Auxin response factor (ARF)-B3 DNA binding domain	liverworts, moss, *Ceratopteris*, gymnosperms, angiosperms
**pmi-miR166**	Locus_1230	3.0	Class III homeodomain-leucine zipper protein C3HDZ2	liverworts, moss, gymnosperms, angiosperms
**pmi-miR168**	Locus_158	4.0	Argonaute 1 (AGO1)	gymnosperms, angiosperms
**pmi-miR171**	Locus_11016	1.5	GRAS domain transcription factor (SCL6)	liverwort (*Marchantia*), moss, *Ceratopteris*, gymnosperms, angiosperms
**pmi-miR172**	Locus_9880	0.5	Apetala2 (AP2)	*Ceratopteris*, gymnosperms, angiosperms
** **	Locus_1423	1	Apetala2 (AP2)	*Ceratopteris*, gymnosperms, angiosperms
**pmi-miR319**	Locus_7173	2.0	MYB transcription factor	liverwort (*Marchantia*), moss, gymnosperms, angiosperms
** **	Locus_831	3.0	MYB transcription factor	liverwort (*Marchantia*), moss, gymnosperms, angiosperms
** **	Locus_1866	3.0	MYB transcription factor	liverwort (*Marchantia*), moss, gymnosperms, angiosperms
** **	Locus_3676	2.0	RWP-RK domain transcription factor	liverworts *(Marchantia*, *Pellia*)
**pmi-miR390**	Locus_39179	0.5	*TAS3* tasi-RNA targeting ARF3/4	liverwort (*Marchantia*), moss, gymnosperms, angiosperms
** **	Locus_20755	0.5	*TAS3* tasi-RNA targeting ARF3/4	liverwort (*Marchantia*), moss, gymnosperms, angiosperms
**pmi-miR408**	Locus_5439	3.0	Polyphenol oxydase (PPO)	liverwort (*Pellia*)
**pmi-miR529**	Locus_14997	1	SBP domain transcription factor	liverwort (*Marchantia*), gymnosperms, angiosperms

E-complementarity score between miRNA and target RNA as estimated by the psRNATarget program

**Fig 5:**
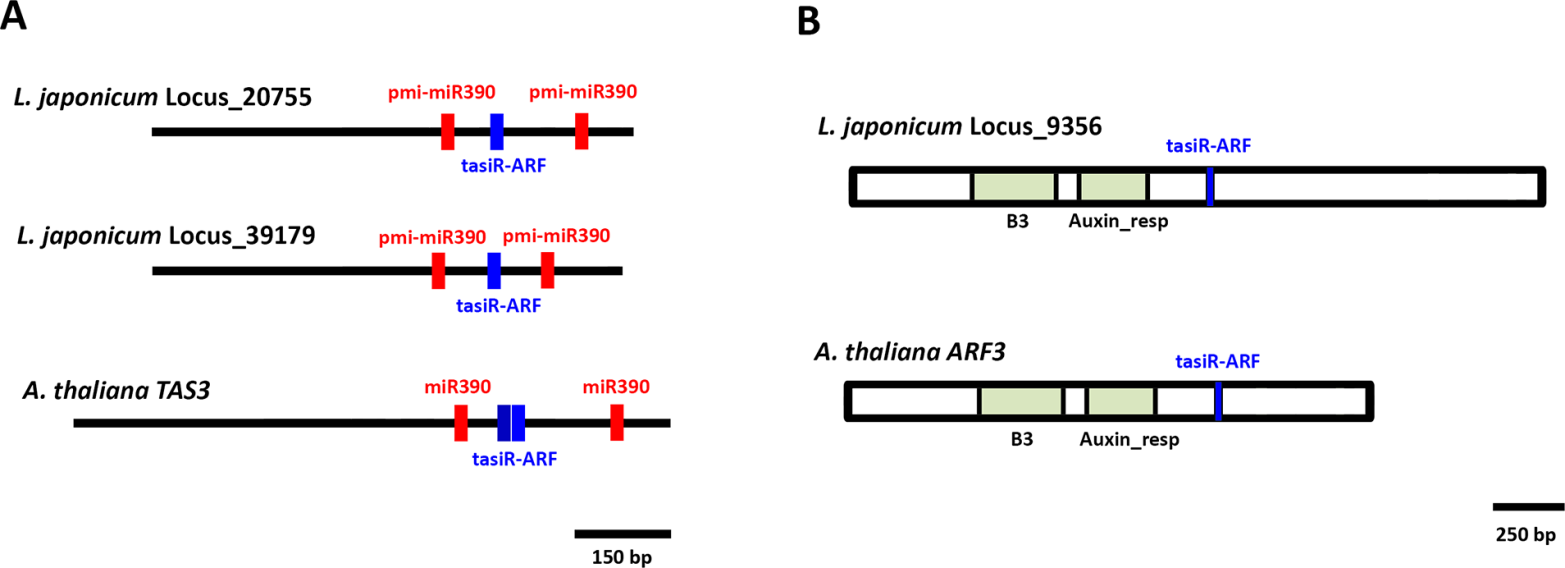
Trans-acting siRNA 3 targeting *ARF* transcripts in ferns. **(A)** Schematic representations of two *L*. *japonicum TAS3* RNAs (Locus 20755 and 39179). Predicted sites targeted by pmi-miR390 are indicated in red. The region predicted to generate a tasiRNA targeting a mRNA encoding a ARF transcription factor is indicated in blue. For comparison, a *TAS3* transcript from *A*. *thaliana* (NR_143941.1) is also shown. **(B)** Schematic representation of a *ARF* mRNA (Locus 9356) from *L*. *japonicum* targeted by tasiRNA derived from *TAS3* transcripts (blue stripe). The *A*. *thaliana ARF3* mRNA (At2g33860) is shown for comparison. Conserved protein domains encoded by the *ARF* mRNAs are indicated.

The miR390 family does not control protein-coding mRNAs, but rather targets a group of non-coding transcripts called *TAS3* RNAs (trans-acting siRNAs). In the moss *P*. *patens* and in angiosperms, two miR390 molecules hybridize to different segments of a *TAS3* RNA, which is then processed by a complex mechanism to produce one or more 21-nt ta-siRNA molecules that are incorporated into a RISC complex that targets the mRNAs of *ARF3* and *ARF4* transcription factors [48]. In addition, *P*. *patens TAS3* can also produce ta-siRNAs that target the transcription factor *AP2* [13], and there is evidence that the ta-siRNAs generated from the *TAS3* of the liverwort *M*. *polymorpha* can target *AP2* as well [36, 49]. As for the *P*. *minima* miR390 family, we found that they can potentially target two *L*. *japonicum* non-coding transcripts which exhibit characteristics of *TAS3* RNAs (Table 2, Fig 5, S9 Fig). These *L*. *japonicum TAS3* transcripts carry two sites predicted to hybridize to pmi-miR390; in addition, between the miR390 sites one potential ta-siRNA, derived from the (+) strand of the RNA, can be produced that could target *L*. *japonicum ARF3/4* mRNAs (Fig 5 and S9 Fig), indicating that the control of *ARF* genes by miR390 is conserved in ferns.

Of particular interest are some predicted *P*. *minima* miRNA targets that are conserved with liverwort miRNAs but not other plants. Specifically, apart from transcription factors of the MYB family, pmi-miR159 and pmi-miR319 potentially target a mRNA that encode a RWP-RK domain-containing protein (RKD, Table 2, Fig 4, S3 Fig). Degradome studies in the liverworts *P*. *endiviifolia* and *M*. *polymorpha* identified mRNAs encoding RWP-RK proteins as bona fide targets of miR159 and miR319 homologues of these species [42, 43]. Interestingly, the pairing between pmi-miR159/319 and its potential *L*. *japonicum* RWP-RK target occurs in the 3'-UTR of the mRNA, the same region that is targeted in the homologous liverwort *M*. *polymorpha* mRNA by miR319 (Fig 4, S3 Fig). In addition, although a RWP-RK mRNA has not yet been experimentally identified as a target for miR159/319 in moss [13, 14, 45], we found in the *P*. *patens* transcriptome a RWP-RK mRNA that harbours a potential miR159/319 target site near its 3' end, with a similar position and sequence to the RKD targets in liverworts and fern (S3 Fig). As for seed plants, mRNAs encoding RWP-RK proteins have not been identified as targets of miR159/319 in gymnosperms or angiosperms [14, 38, 39, 41, 50] and we could not find potential miR159/319 target elements in the RWP-RK mRNAs of these groups, suggesting that the miR159/319-mediated regulation of RWP-RK proteins is an ancient land plant feature that may have been lost from seed plants. Another target possibly conserved between ferns and liverworts is the mRNA encoding the enzyme polyphenol oxydase (PPO), which was identified as a potential target of pmi-miR408 (Table 2). In the liverwort *P*. *endiviifolia*, *PPO* was also identified as an experimental target of miR408 by degradome analysis [42]. Significantly, miR408 targets the 3'-UTR of *PPO* mRNAs in both ferns (Fig 4, S10 Fig) and liverworts [42].

In seed plants, some miRNAs target genes involved in the biogenesis and function of miRNA themselves. Thus, in angiosperms, miR162 targets Dicer-like 1 (DCL1), miR168 targets AGO1 and miR403 targets AGO2 [14]. In our analyses, *P*. *minima* miR162 and miR403 are not predicted to target genes similar to DCL1 or AGO2, but pmi-miR168 is predicted to target *L*. *japonicum* AGO1 (Table 2 and Fig 4). The sequence predicted to be targeted by pmi-miR168 is located within the 5' half of the *AGO1* coding region, outside the segments that encode the conserved domains of the protein, and corresponds to the homologous *AGO1* region targeted by miR168 in *A*. *thaliana* and other seed plants (Fig 4 and S6 Fig) [51]. Thus, the negative feedback loop involving miR168 and *AGO1* may be an ancestral euphyllophyte feature.

## Discussion

**Fig 6:**
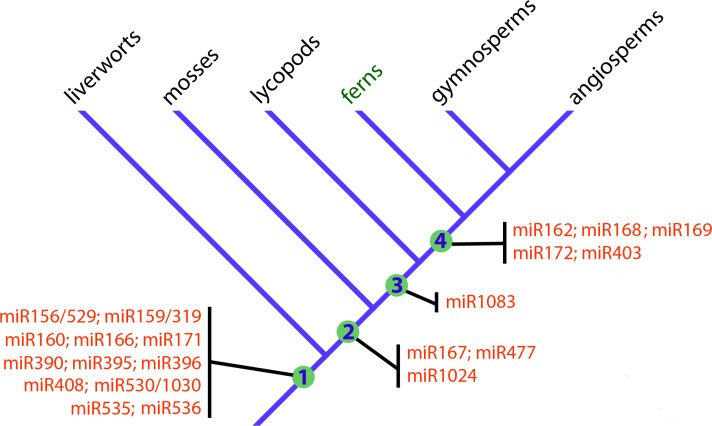
Emergence of miRNAs during early land plant evolution. The schematic cladogram shows the origin of ancient miRNA families during early land plant evolution. Only miRNA families that originated in early land plant evolution are shown, namely land plants (Embryophyta, node 1), mosses and vascular plants (node 2), vascular plants (Tracheophyta, node 3) and ferns and seed plants (Euphyllophyta, node 4). Note that, according to recent molecular phylogenies [25, 26], liverworts and mosses might belong together in a clade that is sister to vascular plants, in which case nodes 1 and 2 would be the same. Also note that five miRNA families seem to be restricted to euphyllophytes and that only miR1083 seems to have originated in the branch leading to vascular plants.

In this work, we identify the conserved miRNAs of a polypod fern, *P*. *minima*, by comparing its miRNA repertoire to those of other land plant groups. Fig 6 shows a revised scenario for the emergence of different miRNA families during early land plant evolution. Concerning the fern miRNAome, our analyses allowed us to reach a series of conclusions. First, we found that 15 ancient miRNA families that originated in the beginning of land plant evolution, as evidenced by their presence in liverworts and/or mosses, are all present in *P*. *minima*, showing that the fern miRNA repertoire is very conserved in this regard (Fig 6). This is not a trivial observation, as illustrated by the lycopod *S*. *moellendorffii*, which lacks half of the ancestral miRNAs that are present in bryophytes (Fig 3). Second, by comparing *P*. *minima* miRNAs and those of the aquatic fern, *M*. *quadrifolia*, with the rest of land plants, we identified five miRNAs that seem to have originated at the beginning of euphyllophyte evolution, before the split of the lineages of ferns and seed plants (gymnosperms and angiosperms; Fig 6). The miRNAome of *P*. *minima* seems to be more conservative than that of *M*. *quadrifolia*, since six ancestral miRNAs found in the former could not be identified in the latter (Fig 3). Third, by searching putative targets of *P*. *minima* miRNAs using the transcriptome of another fern, *L*. *japonicum*, we provide evidence that the targets of 11 fern miRNA families are deeply conserved in land plant evolution.

Molecular studies on basal land plant groups can give crucial clues on the evolution of plant form [3]. As gene expression regulators, miRNAs are essential for proper plant development and may have played important roles in the early evolutionary diversification of land plants, making comparative functional studies of ancestral miRNAs in different plant groups very interesting in this regard [10]. Some ancestral miRNAs present in *P*. *minima* exhibit an intriguing phylogenetic distribution and are interesting candidates for functional studies. One of these is miR1083, a tracheophyte-specific miRNA that is present in lycopods and is abundantly expressed in *P*. *minima* and gymnosperms, but is apparently absent from angiosperms (Figs 3 and 6). The other is miR1024, which is present in moss and *P*. *minima*, but has not been detected in any other species investigated so far (Fig 3) [13]. Neither the expression pattern nor the targets of miR1083 and miR1024 have been studied in any plant, but their antiquity and conservation make them interesting subjects for future study.

Our bioinformatic search for potential targets of fern miRNAs indicate that 11 of the ancestral pmi-miRNAs might target genes that are homologous to those of other land plants (Table 2, Figs 4 and 5). Although our evidence is based on an *in silico* approach, the conservation of potential miRNA/target pairs in species that are separated by hundreds of millions of years of independent evolution is strong evidence that these fern transcripts are real targets. Among these, we found that pmi-miR390 could target *TAS3* RNAs with the potential to generate tasi-RNAs that, in their turn, target genes encoding ARF3/4 transcription factors from *L*. *japonicum* (Fig 5 and S9 Fig). This represents the first full description of *TAS3* transcripts in ferns and an indication that miR390 function is conserved in this group, as suggested by a previous PCR-based survey [49].

Another interesting fern conserved target was found for pmi-miR166, which can target transcription factors of the Class III HD-Zip (C3HDZ) family. In angiosperms, C3HDZ genes are expressed in the adaxial side of developing leaves and are necessary for the proper dorsoventral patterning of these structures [52]. In the abaxial side, on the other hand, C3HDZ expression is repressed by miR166, which is also necessary for proper leaf patterning in angiosperms [53, 54]. Even though it is often considered that the leaves of ferns and seed plants evolved independently [3, 55], Vasco et al. [47] have recently observed that C3HDZ genes are specifically expressed in the adaxial side of developing fern leaves, just like in angiosperms, giving credence to the hypothesis that euphyllophyte megaphylls are homologous structures. Considering our observation that pmi-miR166 could target fern C3HDZ genes, future studies on the expression and function of fern miR166 may help establish the extent of molecular homology in the gene regulatory network that controls leaf development in euphyllophytes.

A recurrent feature in the evolution of plant gene regulation is that certain mRNAs encoding proteins involved in miRNA processing and function are themselves controlled by miRNAs. In angiosperms, *AGO1*, *DCL1* and *AGO2* are targets of miR168, miR162 and miR403, respectively [14], and *AGO1* is also a miR168 target in the Chinese fir, a gymnosperm [38]. Interestingly, liverworts and mosses also target *AGO1* and *DCL1* with a different set of miRNAs. Thus, miR902 and miR1047 target *AGO1* and *DCL1* in the moss *P*. *patens* [13, 56], while miR11707 targets *AGO1* in the liverwort, *M*. *polymorpha* [43]. As for ferns, we found that pmi-miR168 has the potential to target *L*. *japonicum AGO1* in a site that, given its position in the mRNA, seems to be homologous to the *AGO1* site targeted by miR168 in angiosperms (Fig 4, S6 Fig). Since miR168 originated in the lineage leading to ferns and seed plants (Fig 6), it can be hypothesized that the negative feedback loop involving *AGO1* and miR168 has been present throughout the evolutionary history of euphyllophytes. The confirmation of the miR168/*AGO1* regulatory relationship, as well as the study of miR162 and miR403 targets, constitutes an interesting avenue of research in fern regulatory RNA research.

Target prediction of *P*. *minima* miRNAs yielded two candidate targets that seem to be conserved only between ferns and liverworts. One is the gene encoding polyphenol oxydase (*PPO*), which originated in the early stages of the colonisation of land by plants and has roles in pathogen defence [57]. In both *L*. *japonicum* and the liverwort, *P*. *endiviifolia*, *PPO* is targeted at the 3'-UTR by miR408 (Fig 4 and S10 Fig). Another common potential target is *RKD*, a gene encoding a transcription factor of the RWP-RK family. In the liverworts, *P*. *endiviifolia* and *M*. *polymorpha*, *RKD* is targeted at the 3'-UTR by miR319 as revealed by degradome analyses [42, 43]. Similarly, we observed that a *L*. *japonicum* homologue of *RKD* is predicted to be targeted at the 3'-UTR by pmi-miR319 and pmi-miR159 (Fig 4, S3 Fig). Recently, it has been found that *RKD* is necessary for proper germ cell development in liverworts, as *RKD*-deficient *M*. *polymorpha* plants exhibit egg cells with aberrant cellular differentiation and proliferation properties, along with defects in gemma cup formation, which are vegetative reproductive organs [58, 59]. Work in *A*. *thaliana* indicates that *RKD* homologues might have related functions in egg development of seed plants [60]. Interestingly, the overexpression of miR319 in *M*. *polymorpha* causes defects in thalli growth and gemma cup formation in gametophytes [36]. Thus, given the important functions of *RKD*, its potential regulation by miR319 in liverworts and ferns might represent an ancient gene regulatory interaction that plays a role in gametophyte growth and germ cell differentiation in basal land plants.

Despite their ecological importance and their key phylogenetic position as sister group to seed plants, the genetic and molecular mechanisms underlying fern development and physiology are poorly known. We hope that our work will add to the growing genomic resources available to researchers dedicated to the study of this group of land plants.

## Materials and methods

### Plant material

Specimens of *Pleopeltis minima* (Bory) J. Prado & R.Y. Hirai (= *Polypodium squalidum* Vell.; = *Pleopeltis squalida* (Vell.) de la Sota.) [27] were collected in a peri-urban environment at Departamento General Alvear, Corrientes Province, Argentina (29°05'56.0"S, 56°33'12.0"W). Annual precipitation in the area reaches 17,000 mm approximately. At the time, no special permits were needed to collect plant specimens from a peri-urban environment. The ferns were found growing on branches of large trees, and a couple of specimens with the branches attached were maintained in a greenhouse at 23°C under a 16 h/8 h light/dark cycle. Field work did not involve endangered or protected species.

### RNA preparation and high-throughput sequencing

Two independent samples of *P*. *minima* fertile fronds from two different specimens each were subjected to a protocol for total RNA extraction enriched for small RNAs using the *mir*Vana miRNA Isolation Kit (Thermo Fisher Scientific). The quantity and quality of the total RNAs were analised with a NanoDrop 2000 spectrophotometer (Thermo Fisher Scientific) and an Agilent 2100 Bioanalyzer (for concentration, 28S/18S and RIN detection; Agilent Technologies). Small RNA libraries were generated from both *P*. *minima* independent RNA samples using the Illumina Truseq SmallRNA Preparation kit(Illumina, San Diego, USA). Total sRNAs were ligated to 3p and 5p adapters (ADTs), and the corresponding cDNA was obtained by reverse-transcription PCR. The purified cDNA library was used for cluster generation on Illumina’s Cluster Station and then sequenced on Illumina GAIIx (LC Sciences, Houston, USA). Raw sequencing reads were obtained using Illumina’s Sequencing Control Studio software version 2.8 (SCS v2.8) following real-time sequencing image analysis and base-calling by Illumina's Real-Time Analysis version 1.8.70 (RTA v1.8.70).

### Identification of conserved miRNAs

Initial analysis of reads were done with the ACGT101-miR bioinformatics program (LC Sciences, Houston, USA). Raw reads were filtered to remove adaptor sequences and reads which were too short (<15 nt) or too long (>40 nt). Sequences corresponding to mRNA fragments, rRNA, tRNA, snoRNA and snRNA mapping to Rfam (http://rfam.janelia.org) were removed, as well as reads mapping to RepeatMasker (http://www.repeatmasker.org/). The non-redundant sRNA reads were mapped to plant precursor and mature miRNA sequences stored in miRBase 21.0 (http://www.mirbase.org/) [31] using Bowtie software to identify an initial group of putative conserved miRNAs and precursors. Length variation (1–3 nt) at both 3' and 5' ends and up to two mismatches inside of the sequence were allowed in the initial alignments, and only sequences of 20–24 nt were selected for further analysis. Putative miRNAs identified by Bowtie were manually compared to miRBase (http://www.mirbase.org/search.shtml) using BLASTN and SSEARCH to confirm the identity of the miRNAs. The final set of conserved *P*. *minima* miRNAs (Table 1) are either 100% identical or display minor changes (one or two mismatches or 1-nt length variation) to annotated miRBase sequences. The identified *P*. *minima* miRNAs were compared to a recent re-evaluation of miRBase entries [11] to discard low-confidence miRNAs from our sample.

To better characterize the phylogenetic distribution of plant miRNAs, *P*. *minima* sequences were also checked against a large plant miRNA database generated recently [23] which includes the aquatic fern, *M*. *quadrifolia*. The miRNAs that were reported for *M*. *quadrifolia* by Chávez-Montes et al [23] were reanalysed more stringently in the following way: First, miRNA sequences for each putative miRNA family were compared against miRBase (http://www.mirbase.org/) and miRNA sequences that exhibited low similarily to miRBase entries (E-value of less than 0.01) were discarded. Next, the abundance of each miRNA sequence was checked against the Comparative Sequencing of Plant miRNA database (http://smallrna.danforthcenter.org/) [23] and sequences that were expressed at low levels (less than 1 read per million) were discarded. After this conservative reassessment, several miRNAs that seemed to be present in *M*. *quadrifolia* and other plants [23] were discarded from our evolutionary analysis. The resulting *bona fide* conserved miRNAs were incorporated into the descriptions of miRNA distribution and evolution shown in Figs 3 and 6.

### miRNA target prediction

The *P*. *minima* conserved miRNA sequences identified (57 in total; Table 1) were used to search against the transcriptome of the fern, *Lygodium japonicum* [20], for potential targets using the psRNATarget platform (http://plantgrn.noble.org/psRNATarget/) [46]. Searches were performed against the Oases_K49 database (downloaded from http://bioinf.mind.meiji.ac.jp/kanikusa/) that consists of a transcriptome assembled from sequenced RNA samples from prothalli, trophophylls, rhizomes and sporophylls obtained using Roche 454 GSFLX and Illumina HiSeq sequencers [20]. Search parameters were Expectation (E) of ≤ 3.0 (which measures sequence complementarity) and UPE of ≤ 25.0 (which measures target accessibility) [46]. For some miRNAs, namely pmi-miR168, the E parameter was relaxed (≤ 4.0) to identify further potential targets.

### Data availability

The raw sequencing data of *P*. *minima* small RNA transcriptome was submitted to the NCBI Sequence Read Archive under the accession number SRR4294182.

## Supporting information

S1 Fig Predicted targeting of a fern *SPL* mRNA by miR156/miR529. **(A)** Sequence of a transcript (Locus_14997) encoding a Squamosa-binding protein-like (SPL) homologue from the fern *L*. *japonicum*. The region predicted to be targeted by pmi-miR156 or pmi-miR529 is indicated in yellow. The starting ATG and stop codon are highlighted in blue. **(B)** Alignment of part of the *SPL* transcripts from *L*. *japonicum* (Lja), the liverwort *M*. *polymorpha* (Mpo), the gymnosperm *Pinus tabuliformis* (Pta), the dicot *A*. *thaliana* (Ath) and the monocot *O*. *sativa* (Osa). Residues displaying over 75% identity are highlighted. The region targeted by miR156/529 is indicated in red, and the conserved SBP-DNA binding domain in green. Note that the miRNA-targeted region is conserved in all mRNAs and species. **(C)** Predicted pairing between pmi-miR156 and miR529 and *L*. *japonicum* Locus_14997. The E-complementarity score between miRNA and target RNA as estimated by the psRNATarget program is shown.
(DOCX)

S2 Fig Predicted targeting of fern *MYB* mRNA by miR159/miR319. **(A)** Sequence of a transcript (Locus_7173) encoding a MYB transcription factor protein from the fern *L*. *japonicum*. The region predicted to be targeted by pmi-miR159 or pmi-miR319 is indicated in yellow. The starting ATG and stop codon are highlighted in blue. **(B)** Alignment of part of the MYB transcripts from *L*. *japonicum* (Lja), the liverwort *M*. *polymorpha* (Mpo), the moss *P*. *patens* (Ppa), the gymnosperm *Pinus tabuliformis* (Pta), the basal angiosperm *Amborella trichopoda* (Atr), the dicot *A*. *thaliana* (Ath) and the monocot *O*. *sativa* (Osa). Residues displaying over 75% identity are highlighted in blue. The region targeted by miR156/529 is indicated in red, and the conserved MYB DNA binding domain in green. Note that the miRNA-targeted region is conserved in all mRNAs and species. **(C)** Predicted pairing between pmi-miR159 and miR319 and *L*. *japonicum* Locus_7173. The E-complementarity score between miRNA and target RNA as estimated by the psRNATarget program is shown.
(DOCX)

S3 Fig Predicted targeting of a fern *RKD* mRNA by miR159/miR319. **(A)** Sequence of a transcript (Locus_3676) encoding a RKD transcription factor protein from the fern *L*. *japonicum*. The region predicted to be targeted by pmi-miR159 or pmi-miR319 is indicated in yellow. The starting ATG and stop codon are highlighted in blue. **(B)** Alignment of part of the RKD transcripts from *L*. *japonicum* (Lja), the liverwort *M*. *polymorpha* (Mpo) and the moss *P*. *patens* (Ppa). Residues displaying over 75% identity are highlighted. The region targeted by miR159 (or miR319) is indicated in red, and the conserved RWP-RK DNA binding domain in green. Stop codons are encircled in yellow. Note that the miRNA-targeted region is conserved in all mRNAs and species. **(C)** Predicted pairing between pmi-miR159 and miR319 and *L*. *japonicum* Locus_3676. The E-complementarity score between miRNA and target RNA as estimated by the psRNATarget program is shown.
(DOCX)

S4 Fig Predicted targeting of a fern *ARF* mRNA by miR160. **(A)** Sequence of a transcript (Locus_3364) encoding an ARF transcription factor protein from the fern *L*. *japonicum*. The region predicted to be targeted by pmi-miR160 is indicated in yellow. The starting ATG and stop codon are highlighted in blue. **(B)** Alignment of part of the ARF transcripts from *L*. *japonicum* (Lja), the liverwort *M*. *polymorpha* (Mpo), the lycopod *S*. *moellendorffii* (Smo), the moss *P*. *patens* (Ppa), the gymnosperms *Picea abies* (Pab) and *Cycas rumphii* (Cru), the dicot *A*. *thaliana* (Ath) and the monocots *O*. *sativa* (Osa) and *Brachypodium distachyon* (Bdi). Residues displaying over 75% identity are highlighted. The region targeted by miR160 is indicated in red, and the conserved Auxin response domain (ARF) in green. Note that the miRNA-targeted region is conserved in all mRNAs and species. **(C)** Predicted pairing between pmi-miR160 and *L*. *japonicum* Locus_3364. The E-complementarity score between miRNA and target RNA as estimated by the psRNATarget program is shown.
(DOCX)

S5 Fig Predicted targeting of a fern *C3HD-Zip* mRNA by miR166. **(A)** Sequence of a transcript (Locus_1230) encoding an C3HD-ZIP transcription factor protein from the fern *L*. *japonicum*. The region predicted to be targeted by pmi-miR166 is indicated in yellow. The starting ATG and stop codon are highlighted in blue. **(B)** Predicted pairing between pmi-miR166 and *L*. *japonicum* Locus_1230. The E-complementarity score between miRNA and target RNA as estimated by the psRNATarget program is shown. **(C)** Alignment of part of the C3HD-ZIP transcripts from ferns *L*. *japonicum* (Lja) and *Psilotum nudum* (Pnu), the liverwort *M*. *polymorpha* (Mpo), the lycopod *S*. *moellendorffii* (Smo), the moss *P*. *patens* (Ppa), the gymnosperms *Cunninghamia lanceolata* (Cla), *Taxus globosa* (Tgl) and *Gingko biloba* (Gbi); the basal angiosperm *Amborella trichopoda* (Atr), the dicots *A*. *thaliana* (Ath), *Medicago truncatula* (Mtu), *Solanum lycopersicum* (Sly) and *Populus trichocarpa* (Ptr), and the monocots *O*. *sativa* (Osa) and *Brachypodium distachyon* (Bdi). Residues displaying 100% identity are highlighted in blue. The region targeted by miR166 is indicated in red, the START domain in green, and HOX Homeodomain in light blue. Note that the miRNA-targeted region is conserved in all mRNAs and species.
(DOCX)

S6 Fig Predicted targeting of a fern *AGO1* mRNA by miR168. **(A)** Sequence of a transcript (Isotig_21625) encoding AGO1 protein from the fern *L*. *japonicum*. The region predicted to be targeted by pmi-miR168 is indicated in yellow. The starting ATG and stop codon are highlighted in blue. (B) Alignment of part of the AGO1 transcripts from fern *L*. *japonicum* (Lja), the gymnosperm *Cunninghamia lanceolata* (Cla), the dicot *A*. *thaliana*, and the monocots *O*. *sativa* (Osa) and *Brachypodium distachyon* (Bdi). Residues displaying 100% identity are highlighted in blue. The region targeted by miR168 is indicated in red, and the Argonaute N-terminal domain domain (ArgoN) in green. Note that the miRNA-targeted region is conserved in all mRNAs and species. (C) Predicted pairing between pmi-miR168 and *L*. *japonicum* Isotig21625. The E-complementarity score between miRNA and target RNA as estimated by the psRNATarget program is shown.
(DOCX)

S7 Fig Predicted targeting of a fern *SCL* transcription factor mRNA by miR171. **(A)** Sequence of a transcript (Locus_11016) encoding a Scarecrow-like(SCL)/GRAS domain transcription factor protein from the fern *L*. *japonicum*. The region predicted to be targeted by pmi-miR168 is indicated in yellow. The transcript is incomplete and lacks the starting ATG, but the stop codon is highlighted in blue. **(B)** Alignment of part of the SCL transcripts from fern *L*. *japonicum* (Lja), the liverwort *M*. *polymorpha* (Mpo), the lycopod *S*. *moellendorffii* (Smo), the moss *P*. *patens* (Ppa), the gymnosperms *Pinus tabulliformis* (Pta) and *Pinus radiata* (Pra); the basal angiosperm *Amborella trichopoda* (Atr), the dicots *A*. *thaliana* (Ath), *Solanum lycopersicum* (Sly) and *Populus trichocarpa* (Ptr), and the monocots *O*. *sativa* (Osa) and *Brachypodium distachyon* (Bdi). Residues displaying over 75% identity are highlighted. The region targeted by miR171 is indicated in red, and the GRAS domain in green. Note that the miRNA-targeted region is conserved in all mRNAs and species. **(C)** Predicted pairing between pmi-miR171 and *L*. *japonicum* Locus_11016. The E-complementarity score between miRNA and target RNA as estimated by the psRNATarget program is shown.
(DOCX)

S8 Fig Predicted targeting of a fern *AP2* transcription factor mRNA by miR172. **(A)** Sequence of a transcript (Locus_9880) encoding a Apetala-2 transcription factor protein from the fern *L*. *japonicum*. The region predicted to be targeted by pmi-miR172 is indicated in yellow. The starting ATG and stop codon are highlighted in blue. (B) Alignment of part of the AP2 transcripts from ferns *L*. *japonicum* (Lja) and *Ceratopteris thalictroides* (Cta), the gymnosperms *Pinus tabulliformis* (Pta) and *Ginkgo biloba* (Gbi); the basal angiosperm *Amborella trichopoda* (Atr), the dicot *A*. *thaliana* (Ath, and the monocots *O*. *sativa* (Osa) and *Brachypodium distachyon* (Bdi). Residues displaying over 75% identity are highlighted. The region targeted by miR172 is indicated in red, and the AP2 DNA-binding domain in green. Note that the miRNA-targeted region is conserved in all mRNAs and species. **(C)** Predicted pairing between pmi-miR172 and *L*. *japonicum* Locus_9880. The E-complementarity score between miRNA and target RNA as estimated by the psRNATarget program is shown.
(DOCX)

S9 Fig Predicted targeting of fern *TAS3* RNAs by miR390. **(A)** *TAS3* RNAs (Locus 20755 and Locus 39179) from *L*. *japonicum* predicted to be targeted by pmi-miR390. The regions predicted to pair with miR390 are indicated in blue, and the trans-acting small-interfering RNAs (tasi-RNAs) derived from *TAS3* processing are shown in yellow. The tasi-RNAs are predicted to target mRNAs encoding ARF3/4 transcription factors. **(B)** *TAS3* RNAs were aligned with the CLUSTAL Omega program. Regions predicted to be targeted by pmi-miR390 are indicated in blue, with the base pairing with miR390 indicated. Note that the pairing between pmi-miR390 is more extensive at the 5' site than at the 3' site, indicating that RISC-mediated cleavage of the RNAs probably occurs only at the 5' site. The tasiRNAs derived from *TAS3* processing are shown in yellow. **(C)** Alignment of part of the *TAS3* transcripts from *L*. *japonicum* (Lja) and a *TAS3* from the dicot *A*. *thaliana* (Ath). Residues displaying 100% identity are highlighted. **(D)** Sequence of a transcript (Isotig23398) encoding a ARF transcription factor protein from the fern *L*. *japonicum*. The region predicted to be targeted by pmi-tasiR-ARF is indicated in yellow. The starting ATG and stop codon are highlighted in blue. **(E)** Alignment of part of ARF transcripts from fern *L*. *japonicum* (Lja), the gymnosperm *Pinus pinaster* (Ppi); the basal angiosperm *Amborella trichopoda* (Atr), the dicot *A*. *thaliana* (Ath) and the monocot *Brachypodium distachyon* (Bdi). Residues displaying 100% identity are highlighted. The region targeted by pmi-tasiR-ARF is indicated in red, and the B3 DNA binding and the Auxin responsive factor domains are also indicated. Note that the tasiR-ARF-targeted region is conserved in all mRNAs and species.
(DOCX)

S10 Fig Predicted targeting of a fern *PPO* mRNA by miR408. **(A)** Sequence of a mRNA encoding Polyphenol oxydase (PPO, Locus 5437) from *L*. *japonicum* predicted to be targeted by pmi-miR408. The targeted region is located in the 3'-UTR of the mRNA (yellow). The starting ATG and stop codon are highlighted in blue. **(B)** Predicted pairing between pmi-miR408 and *L*. *japonicum* Locus_9880. The E-complementarity score between miRNA and target RNA as estimated by the psRNATarget program is shown.
(DOCX)

S1 Table Statistics of sRNA libraries. (XLSX)

S2 Table *P minima* putative 5p and 3p miRNAs duplexes. (XLSX)
