# Supplementary material for: Sequencing of small RNAs of the fern Pleopeltis minima (Polypodiaceae) offers insight into the evolution of the microrna repertoire in land plants
Source: PLoS One. 2017 May 11;12(5):e0177573. doi: 10.1371/journal.pone.0177573 (PMC5426797; doi:10.1371/journal.pone.0177573)
Supplement: S1 Fig — (A) Sequence of a transcript (Locus_14997) encoding a Squamosa-binding protein-like (SPL) homologue from the fern L. japonicum. The region predicted to be targeted by pmi-miR156 or pmi-miR529 is indicated in yellow. The starting ATG and stop codon are highlighted in blue. (B) Alignment of part of the SPL transcripts from L. japonicum (Lja), the liverwort M. polymorpha (Mpo), the gymnosperm Pinus tabuliformis (Pta), the dicot A. thaliana (Ath) and the monocot O. sativa (Osa). Residues displaying over 75% identity are highlighted. The region targeted by miR156/529 is indicated in red, and the conserved SBP-DNA binding domain in green. Note that the miRNA-targeted region is conserved in all mRNAs and species. (C) Predicted pairing between pmi-miR156 and miR529 and L. japonicum Locus_14997. The E-complementarity score between miRNA and target RNA as estimated by the psRNATarget program is shown. (DOCX) [file pone.0177573.s001.docx]

**Fig S1. Predicted targeting of a fern SPL mRNA by miR156/miR529.**

**(A)** Sequence of a transcript (Locus_14997) encoding a Squamosa-binding protein-like (SPL) homologue from the fern *L. japonicum*. The region predicted to be targeted by pmi-miR156 or pmi-miR529 is indicated in yellow. The starting ATG and stop codon are highlighted in blue. **(B)** Alignment of part of the SPL transcripts from *L. japonicum* (Lja), the liverwort *M. polymorpha* (Mpo), the gymnosperm *Pinus tabuliformis* (Pta), the dicot *A. thaliana* (Ath) and the monocot *O. sativa* (Osa). Residues displaying over 75% identity are highlighted. The region targeted by miR156/529 is indicated in red, and the conserved SBP-DNA binding domain in green. Note that the miRNA-targeted region is conserved in all mRNAs and species. **(C)** Predicted pairing between pmi-miR156 and miR529 and *L. japonicum* Locus_14997. The E-complementarity score between miRNA and target RNA as estimated by the psRNATarget program is shown.

**(A)**

>Locus_14997_Transcript_4/6_Confidence_0.471_Length_2284

CCCTCCGAGCACCCGTCGTAGGTAGGTACCGGTACCGCCAGACGAAGTTGCCCCTGTTTTGGGGATAAGAGTACTCCAGCACTCCAAAGTACTGCTGCCCATGTTCTTCTAGCGACATTTGAGCAAAGTGGGCTTTTGCAAATCCGCAATCTTTTTTTTTGGGTGCTAGGGGTTTGTTGGAGATTATTATGCACATGCATGGCCTGGCATTCCACTAAGCCTTCTTGCCCAAATTGGGGGTGGGCAGACAGCAGAGGGGGAGGAGAAGGAGCAGGAGGAGGAGGAGGATCAGAAGAAGTCCTCTTTCTGAACAGCCACCATGGCAGGGGCGCCCCTGACAATAACATCCTCGCCGCACCACCGGCTGCAAAACCTCCGGTGGCCCGGGATGCCCTTGCCCAGCAGCCTCAGCGGAATGCATCATGGATCCTCCACCAAGCCCATGGCCCCTCGCTTTATGCGGGAAATGGTGGTGATGGCCCTGACACAGACGTTTGCAATGGACATGGCTTGGATGCGGGCATCAATGCCGCCTTTGCAGCTGCCTCGCCGTCGACAACATTAGCTGTTGGCGGGCATGGTGATCCTAGCAGTAACCTTCCTTTCTGGGATTATGGTGCATCTCACGGCACTAGCAAAGACTGGTGTGCTGGGTCTAAAATCGGCATTCTTGATTTCAAGCATGATAATGCACATGGAGTTGTTGTTAGTGAGCATGATGTTGTTGATGCCAAAGAGAATGCTCTGAAAGAGGGAGATATAGAGTTTCTAAAGCTCAGAAGCCATCATGGCTCAAGTTCTCTTGGACAGTGGAAGCCTGTGTCTACAGGCACTCAGCAACAAGGCAGCCCTATTGTGGAAGAAGTCAATGGGAAGCAGTCACTGCCATTTTTACCCCATGCTGGAGCTTTCCATGACACTTCAGCCACGCAATCCTCTGAAGAGCAGCATAGTAGCACAGCAACTGGTTCAGCTGGGTCTGGGGACTCCCTAACTGGTCTGCATCTGGGAAAAAGAAACCATGATGGTGATGCTACAGTCAGCACCGTGCCAAGAAGGGTCCAAAATCCTGCCCAAAGTAACCCAGTTCCTTCGAAGAAGCAGCGAGCTAGCTCAGTTGCTCTGCAAACACCGCGCTGTCAGGTACAAGGGTGCAACCTGGAGCTCACAAATGCCAAAGCCTACCACAAGAGACACAAAGTCTGTGATGCCCACTCCAAAGCCGAGAAGGTAGTAGTTGCAAATCTGGAGCAACGCTTTTGCCAGCAGTGCAGCAGATTTCATGTTTTAGCTGAGTTTGATGAAGTTAAAAGAAGCTGCAGGAAAAGGCTCGCAGGGCATAATGAGCGTCGTAGGAAGCCACAACCTGAAGCCTTAGCTTTGAATGCACGCTTGTCATCCATCTTTCGAGATTGGCCTTTCTATCCTCAGTCAAGGCTTACAGGAATGTCATCTTTATGGGGTGATCCTGGAGAGTCATCGTCCCTGTTTTTCAGAGGCAGCTGGCCAAAGACTGTAAAGTTGGAAGACCAGCAAGGCCACAATTGGGCCATGCAGGCTTCAGGGATAGAGAAAGAAATGTTTTACACAGGTTCCTCGCCTCACTTCGGTGAGAAGTCACCACTACTGCTCCTTCATAGTCCAAAAGGGATGATGTCAATGCCAAATGACTCACAAGGCCAAAGGTTTTCGCAGTACTTGCCAAGATCCTTTGGCCATGGGGCTGATGGTTCAACTTTGTCATCTTCATCTGGAGCAGGGGCGCTCTCGAGCCTGGAAGCAGCACCAGCTACTCAGGCTGTTACTCGAGTCACAGACTCTGGGTGTGCTCTCTCTCTTCTGTCAGTGCAGACACAGGGCTCTCATGTTGCAGATGCAGTGTCTTTGGGTTTGTCAACTGGGGCCAGCCTACCAGTTGATTCATCAATGAATGATAACATATTGTCTATAGCTTTCAATTCTCGGAAGGACTTGGGGGGTGGAAGTAATGGTGGAATGGAAAAGGACCAGATTGTAAACTCATTCATCCACGGTTTCAATGGTGTTGGCTGCTTACAAAGCCTGCCCTTGTATCCGACGCTACGTGGTCAGGAGACAAAGGTCCTTCAAGCATCAAGGTCAGCAGTAGAGGTCATGCAAATAATGCCTCTATCTCAACCGTATGGACAAGCAATGGGATTTCAAATGCCTTTTGGAGACTTGCCATATTCTGATGAATCTTCGTTGATGGAACTGCAGCAAAGGATCTAGGCAGTATATGCCTTAATGACATAAGGACCCAAAT

**
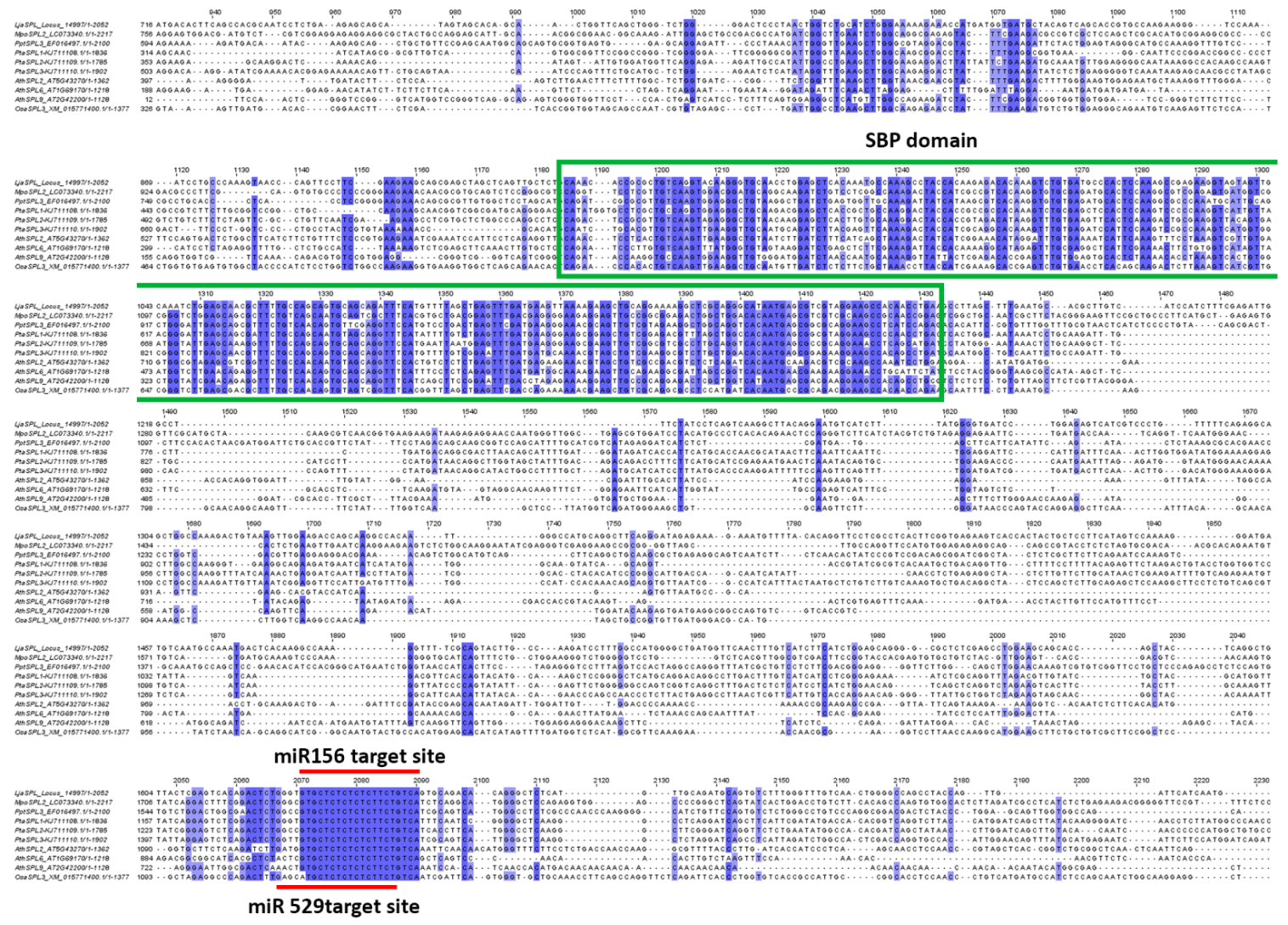
(B)**

**(C)**

(E)

**pmi-miR156v1** 21 CACGAGAGAUAGAAGACAGUU 1

::::::::: ::::::::::. 1.5

**Lja_Locus14997** 1826 GUGCUCUCUCUCUUCUGUCAG 1846

**pmi-miR529v** 120 CCGACACGAGAGAGAGAAGA 1

:: ::::::::::::::::: 1.0

**Lja_Locus14997** 1822 GGGUGUGCUCUCUCUCUUCU 1841
