# Supplementary material for: Sequencing of small RNAs of the fern Pleopeltis minima (Polypodiaceae) offers insight into the evolution of the microrna repertoire in land plants
Source: PLoS One. 2017 May 11;12(5):e0177573. doi: 10.1371/journal.pone.0177573 (PMC5426797; doi:10.1371/journal.pone.0177573)
Supplement: S2 Fig — (A) Sequence of a transcript (Locus_7173) encoding a MYB transcription factor protein from the fern L. japonicum. The region predicted to be targeted by pmi-miR159 or pmi-miR319 is indicated in yellow. The starting ATG and stop codon are highlighted in blue. (B) Alignment of part of the MYB transcripts from L. japonicum (Lja), the liverwort M. polymorpha (Mpo), the moss P. patens (Ppa), the gymnosperm Pinus tabuliformis (Pta), the basal angiosperm Amborella trichopoda (Atr), the dicot A. thaliana (Ath) and the monocot O. sativa (Osa). Residues displaying over 75% identity are highlighted in blue. The region targeted by miR156/529 is indicated in red, and the conserved MYB DNA binding domain in green. Note that the miRNA-targeted region is conserved in all mRNAs and species. (C) Predicted pairing between pmi-miR159 and miR319 and L. japonicum Locus_7173. The E-complementarity score between miRNA and target RNA as estimated by the psRNATarget program is shown. (DOCX) [file pone.0177573.s002.docx]

**Fig S2. Predicted targeting of fern MYB mRNA by miR159/miR319.**

**(A)** Sequence of a transcript (Locus_7173) encoding a MYB transcription factor protein from the fern *L. japonicum*. The region predicted to be targeted by pmi-miR159 or pmi-miR319 is indicated in yellow. The starting ATG and stop codon are highlighted in blue. **(B)** Alignment of part of the MYB transcripts from *L. japonicum* (Lja), the liverwort *M. polymorpha* (Mpo), the moss *P. patens* (Ppa), the gymnosperm *Pinus tabuliformis* (Pta), the basal angiosperm *Amborella trichopoda* (Atr), the dicot *A. thaliana* (Ath) and the monocot *O. sativa* (Osa). Residues displaying over 75% identity are highlighted in blue. The region targeted by miR156/529 is indicated in red, and the conserved MYB DNA binding domain in green. Note that the miRNA-targeted region is conserved in all mRNAs and species. **(C)** Predicted pairing between pmi-miR159 and miR319 and *L. japonicum* Locus_7173. The E-complementarity score between miRNA and target RNA as estimated by the psRNATarget program is shown.

**(A)**

>Locus_7173_Transcript_2/8_Confidence_0.667_Length_2531

ACTATGATGATCTATAGTTCTTCTTCCGACTTCTTATTACTACATATAATGCCTATGAACCCTTCTCGACATCTGATGAACCCTTCTCGACATCTGTTGCTTATGTTCAGACATATTATGGCTTATATGTTTTGTAATGCTGTTATTTTGCACTCTCAAGCCAGCCAAGGTAGCTTCCTTGTTTTTTGAAGCAACACTCCCTCAAACAGCTGTAAACTTTTTTTTGATATATTTTAATACAACTTTAAAGCTTCCGTCAAAAAAAAAAAAAGAGAGGGGGGGCATATATAGGCGTGCAGGCAAAAGGAGCGGTAGAAAGTACAATGGAAAAGAGCAGCGGGGGCTACATGATGCTGCATGCGATGCAGCAACTACAACAGCAGCCAAGGCAAGCTGGGATGCAGGGATCAGCTGGGTACACCTCTAATGGACATGCGACGTCGAGATCGGTATCGTTATCTTCGTCGTCATCGACATCTTCTTCTTCGTCGCAGCCTCATGAACGTCGTCGTCAGGAGGAGGAGGAGGAGCAGCAGCAGCAAGGAGGAGGAGGAGGAGGGCTAGGGCTAGGGCTAGGGCTGAAGAAGGGGCCGTGGAGCGCGGCGGAGGACAAGGAGCTGATGGAGTACGTGCGGAAGTACGGGGAGGGGAACTGGAACGCGGTGCAGGCGTGCTCGGGGCTGCGCCGCTGCGGCAAGAGCTGCCGCCTGCGCTGGACCAACCAGCTCCGCCCGGACCTCCGCCGTGACCGCTTCACCCCCGCCGAGGAGGCCCGCATCGTCCTCCTCCAGTCCATCCACGGCAACAAGTGGGCCCGCATCGCAGCCGAGTTCCCTGGCCGCACCGACAACGCCATCAAGAACCTCTGGAACACTCGCAACAAGCGCCTCAAGCGCCAGGCCGCCGCTGCCGCTGCTCAGTCTTCCTCCCCCTCCTCGTCAACCACAGCTATCAGGAGCAGCTGTAGCTTGCATGCCGCAAACCGGGCGTCTCGCCTCACTAAGACTCTCGGAGCTTCATTCACTATTAACGCGGCCACCTTCTCTCCTGCCAATTGGAGCTGCCAAACGCAACAATCCATTAACCTAAACAGTACAGGACGGGCCAACATTGTGATCAACCCCCCTGCCTTAACCACCAGTTGTACAGCGCTATCTTTCCCAGGAGCTGCTGCTGCGAACCAAAGCGACCCATTGTTCGCTTCTCTCTATTCTCTCTATGCAGGCCAGCCAACGCGCTCACTGTCAAGGGAGCTGGGTCGACCTTTTCAAGCGCGTAATCATCATTTGAACACAAAGCTAACCGCTTCTGATCAATATCCTTTTTTATCTTCGCCACCGACGGCTTCCAAGGCCGGCAACCGTGTACAAAACGAGAGCCAGCAAAATGCATTCACAGTTGCTGAATTAGCTGAAGGGAACGACAGATGGGGGAATCTCCCCTCTGGTTTGTTTTACGAGGGTTTAGCATCAGACCCAGGCCCCTCTAGTAATGCTCAAGATGACGCCACTGCAGCAGCCCGAGGTCTGAGTACACTGGCCGCCGCAGCCGCCGCCGCCGCCTTCAATCCAGCGAGCATTTCTGATACTGGTCATGACCATACTTTTCCTTTAACATCGGCATCACCAGCCAGCAGCCAGTCGAAGCTGGATCTCCCTTCAGTCCAAACGTCTTCATGTGACCATAATGGTATGACGAATGCCATCACCAATACTAAACCAGCTCATAATAAAACAATTGCTGCCCGTAGCCATATCAGCCATCAGCTTCAGGATTCAACCGGTCAACCGGGGGTCTTTTTAGCTGACAAAATGCATTACACTTATCAGCTAGAACCAGCAGCTCCACATCCCATCAATAAATTAACCGCAGATTCTAATCCGGAGCTGGTCACAAGCTGCAGCAGCCTCTTCCCGGATCATAAGGCACAGAAGCAGAATATATTGTTTACTGATCCTCTGGCACTTCTAGGAGGCAAGTCTCTGTCCATTTTAACCGAGGATAGCTACAATATCTCTTCTCTAATTGGTTTGCAGGCGTTCGATCATGTGTCAACTCCCTCCACGGGTGATCAGGAGCACAGTATGGCCGACGCCAGTAATGGTGTTGAGTTGCAGCAATTATATCCAGCTTCTACGGCAGGTAGCTCTTCATGCGATGACGGCAGTGCAGAGTATGGGGGCCTGGAGAGTATGTTCAAACTCATGCAGTGGACCACCATGCCGGGGAGCTGTCATATTGCGGACTCGCCAGCCGCAGCTGTTGATCAGTTGAACTCGCCCCAATCTAATTAGTATTCTGTCCCTTCACATTATTGCATGCATGCAACGTACATTCACGTCTTAAGGTTGCCAGCGTCACTGGTTATGCTTTGTCTTTCGGGTTTCTCCTCATGGCCATACTTTAATTCTTTAAAATCTCCTCGATTTTAGTTTGATCACTACTTGTCACCTTAGGTCCTGAACTATCGTCTTAATACGTTTAATTACTTCAATAGTTTTGCCGCTCAAGGAATTACCAGGAGAGAAACA


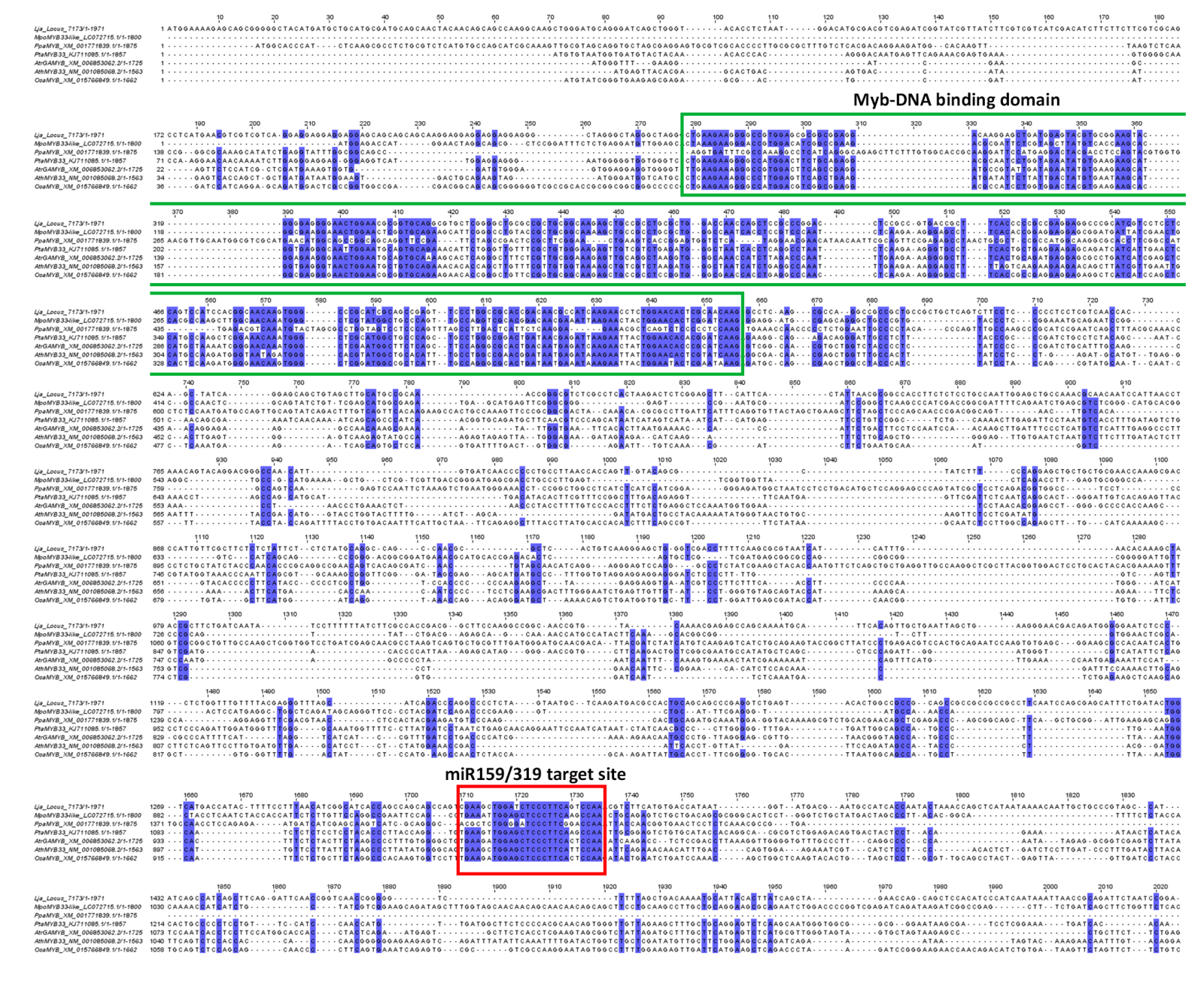
**(B)**

**(C)**

(E)

**pmi-miR159v2** 20 CCUCGAGGGAAGUUAGGUUU 1

::: :::::::::.:::::: 1.5

**Lja-Locus_7173** 1649 GGAUCUCCCUUCAGUCCAAA 1668

**pmi-miR319v1** 20 UCCUCGAGGGAAGUCAGGUU 1

::: ::::::::::::::: 2.0

**Lja-Locus_7173** 1648 UGGAUCUCCCUUCAGUCCAA 1667
