# Supplementary material for: Sequencing of small RNAs of the fern Pleopeltis minima (Polypodiaceae) offers insight into the evolution of the microrna repertoire in land plants
Source: PLoS One. 2017 May 11;12(5):e0177573. doi: 10.1371/journal.pone.0177573 (PMC5426797; doi:10.1371/journal.pone.0177573)
Supplement: S3 Fig — (A) Sequence of a transcript (Locus_3676) encoding a RKD transcription factor protein from the fern L. japonicum. The region predicted to be targeted by pmi-miR159 or pmi-miR319 is indicated in yellow. The starting ATG and stop codon are highlighted in blue. (B) Alignment of part of the RKD transcripts from L. japonicum (Lja), the liverwort M. polymorpha (Mpo) and the moss P. patens (Ppa). Residues displaying over 75% identity are highlighted. The region targeted by miR159 (or miR319) is indicated in red, and the conserved RWP-RK DNA binding domain in green. Stop codons are encircled in yellow. Note that the miRNA-targeted region is conserved in all mRNAs and species. (C) Predicted pairing between pmi-miR159 and miR319 and L. japonicum Locus_3676. The E-complementarity score between miRNA and target RNA as estimated by the psRNATarget program is shown. (DOCX) [file pone.0177573.s003.docx]

**Fig S3. Predicted targeting of a fern RKD mRNA by miR159/miR319.**

**(A)** Sequence of a transcript (Locus_3676) encoding a RKD transcription factor protein from the fern *L. japonicum*. The region predicted to be targeted by pmi-miR159 or pmi-miR319 is indicated in yellow. The starting ATG and stop codon are highlighted in blue. **(B)** Alignment of part of the RKD transcripts from *L. japonicum* (Lja), the liverwort *M. polymorpha* (Mpo) and the moss *P. patens* (Ppa). Residues displaying over 75% identity are highlighted. The region targeted by miR159 (or miR319) is indicated in red, and the conserved RWP-RK DNA binding domain in green. Stop codons are encircled in yellow. Note that the miRNA-targeted region is conserved in all mRNAs and species. **(C)** Predicted pairing between pmi-miR159 and miR319 and *L. japonicum* Locus_3676. The E-complementarity score between miRNA and target RNA as estimated by the psRNATarget program is shown.

**(A)**

**>LjaRKD_Locus_3676 _Transcript_5/5_Confidence_0.529_Length_2469**

TAGTAGTCGTCAGTAGTATTATTAGTATTAGCCTCTCTCTTCTCTTCTAGGGAACCCATCATTCTCTCCACACTTTCTTTCCTCTCTCTCTCTCTCTGTCTCTCTCTCTCTCTCTCTATGTCCTGATCTGTTCCATCAATCCTTCCCCTTTCATCACCCTCACCCTCCTCTAGATTGAGCCCTGGATTGTGCCCCCTACCCCCCCAATCAAGCTCTCCTCCCTTCTCCTTCTGTCCCTTTGCCAATGCCCCTGTCCTGATCATCACATTTAGACCCCCCCATCTCAGGGGGCAGGGAGGAAGAGAGGAGGGGAGGGGGGCTACCCATATTTGTATGTACCAATAATGGTGATCAATCACATCATCAGTCACTGCAGTTTGCGCTAATTTCAGCTCCTTTTTTTTTTTGGGCTCCTCTGTCACATAACCCATAAAAAGGGCATCAATCAAGGTCCAGCTTCTATGGCTGAATCCCAAAACCTTCTGGCTCTTGCTGTCTTCAAAGACACTCACAAAGCAGAGTATGTGAGAAGCATCCACATGTATGATCAGAACGGGATGATGATGACCACCCAACAGGAGTTTCTTCTCACTCCGGGGTTGTACTCGAGAATAAGCCCACCCGCGCCTTGTGGGTACTTCTGCTCTGCTGCGTCCTCGTCTGCGGCGAATGTCAAGTCAGCCATGGTGGCCATTAAGAAGATGCTTGAGGGGGACAGATGGAAATGTGTGCTGGAGTTTTTGAAGCATTGGCCCCATGAAATCACAATCTTAGATCCTCTCCTGCTAATCCCCTCAAGGAATGAAAAGTTAAAGAGCATCCCTTCTTTAGCCCAGGACCTTGTGCTGGTGGAAGTAGCATATAACAACCACAGCAGCAACCTTAATGCGAGCCCCGGCATTTTGGTCCCTGATCATGTCAATAGCACTAGTATTAGCACTTCAAAGACATCACCACGGGTCCCAAATATCAATGAACTGGACACCTGCCAAGATGCTAGCTGTGATATTCCAAGGTTATCAATCTTCTCAGGAGAGCCATTAAGTGATCAGATTCCTGGGTTAGAGACACCGGATAGGACTCCTCAAGGTGCCCAAGCTGATATCTGGCAAAAATGGGGCTCAGATTTAGAGCTTGCAAGTCCACGTGTCAATTATCGAGAGCTGGGGTCATGGAGGACCAATTATCCAGACTCAACTCAAGTGCCTCTTAATTCTACCAGTCCGACTGCAGCAGACCCTCCTCCGCCTTGGAGCCCTTCGCTGCTGGATCTGAGTTTGCACTTGCCAGACTTGCTTTCAGAACCCCGAGAGGCCAACACTTCTCTCCTACTATCCAGTTCCTTTGGACCAGCAACAGGCCTCAACCTTGAATTGGGAACATCCTTGAGCCTACAAAACTCCACCATTACACAAGCTGAAGCTAAAGATCAAGGACAAGAGGACACCACAGGCATCGATTGCTTCTCCTTGGATTGCTTTGGCCTGGACAGCTCCTCAAGCAATTTCTACTCTGGTGAGATAACAAATCGCATCGCAAGATCTCTTTGCAGTGATGACAGCATTGGAACAGGCCAAGTTCTGGGCTTGCGGTCCACTTCAGAGTTTAGCAGCCACGATCTCACATATGATATGAATGATGTGTTTCACACAGACAAGGCAAATTCTTCTACCACAACCTCGAGTGATGTGCATTGGGGTGGAGGAGGCAAGGGTGTGAAGCTGTCTTCTGGGGAGCCCTATCAGTTGGAGTCTCCTGCCACCTCAACTGGTTACACGAACTGGAGAGGAGGTAGGGAAGGCAGGGTGACTCGCAGGGGACGGCAAGCTGAGAAAAGTATGTACTTGCAGCAAAAGGGTACGAGTGAGCGGATAACTGAGATCACTCTGCAAGAACTCTCTCAATACTTTAACATGCCCATAACCCAGGCTTCCAAGGAGCTGAAAGTAGGGTTAACAGTTTTGAAGAAGAGATGCAGGGAATTTGGAATACCACGCTGGCCTCACCGCAAGATGAAAAGCCTTGACAGTCTTATCAACAACATTCAGGATTTGGCACGCGATCAAGGCGTGAAGAGCTCTGCAAGGGTGCTCAATGCAGTAAAGGAGTTGGAGGAGCAGAAGAGGCTAATGGAGGAGTGTCCTGGGACGGAGCTTGCCGAGCGGACGAAAAGGCTAAGGCAAGCTTGTTTCAAGGCAAGCTACAAGAAGAGAAGGCTGCAGGCACAAATTCAACAAGCTCAAGGCAATAACATGAATGCTAATCCTAATCACAATAACAACCATGCCTTCCTCCTAAATCATCTAAACCCTACTCCCACTTCTTCAGTTGACACCGAGTACGACCTCGTGTGCCCCAAACTCTAGCGTAGGGGCTCCCTTCCGTCCAAAGGAGAAAAAAAAATCAGACTTTTTTTTCCCCTCCCCTAAATCTGTTTTTTGCCAGAATCTCAGAACCCAACTGTGAATT


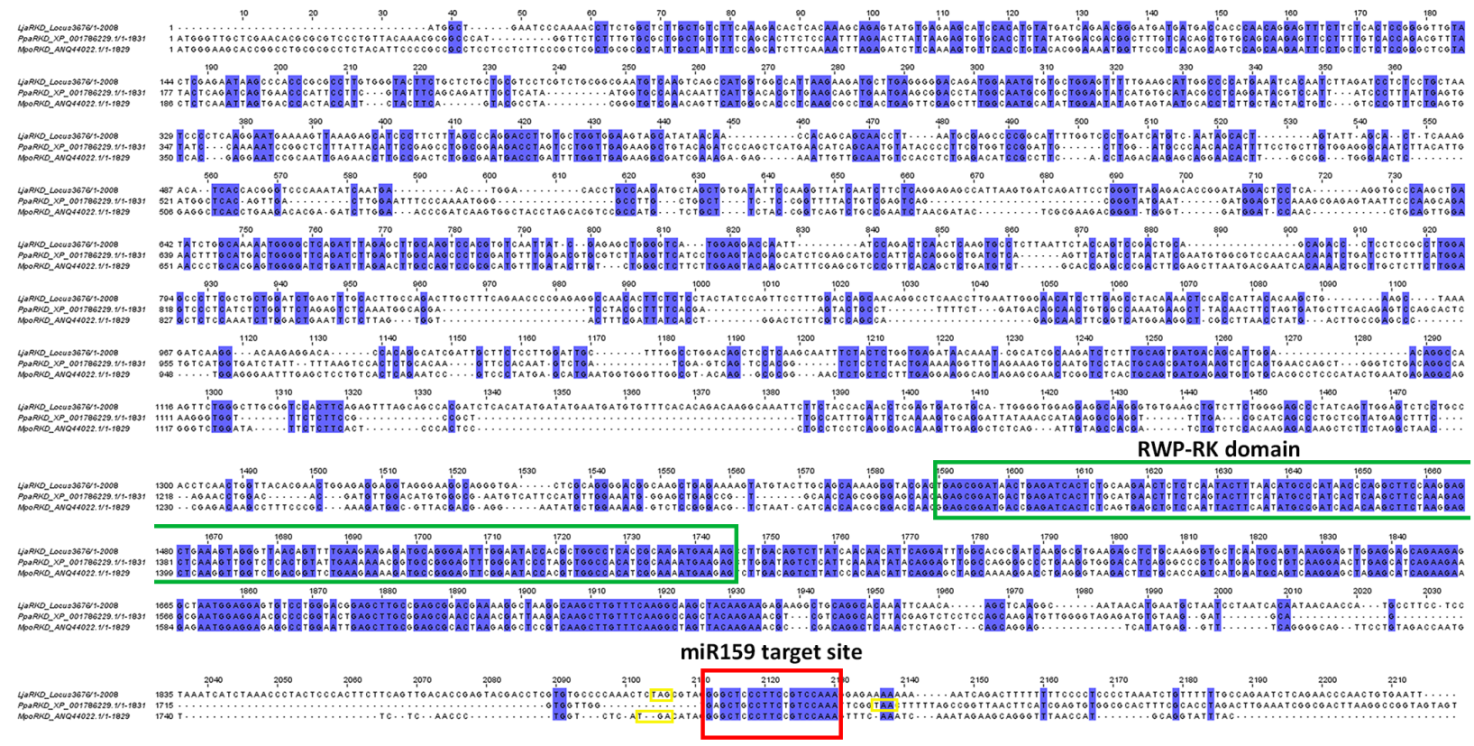
**(B)**

**(C)**

(E)

**pmi-miR159v1**  20 CCUCGAGGGAAGUUAGGUUU 1

::.::::::::: .:::::: 2.0

**Lja-Locus_3676** 2371 GGGGCUCCCUUCCGUCCAAA 2390

**pmi-miR319v1** 20 UCCUCGAGGGAAGUCAGGUU 1

:::.::::::::: :::::: 2.0

**Lja-Locus_3676** 2370 AGGGGCUCCCUUCCGUCCAA 2389
