# Supplementary material for: Sequencing of small RNAs of the fern Pleopeltis minima (Polypodiaceae) offers insight into the evolution of the microrna repertoire in land plants
Source: PLoS One. 2017 May 11;12(5):e0177573. doi: 10.1371/journal.pone.0177573 (PMC5426797; doi:10.1371/journal.pone.0177573)
Supplement: S4 Fig — (A) Sequence of a transcript (Locus_3364) encoding an ARF transcription factor protein from the fern L. japonicum. The region predicted to be targeted by pmi-miR160 is indicated in yellow. The starting ATG and stop codon are highlighted in blue. (B) Alignment of part of the ARF transcripts from L. japonicum (Lja), the liverwort M. polymorpha (Mpo), the lycopod S. moellendorffii (Smo), the moss P. patens (Ppa), the gymnosperms Picea abies (Pab) and Cycas rumphii (Cru), the dicot A. thaliana (Ath) and the monocots O. sativa (Osa) and Brachypodium distachyon (Bdi). Residues displaying over 75% identity are highlighted. The region targeted by miR160 is indicated in red, and the conserved Auxin response domain (ARF) in green. Note that the miRNA-targeted region is conserved in all mRNAs and species. (C) Predicted pairing between pmi-miR160 and L. japonicum Locus_3364. The E-complementarity score between miRNA and target RNA as estimated by the psRNATarget program is shown. (DOCX) [file pone.0177573.s004.docx]

**Fig S4. Predicted targeting of a fern ARF mRNA by miR160.**

**(A)** Sequence of a transcript (Locus_3364) encoding an ARF transcription factor protein from the fern *L. japonicum*. The region predicted to be targeted by pmi-miR160 is indicated in yellow. The starting ATG and stop codon are highlighted in blue. **(B)** Alignment of part of the ARF transcripts from *L. japonicum* (Lja), the liverwort *M. polymorpha* (Mpo), the lycopod *S. moellendorffii* (Smo), the moss *P. patens* (Ppa), the gymnosperms *Picea abies* (Pab) and *Cycas rumphii* (Cru), the dicot *A. thaliana* (Ath) and the monocots *O. sativa* (Osa) and *Brachypodium distachyon* (Bdi). Residues displaying over 75% identity are highlighted. The region targeted by miR160 is indicated in red, and the conserved Auxin response domain (ARF) in green. Note that the miRNA-targeted region is conserved in all mRNAs and species. **(C)** Predicted pairing between pmi-miR160 and *L. japonicum* Locus_3364. The E-complementarity score between miRNA and target RNA as estimated by the psRNATarget program is shown.

**(A)**

>Locus_3364_Transcript_2/10_Confidence_0.578_Length_3958

ATATCCGATCTTCTTCTGAAACCCGGAAAATACGAACAGCCAGCAGAGTCTCTCTCTCTTTTATTCTGCCCACCGGGTAGATTTTGCCATTTGCCATAGATTGCTCATCAGCACCTCCCCTGCCATGGCACCTTTCCGGCTCCCCCTCCTAGTTTCCTGACGCATCCACAGCTTATCATTCGTCTCTGCCTCTGTCTCTCTCTCTCTTTCTCTGTGTCTTGCCCTGTTCTTGCAGCAAGACAGATCGCCCTGGCTACCCCCCCCTTGTCGTCTGGGATGCCCGGGCCCTTCTCAATGAGCGTCAACAGCCTCAAGATGGACCATGGCGGAGACGACCACCGTAGCCCGTGGGCAGGAGCAGCCTTGCGCAAGCGCAGTGCCGACCCGGCGCCCGCAACCAACAACGGCCACCACCATAGCAGCGCCAGCATCGGCGGCGGTGGTACCGGGCTGGATTCTCAGCTGTGGCATGCCTGCGCCGGAGCCATGGTGCACCTCCCTAGGGTGGGAGCCAAGGTGATCTACTTCCCACAGGGCCACGCAGAGCACGCTGCATCCCCGCCCGACTTACCGCCATCCCTCGCCCTTAACAGCACTGTCCCTTGCCGTGTACTCTCGGTGCAGTTCCTCGCCGACATCGAGACCGACGAGGTTTATGCCCGCATCCGCCTGAAGCCCGACCCTGTGGTGGCTTCTGATCCATTAGAGGATTCCCCACCGTCCTCCCCTCTGCCTGAGAAATCAGCCTCATTTGCTAAAACCCTAACCCAGAGTGATGCCAATAATGGTGGTGGCTTCTCCGTCCCTCGCTACTGTGCTGAGACCATCTTCCCCCGGCTGGACTACACTGAGGATCCGCCTGTTCAGATTGTGCATGCAAAGGATGTTCATGGTACTGTGTGGAAGTTCCGGCACATCTACCGTGGCACACCACGGCGTCATCTCCTTACCACTGGCTGGAGCAACTTTGTTAACCAGAAGAAACTTGTTGCTGGTGATGCCATTGTGTTCTTGAGAAGTGCTGGAGGGGAGCTTTGTGTCGGTGTGCGGCGATCTACCAGGGGAACCAGTGCAGGGGATTCTTCTTCTTGGCATATTTCGGCAGCAGGTGTGCGATCTTCGTCGAGGTGGGAGCTGAAGCCTCAAGATAATTCATATGATTTCCTGGGGCCAGCAGAAGGTAACCCAACTTTAACAAGCTTGAATCGTGCCATTAGAGCAAGCACAGACACTGGCACCACTACCAGCAACTTTGCTAGGAACCGTGCTCGGGTTACTGCCAAATCTGTCATGGAAGCGGCTATGCTGGCTGCAGAGGGGCAGCCATTCGAGGTGGTCTACTATCCACGGGCTACTATTTCAGAATTCTGTGTGAAAGCATCTGCAGTTAAGGCAGCCTTACAGCAAAGCTGGGCCCCAGGGATGCGTTTCAAGATGGCAGTAGAAACGGAGGATGCATCTCGAATTAGTTGGTTTATGGGGACCATTGCCAAAGTGCAAGAGGCTGACCCTGTACACTGGCCCAATTCTCCTTGGAAGATGTTGCAGGTGACATGGGATGAACCAGACCTGCTTCAGGGCATTGTACGAGTGAGTCCATGGCAAGTGGAGCTTGTATCTCCAATGCAGCTGCCACCATTTGCTTTGCCAAAGAAAAAATTGCGGCTTTCGCAACCACCAGAGCTCCAACTTGATGGCCAGGGCATGATGGGGCTTACGATGGCAGCGCTTGCCAGCAATATGCTGGGGCATATTAACTCTTGGCATGGGTATGTGGACAGTGTTCCTGCAGGCATGCAGGGAGCCAGGCATGATCGTAACTTTGGGCTAGCATTTCAGGACTTTCAACCCAACATGTCCCAACCAGGTTTGTTCTTGGAGAGTTTTTATGAGCACCAAGACAATCCTATGCTGGCTGGTACTAGGGTGTCAACTGAGCTTAATATGGGTAATTTTGCTCATCAGAGCAACTTGGGTGTCCAAAACCAACTCTCCACTTTGTTGACAGTTGGAAGTTCCAGTAGTTCTGAGCCTGCATCAAGCAGTAGTGTATTGAATTGGGATAAAAGTGGAAGCTCCAGCACCAAGAACGCACCCTTTCTACTCTTTGGAAAGGCGATCGACACCAGTCAATCAATAAAATCACACCCCACTCAACTGTCTGGTGGCAGCATTTCAGATGGCCATGGACACGAGACCATGTCAGATGGAAATCAATCCGAAGTGCTTGATAAGATGCATGGGTGCATTACAGGACATGGGAAAAGATCAAATACCTTACAACAGCAATGGGAAAGTTTTGAGTCCACTTCCAATGAAGTTGGAACTCTTGATTGGTTCCGAGAACAGGCAAGCATTCTTGACAGAGACCGAGCCTTGAATAATGTTGACAATGCCTTCAACCATTGCAAAGTGTTTCGAGAAAGCGATGAGGTTGGGCGAACTGTAGATCTTTCTGTATTTGACTCATATGAGGAGCTTTATGAGAGGCTGTCAGCCATGTTTGGTGTTCAAAAACTTGAGATTCTGAATCGGGTGGTGTGTGTTGACTCTAGGGGTTCTTCAAGACCTGTTGGTGAGGAACCATACAGGTAATTCTTCGCACTCAGCTGACATTATGCATTACTGCAAGCATGGGGGGATGATCTGTGTTATTGGAAGGCTGTTCTGTTATTGGAAGCTGTTCTTAATAAGCTGACATGGAGTCCATCAGCATTGCTGCACACACACACATTGCTGCAAACATGGGGGTCATGTGTCTTGTCTGTCCTAATACATGATTAAGGTGCAAATTAATGCTTTGTTAACATTTTTGCAGGGAATTTGTCAAACGTGTGAAGAGGCTGAAGATACTATCAGAGTCCAGCAGTGAGAGTATGACAAGGTGACTGAAAGCAATGGCTTGCCAACCAACATAACATGGCTGGGCCAGTTGATGGGCCTCTGCTTCTATTGGTTGGAAAGATGTGATTTGTGGTTTCAATTTTCCAAAACTTTTTTTTCCGAAGTGATGTCACACAAAGTCGAGGCAAATGCCCCTATGTTTCGACAAGTTCAAAGGTTCAATGCAAACTGTCATAACCTCTCTCTCTCTCTCTCTCTCTCTCGTGTACATCTTGCGTTGGCTGTCTATGCATTTGGGCCTTTTTTGGATTCAGCTCAAAATTGGTAAATTTGGAAGCATCTCTCTCTCTCTCTCTCTCTCTCTCTCTCTCTCTCTCTCTCTCACAGAGACTGTGTGTGTGTGTCTGTGTCTGTATATGTCTCTCGCTCGCTTAAAAGGGCAGGAAAGCATGATGCTAAGGTCAGGGCTGGTGAGGACAGCCACCTGTCCGAGGGCACAAGACATTACGGAGCGGCTGAGGGCGAGGAACAGGCAGCTTGAGAAGAGCGTGGAGAGCCTCCAGGGAACGCTGAGGGCCAAGGAGGCGGAGGCCGAGGCCGAGAAGCGGGAGCTGAGGGAGAGGATAAGGGCCCTCGAGGAGGCCGTGCGCGAGGCTCATCGCAAGCACACCGAGGCCGAGTGCGGCCTAGAGACCGAGTTCAGCAAGGTCATGGAGGAGATCCGCCACCGGCTCACGGAGGCCGATAGGGCGCGCGCCGAGGGCCTCTCGCAGCTGCAGCACCAGCTCCAGCAGAAGCACCACCTCATCGCCGGCCTCCACAACGAGGCCCGTACCACCATCGCCAGCTTGTTGCAGGCTGCTTCTGCTTCTGCTTCTGCCCCTCAACCCTAATTCAATACTGCTGCCTGCTGATTGTCCCCCTCAGCCCTAATTCATTCTAACTTGCTGTTGCCCTAATTCATTACTTACTACACCACATTGGAATTGTTTACTTTCTATATCATCCCGACCAAAAATTGGAATTGAAATACAAGGCTAACTACATGTGTATAATTGATTTCTCGTACTACTCACGAATAATTCGTTCATAACAGTACCTGCCCATATTCGTC


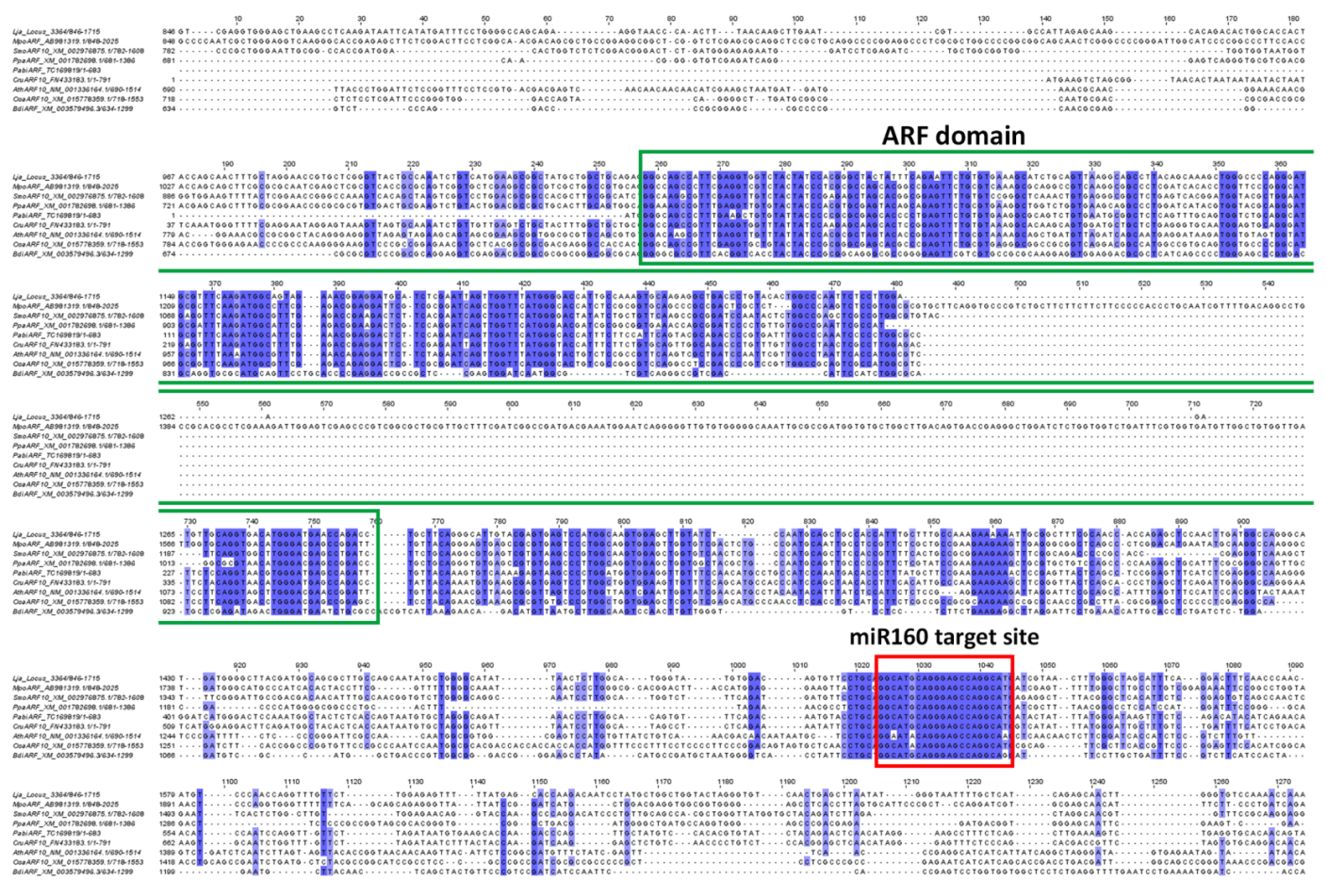
**(B)**

**(C)**

**(E)**

**pmi-miR160v1** 21 CCGUACGUCCCUCGGUCCGUU 1

:::::::::::::::::::: 2.0

**Lja-Locus_3364** 1792 GGCAUGCAGGGAGCCAGGCAU 1812
