# Supplementary material for: Sequencing of small RNAs of the fern Pleopeltis minima (Polypodiaceae) offers insight into the evolution of the microrna repertoire in land plants
Source: PLoS One. 2017 May 11;12(5):e0177573. doi: 10.1371/journal.pone.0177573 (PMC5426797; doi:10.1371/journal.pone.0177573)

**Fig S5. Predicted targeting of a fern C3HD-Zip mRNA by miR166.**

**(A)** Sequence of a transcript (Locus_1230) encoding an C3HD-ZIP transcription factor protein from the fern *L. japonicum*. The region predicted to be targeted by pmi-miR166 is indicated in yellow. The starting ATG and stop codon are highlighted in blue. **(B)** Predicted pairing between pmi-miR166 and *L. japonicum* Locus_1230. The E-complementarity score between miRNA and target RNA as estimated by the psRNATarget program is shown. **(C)** Alignment of part of the C3HD-ZIP transcripts from ferns *L. japonicum* (Lja) and *Psilotum nudum* (Pnu), the liverwort *M. polymorpha* (Mpo), the lycopod *S. moellendorffii* (Smo), the moss *P. patens* (Ppa), the gymnosperms *Cunninghamia lanceolata* (Cla), *Taxus globosa* (Tgl) and *Gingko biloba* (Gbi); the basal angiosperm *Amborella trichopoda* (Atr), the dicots *A. thaliana* (Ath), *Medicago truncatula* (Mtu), *Solanum lycopersicum* (Sly) and *Populus trichocarpa* (Ptr), and the monocots *O. sativa* (Osa) and *Brachypodium distachyon* (Bdi). Residues displaying 100% identity are highlighted in blue. The region targeted by miR166 is indicated in red, the START domain in green, and HOX Homeodomain in light blue. Note that the miRNA-targeted region is conserved in all mRNAs and species.

**(A)**

>Locus_1230_Transcript_6/8_Confidence_0.500_Length_4100 CGAAAAGTTCCCTCTCGTCTTCTCTCTTCTCTCTCTCTGTCTCTCTCTCTCTCTCTTTTCTGATCCTGCCAAAAGAGTCCTACCCACTTTCCTTCCTGGACAAGTGCGCGCTCGCCTTCCTTGCGGTTTTCTTTTGAGCCTCTCTCTATCTCACACACACACACACTCTCTTTCTCTTTCACCCTCCCTCTCTTTGGCTCCCATTCCATCTCTTCTCCTTCTTCTCCTTCTAATAGTCCTGCCTCGGGTCTTGGCAAACGCCTTGATGTTAGCCATACACACTGCCCCATAATACCCCTTGTGTTTTTTCTTTATCCTCTCCCTCGAAAGAAAAACACACCTCTCCAGAAAGGACACACACATAGCTAGAGCGCGCATGTATATTCGGACACTTCCCTTCTTTGCGCGTGCGAACCATTCGTCCCTATTTCCTCTCTCTCTGTCTCTCTCTGTCTCTCTCTAGACTGTCTGCATGAGTATATGCGTCTTGGGCTGATCTCTATCATCACACCACCTTGTCTGCCATCGCCTTCTCTCTGCTCTATAACCAAGCCGCTTGCTCGCTGCAGCTGTCCTGATCATGTGAGCCCTCGCTGTCATGGCTGCGGCCCTCTCTCTCTTCCCACTCACTAGCTGCTCCAGCACCTGCCTGCATATTGAGCTCCCATCCTCAAATTAGTTAATTAGCAGACACTCACTCATTACCCGAGTCGCCTTATATCAGCTACTCAATAGCCTGATAATCCAATCAGCAGAAAAAGGAATCATTTCAGGCAATCACTCACTCACTCACACACGCTCTCTCTCTCTCTCTCTCTCGCTGTCACATATGTTGACGATGTCTGGGAGTAGCGTGATCTCGAAAGAGAAATGCTCACTCGACTCGGGCAAGTATGTGCGTTACACGAATGAGCAAGTCGAGGCTTTAGAGCGCTTATACAATGAGTGCCCGAAGCCGAGCTCCCTTCGGAGGCAGCAGCTCATCAGAGAGTGCCCGATCCTCCAAAACATCGAGCCCAAGCAAATCAAAGTGTGGTTCCAAAACAGGAGGTGTCGCGAGAAGCAGCGAAAGGAGTATGCTCGGCTGCAAGCTGTGAACGGCAAGCTTACTGCAATGAATAAGCTGCTGATGGAAGAGAATGATCGACTGCAGAAACAGCTCGCTCAGTTGCACTATGAGAACAACTATCTACGACAACAGGGAGGTATAGCTACTACTGATACGAGCTGTGATTCAGTTGTTACAAGTGGCCTTCAACACGTTAATGCCACCCCTCAACATCCTGCAATCGATGTAACACATTCTGGTATCTTATCTCTCGCGGAAGAGGCTTTGACAGAGTTCTTAGCCAAAGCTACAGGAACTGCCGTGGAATGGGTGCAGATGCCTGGAATGAAGCCTGGTCCAGATTCCGCTGGAATGGTGAATATTGCCCATGGGTGCAATGCTGGGGTAGCAGCACGAGCAGGCGGGATGGTGGCTCTGGAGCCCACAAGGGTGGCTGGCATTTTGAAAGATCGGGCGTCGTGGTGCCGAGACTGTCGTCGATCGAGCACATTGTGGGCCGCGCCTGCAGGCAATGGGGGCACCCTGGAGATATTATACATGCAGATGTACGCACCCACAACAATGGCGCCTGCACGCGACTTCTGTACGCTCCGCTACAGCTCATCGTTAGAGGACGGCGGCTATGTGATATGCGAGCGCTCCTTACTGGGCGGCCTGCACCCGGGCGTGCCCACAGCCCCCCCAATGCCTTCCTTCGCAAGAGCCGAGATGCGCTCCAGTGGCTGCCTCATCCGACCCTGCGGCGCCGGCGGCTCCGTCGTCATTATCGTCGATCATTTTGATCTGGAGCCATGGACTGTGCCCGAGATTGTCCGTCCCCTTTACGAATCCTCAGCTGTTTTGGCGGAGAAAATGTCTCTAGCGGCTTTCCGCCACTTGTCTTGTATTGCACAAGAGTCCTCCATCGATTTGCCCGGGGGAGGGGTCGTGAACCAGCAGCCAGCTGCTGTGAGATCGCTCAGCCACAGGCTGGCAAGGGGTTTTAATGATGCGATTAACACGTTCGTAGACGAAGGATGGACACCGGTTAACAATGCAGATCATGATCATGGGCTGGATGACGTGCAAGTGCTGCTGAAACCTAGCTCAGGCGTGGTGTGCGCAAAAGCGTCTATGCCTCTGCAGGCTGTTTCGCCATCTCTGCTAGTGCGCTTCCTACGCGAGCATCGCTCTGAGTGGGCCGACCACAAGCCTGACCCTAATTCCACTTACTCCTCCGCTAACTTGGCTGCCTCCATGCGCCCCTTCACTCAATACACCGGATCAGCTGACTTCTCCTGCACCTCGCAGCTCCTCTACCCTCAAGTCCACAGCGTGAATCAAGATGAACTTCTGGAACTAATAAAATTGGAGGACTGCAATGACGTGGGACGAGAAGATTCTCTTCTATCTAGGGAGACGCTCTTACTGCAGCATTGTAGCGGATTGGATGAGAAGGCTGCTGGGCCTGCATGTGCCCAGATGGTATTCGGGCCGATTGACACAAACAGTGTGTCAGATGAGGTCCCTCTCTTACCATCAGGGTTTCGGGTTGTCCGATTAGACAGGGGTGGCAATGAAGGAGAAGCTCGTACGAGAACTCTAGACCTAGCGTCGGCTCTGGAGGTTGGGCCAGCCATCAATGGCAGACAGCTGGTTCTCATGGACGGAAGCTGTGGCAATGGATCAGCAGCTGGGGCAGCTCAGTATCAGCATCATCAGTACAGTGCCAATGCAGCTGCCAGATCGTTGCTCACATTAGTATTCCAGTTCCCTGTCGAGAACCGCCTTCAGCAAGATGCCTTGGTACCGCTGGCACGCCAATTTGTTCGCCATGTCATGGCCAACGTCCGCCGCATTGTCACCAACATGGCAGCCCTGCTTCATCATCAGGCTTTGCCTGCCCCTCTTTCCCTGCCACCAGCAGCTGCTGCAAGCCCTGCACCTGCCTCGTCTTCTGAGATAACTGCTCAAGGCCTCACCCTTGGCCACCTCATCTGCAAGAGCTACAAGCGTTTTGTGGGTATGGACCTAGTGCGGGTTAATGCCAAAGATGATGAAGACGATGATGACGGCAAGCAATGCTTCGAGCAACTGTGGCAGTTCTCTGATGCTGTGATGTGTTGTTCTTGCAAGACAATGCCTATGTTTACATTTGCAAATCAAGCTGGCCTGGATATGCTGGAAACCACGCCTGCTGCATTGTACGAGCTTTCATGGGAGGAGACGCTGGATGAGGAAGGCAAAAAGAGCCTTGGCTCTACTCTGGCACAAGTCCTGCAGCAGGGTTATGCATGCCTCCCTGCTGGGGTTCGTCTGTCTAGCACAGGCCGGTTGGCTTCTTATGATCGTGCAATTGCATGGAAGGTTCTTGATGTCAATGACGACACTGTCCATGGTGTTGCATTTGTCTACCTCAATTGGTCTTTCATGTAAAGCATTGCCATCATCATCATCATGTACACTCCATCTCGGAAACAAACATGTAAATTATTCTCCAACCCTCACGGCCATCAGTATCCTGCTTGTACTACTAGTTATTAGCCTTGCTTTAATGATCCTCGTTAAAAAAATATTCAGACCTGTGCTCGAGGTTTCCAAGGTTTTGTACAAGGCTATCATCTAGAAGTAATTTTACATGCCTTGCAAAGAATTACTAGCTCAACAAATCTGGAGCTCCCTTGACGTTTTGTACTAGCCTTATTATCCAACAAAAAAATTCACCAGTTGCGCACCAACCGATTATTCTTCTTTGCATGCCCTTGTTATACATGGTCAAGTAATTCATGGCACGTTTTAAGGTAGATTCTGAGGGCCAATTGAGAAATGTGTGCCCATAGCCGCCACTACTTCAATGCCCAAAACTATATTTCTTGAAATATAAATCCTAATGATATGCTTTGGTGTGTTAAAAAAGCTATGAGCCCTTTGGAAACTTCTAGAGTTCTTTCATTGATGGTAGGTAATGAAACTAAGAGATCTCACGGTGGACAATTGTACTTTAACTCGACACAATATAATATTAAGTTCAGAACCGTTTCTAGTTTAGAAC

**(B)**

**(E)**

**pmi-miR166v1** 20 CCCUUACUUCGGACCAGGCU 1

::::::::::::::::: 3.5

**Lja-Locus_1230** 1390 UGGAAUGAAGCCUGGUCCAG 1409

**(C)**


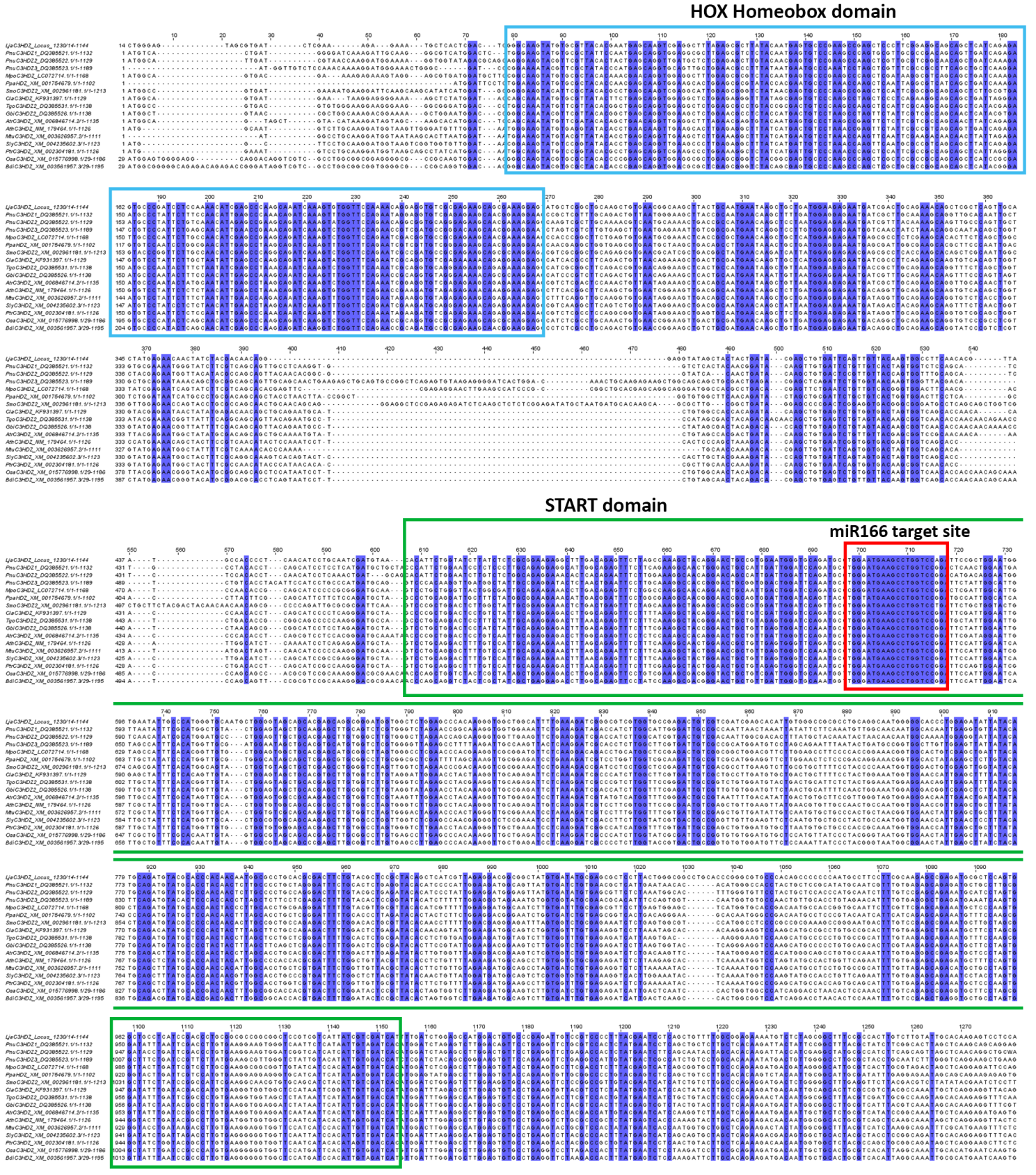

Supplement: S5 Fig — (A) Sequence of a transcript (Locus_1230) encoding an C3HD-ZIP transcription factor protein from the fern L. japonicum. The region predicted to be targeted by pmi-miR166 is indicated in yellow. The starting ATG and stop codon are highlighted in blue. (B) Predicted pairing between pmi-miR166 and L. japonicum Locus_1230. The E-complementarity score between miRNA and target RNA as estimated by the psRNATarget program is shown. (C) Alignment of part of the C3HD-ZIP transcripts from ferns L. japonicum (Lja) and Psilotum nudum (Pnu), the liverwort M. polymorpha (Mpo), the lycopod S. moellendorffii (Smo), the moss P. patens (Ppa), the gymnosperms Cunninghamia lanceolata (Cla), Taxus globosa (Tgl) and Gingko biloba (Gbi); the basal angiosperm Amborella trichopoda (Atr), the dicots A. thaliana (Ath), Medicago truncatula (Mtu), Solanum lycopersicum (Sly) and Populus trichocarpa (Ptr), and the monocots O. sativa (Osa) and Brachypodium distachyon (Bdi). Residues displaying 100% identity are highlighted in blue. The region targeted by miR166 is indicated in red, the START domain in green, and HOX Homeodomain in light blue. Note that the miRNA-targeted region is conserved in all mRNAs and species. (DOCX) [file pone.0177573.s005.docx]
