# Supplementary material for: Sequencing of small RNAs of the fern Pleopeltis minima (Polypodiaceae) offers insight into the evolution of the microrna repertoire in land plants
Source: PLoS One. 2017 May 11;12(5):e0177573. doi: 10.1371/journal.pone.0177573 (PMC5426797; doi:10.1371/journal.pone.0177573)
Supplement: S6 Fig — (A) Sequence of a transcript (Isotig_21625) encoding AGO1 protein from the fern L. japonicum. The region predicted to be targeted by pmi-miR168 is indicated in yellow. The starting ATG and stop codon are highlighted in blue. (B) Alignment of part of the AGO1 transcripts from fern L. japonicum (Lja), the gymnosperm Cunninghamia lanceolata (Cla), the dicot A. thaliana, and the monocots O. sativa (Osa) and Brachypodium distachyon (Bdi). Residues displaying 100% identity are highlighted in blue. The region targeted by miR168 is indicated in red, and the Argonaute N-terminal domain domain (ArgoN) in green. Note that the miRNA-targeted region is conserved in all mRNAs and species. (C) Predicted pairing between pmi-miR168 and L. japonicum Isotig21625. The E-complementarity score between miRNA and target RNA as estimated by the psRNATarget program is shown. (DOCX) [file pone.0177573.s006.docx]

**Fig S6. Predicted targeting of a fern AGO1 mRNA by miR168.**

**(A)** Sequence of a transcript (Isotig_21625) encoding AGO1 protein from the fern *L. japonicum*. The region predicted to be targeted by pmi-miR168 is indicated in yellow. The starting ATG and stop codon are highlighted in blue. **(B)** Alignment of part of the AGO1 transcripts from fern *L. japonicum* (Lja), the gymnosperm *Cunninghamia lanceolata* (Cla), the dicot *A. thaliana*, and the monocots *O. sativa* (Osa) and *Brachypodium distachyon* (Bdi). Residues displaying 100% identity are highlighted in blue. The region targeted by miR168 is indicated in red, and the Argonaute N-terminal domain domain (ArgoN) in green. Note that the miRNA-targeted region is conserved in all mRNAs and species. **(C)** Predicted pairing between pmi-miR168 and *L. japonicum* Isotig21625. The E-complementarity score between miRNA and target RNA as estimated by the psRNATarget program is shown.

**(A)**

>Isotig21625 (AGO1)

CGTGGTTAGCAGCAGCACGTCCTCTCGGCGCACAGCGTCCTTGTTCCGCTTCCTCAGGTGTTCCTTCAACTAAGAGAGCCTTTTCAGTATGCCGAGAAGACGCAAGACCCCAAAGGAAATCGAGGAGAGTTCTCAAGAAGCAACAGCTCAGTCTTCTCAAGGTGGAGCATCTTCTTCAAGTACTCCAGCTCAGGGCCGTgGGCAGCAGCCACCTGCACGCAGTCAGCAGGTTTCACAGCCCTCCGCAGGTAGAGGTCAGCAACCATCTGTACCAACTCCTGCAGGTAGAGGTCAGCTACCACCTGTACAAACTCCTGCAGGTAGAGGTCAGCAGGTGATACAACCTACTCCTGGTAGAGGTCAACAGGTTTCTCATGGTGCAAGTGGAACACAAAGTGTGCCCCCTGGTCAGGTTCCTGTGCAGGTGTTGCAACCTCCTTCAGGTAGAGGTCAGCAGGTCTCTCAGGTCACGGGACCACATAGTTTGCCTGCTGTCCAAGGTCCTCCGCAAGGAAGAGATGGACGAGGTAGAAGTCAAGGACGAGGAAGAGGTGGAGGCCCACCACATCCGAcCCCTCaAATGACTGTTGGTCAGCCTTACGTGCCGACCAACATAATACCTATTCCCGATCTGCCCCAAGCTCACACTACTCCAACTCCATACGGTCTGGAGCAGGCTGCCCACGCTCAATACATGCAGGCTGGCTATGGAGGGATGGCTCCTATTCCAACTATGCAACCACACCTTGTTGCTGTCCCTCAGCCAGCACCTTACCCTGGTATTCCACGTATGGGACAACCATCTCAGCCTCAAGCCAGTTACATGGGCACTTCACCAATCGGTCAGCCAGGCTTTGTTCAGGTTGGTATGTCAGCaTcAAGTTCAACTGTGGACTTGTCACAGCAAATGCAAAGAGTGgGAATACAAGGTCCTCCTCCAGCTATTCAGGAGCCTGGGCCATCAACTGCTCTAGCAGTTGCTCCGCCTGCATCCAGCAAGAAGTTGCGGTTCCCtGCaCGTCCTGGGATGGGAAGAGTAGGGATGCGCTGcACtGTGAAAGCTAACCACTTCTTTGCAGCAcTACCGGACAAAGATTTGCACCAATATGATGTTTCAATaACTCCCGAGGTAACCTCTCGGGGTGTCAACAGGGCTGTAATGCAGCAGCTTGTGCAGCACTATAGAATGAGTGCTCTGGGcTGCAGGCTACCAGCTTATGATGGACGCAAGAGCCTCTACACGGCaggTCCTCTTCCCTTTCAAAACAGGGAGTTTTCAATCACACTaGTGGATGATGATGATGGAAGTGGAGGGCCAAGACGAGAAAGACAGTTTAAAGTGGTAATCAAGTTTGCtGCTCGAGCTGATTTGCATCACCTGGGAGAGTTtCTTCAAGGCAGACAACTAGATGCACCTCAAGAGGCATTGCAGGTCTTAGATATTGTCTTGCGGGAGCTGCCAACACACAGGTTCTGTCCTGTTGGTAGATCATTCTACTCtCCTAATCTTGGTAGACGGCAACAACTTGGGGAAGGATTAGAGAGCTGGCGTGGATTTTATCAGAGCATTAGACCCACTCAGATGGGGCTATCTCTCAACATTGACATGTCTTCAACAGCGTTTATTGAGCCATTGCCGGTCATCGAGTTTGTTGCACAGTTGTTGGGAAAAGATGTTTCTAGGCATTTgTCGGATGCTGATAGAATAAAGATAAAAAAAGCCCTGCGAGGAGTCAAGGTAGAGGTCACTCATCGAGGGACAATGCGGAGAAAGTATCGGATATCTGGATTGACCTCaCAGCCAACCCAGGAaTTGACCTTTCCGGTcGAtGAACAAGGGACAATGAAGTCTGTGGTAGAGTATTTTCAAGAAACATATGGATACACTATCCGTAACACCACGCTTCCATGTCTTCAAGTGGGAAACCAACAGAGGCCAAATTACCTGCCAATGGAGGTGTGCAAGATTGTTGAAGGGCAGAGATACTCTAAGCGATTGAATGAGAAACAAATCACGGCACTGCTGAAGGTCACTTGcCAGcGACCTGTGGACAGAGAAAGAGACATTTTAGAGACAGTACATCATAACTCTTATGCtGAAGATCCTTATGCGAATGAGTTTGGTATACGTATCAGTGAGCAACTTGCTTCAGTTGAAGCCCGTGTATTGCCGGCCCCTAAGCTCAAGTACCATGATACggggaaaTTAAAGTATTGCCAGCCAGAGATTGGACAGTGGAACATGAGAGATAAGAAAATGGTGAATGGTGGAGTAGTCACaTATTGGGCATGTATCAATTTCTCCCGAAGtGTGCAaGATACTGCAGCTCATAGATTCTGTCACGAGCTGGCTtCAATGTGCCAAGTTTCtGGgATGTCTTTCAATGAACGCCCTGCAATTCCAATACACTCAGCACGACCTGATCAAGTTGAGCGAGCATTGAAAAATGTGTTTCGAGAATTTCAACAGAAAGAgAAAGGGCATGATCTTGAGCTTCTcGTTGCTATTTTACCTGATAATAATGGAACCCTCTACGGGGATCTCAAGAGAATATGTGAGACAGATCTtGGCCTGGTCTCTCAATGTTGTTTAACTAAGCACGTGTATAAGATGAGTAAGCAaTACCTGGCgAAtGTCGCGCTgAAAATCAATGTCAAGGTgGGTGGAAGGAATACtGTACTGGTGGATGCTCTCTCTCGCCAAATACCACTTGTCAGTGAcATCCCTACTATTATCTTTGGAGCTGATGTTACTCATCCCCATCCAGGAGAAGATTCTAGTCCGTCTATTGCTGCtGTGGTGGCCTCACAAGATTGGCCGGAAGTTACTAAATATGCAGGGCTGGTGTGTGCTCAAGAGCATCGTCAAGAATTGATACAAGATTTGTTCAAGAGTTGGGATGATCCTGCCCGTGGCCCACAAACGGGTGGcATGATAAAGGAGCTTCTgATATCTTTcCGACGAGCaACGGGTCAAAAGCCTCATCGAATTATCTTTTACAGGGATGGTGTTAGTGAGGGTCAGTTTTATCAAGTACTATTGTATGAGCTgGATGCAATTCGCAAGGCATGTGCTTCTCTGGAATCAAACTACCAACCTCCTGTCACATTTGTAGTGGTTCAGAAGCGCCACCATACACGaCTTTTTGCAAGCAATCATAATGATAGGCGAAGCATAGACAGCAGTGGAAACATTCTTCCAGGCACGGTGGTTGATTCAAAGATATGCCATCCTACAGAATTTGACTTCTATCTTTGCAGCCATGCTGGCATCCAGGGAACTAGCCGACCAGCTCATTACCATGTGTTGTGGGATGAAAACAAGTTCACAGCAGATGGGTTGCAGCTACTtACCAACAATCTTTGCTACACATATGCgCGCTGTACACGTTCTGTCTCAATTGTGCCCCCAGCATATTATGCgCACCTGGCAGCTTTTCGTGCACGGTTTTACATGGAACCTGAGTCgGATAGTGGTTCCCTTACTAGTGCAGCCCCACCACCCAGTAGGGGCACAGGTTCTGGTGCTTCGCGAGCTACtCGTGTGCCAGCTGGGGCTTCTGTCCGGCCTTTGCCAGCTGTGAAAGaAAATGTGAAGCGAGTGATGTTTTACTGCTAGGATCTTGGATTTGTAGCAGCTaACCTAAACTTGTACATGTGTAAAATGaGTTGTCTTTGAGTAAGGATGCCAGTAGTTATTGTCAAAGaAAACTAATGTGAACTCaGACTATGTGGTTTTGGCATTTTTTGTTGCCAGCTGTGTTATAGCCAATTTTGGGCTATTTGTATTGTAACATGTATATTCC

**(B)**


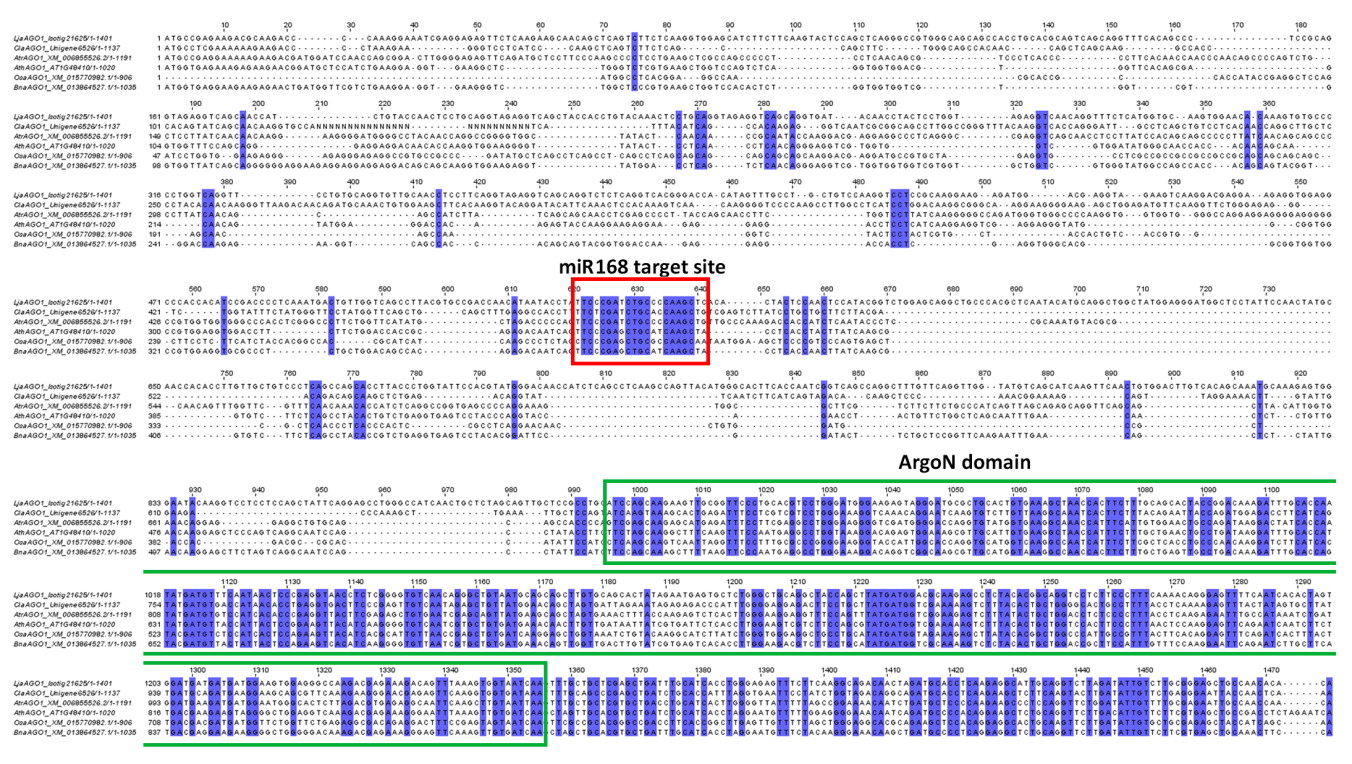


**(C)**

**(E)**

**pmi-miR168v1** 21 AAGGGCUGGACGUGGUUCGCU 1

:::::::.:::: :::::: 4.0

**Lja-Isotig21625** 624 UUCCCGAUCUGCCCCAAGCUC 644
