# Supplementary material for: Sequencing of small RNAs of the fern Pleopeltis minima (Polypodiaceae) offers insight into the evolution of the microrna repertoire in land plants
Source: PLoS One. 2017 May 11;12(5):e0177573. doi: 10.1371/journal.pone.0177573 (PMC5426797; doi:10.1371/journal.pone.0177573)
Supplement: S7 Fig — (A) Sequence of a transcript (Locus_11016) encoding a Scarecrow-like(SCL)/GRAS domain transcription factor protein from the fern L. japonicum. The region predicted to be targeted by pmi-miR168 is indicated in yellow. The transcript is incomplete and lacks the starting ATG, but the stop codon is highlighted in blue. (B) Alignment of part of the SCL transcripts from fern L. japonicum (Lja), the liverwort M. polymorpha (Mpo), the lycopod S. moellendorffii (Smo), the moss P. patens (Ppa), the gymnosperms Pinus tabulliformis (Pta) and Pinus radiata (Pra); the basal angiosperm Amborella trichopoda (Atr), the dicots A. thaliana (Ath), Solanum lycopersicum (Sly) and Populus trichocarpa (Ptr), and the monocots O. sativa (Osa) and Brachypodium distachyon (Bdi). Residues displaying over 75% identity are highlighted. The region targeted by miR171 is indicated in red, and the GRAS domain in green. Note that the miRNA-targeted region is conserved in all mRNAs and species. (C) Predicted pairing between pmi-miR171 and L. japonicum Locus_11016. The E-complementarity score between miRNA and target RNA as estimated by the psRNATarget program is shown. (DOCX) [file pone.0177573.s007.docx]

**Fig S7. Predicted targeting of a fern SCL transcription factor mRNA by miR171.**

**(A)** Sequence of a transcript (Locus_11016) encoding a Scarecrow-like(SCL)/GRAS domain transcription factor protein from the fern *L. japonicum*. The region predicted to be targeted by pmi-miR168 is indicated in yellow. The transcript is incomplete and lacks the starting ATG, but the stop codon is highlighted in blue. **(B)** Alignment of part of the SCL transcripts from fern *L. japonicum* (Lja), the liverwort *M. polymorpha* (Mpo), the lycopod *S. moellendorffii* (Smo), the moss *P. patens* (Ppa), the gymnosperms *Pinus tabulliformis* (Pta) and *Pinus radiata* (Pra); the basal angiosperm *Amborella trichopoda* (Atr), the dicots *A. thaliana* (Ath), *Solanum lycopersicum* (Sly) and *Populus trichocarpa* (Ptr), and the monocots *O. sativa* (Osa) and *Brachypodium distachyon* (Bdi). Residues displaying over 75% identity are highlighted. The region targeted by miR171 is indicated in red, and the GRAS domain in green. Note that the miRNA-targeted region is conserved in all mRNAs and species. **(C)** Predicted pairing between pmi-miR171 and *L. japonicum* Locus_11016. The E-complementarity score between miRNA and target RNA as estimated by the psRNATarget program is shown.

**(A)**

>Lja_Locus_11016 _Transcript_2/5_Confidence_0.667_Length_1933

GCCGACCGCGTCGCCGTCGACAGACGACGGAACCTCCGACGTCTAGAGGGCAACATAGCCATGGCTCAAGCGATATTGGCGCGGCTCAATCACCTAAGCTCTCCCCAAGGGCATCCCTCGCAGCGTGCGATCTTCTACTTCAGGGAAGCGGTCGCCAAGAGGGTTGCCCCGAGCTTAGGCTCCTCTTCTTCGTCCTCGTCCTCCTCCTCTCCCCCTTCTCTTCTTCAACAGCAACCCTTCGACGTAATTGCCAAGATCGGCGCTTACAAGAGCTTCTGCGAAGCCTCACCGATACCTCAATTTGCGCAATTCACTGCCAATCAGGCTATTCTAGAGGCGATGGAAGGGGAGGATGTGGTGCATATTATCGATTTCGAGTTGGGGTTAGGCGGGCAGTGGGCTTCATTTATGCAGGAATTGTCCCAGCGGAGCAGGGGACCCCCGCAGCTGGTGAAGATAACGACGATAGCGGGGAGTGGGGGTTCGATGGAGATGCAATTAGCGAAGGAGAATCTGGTGCAGTTTGCGAGGGAGCTGGGGGTGAACCTCTTGGTGGAGGCGGTGGTAGCAGTGGGACTGGGGGGGCTGAGGGCGGGGATGGTGAAGCGGGGGGAGGGGGAGGCGGTGGCAGTGAATTTCGGATACGGGCTGGGGAGGGCGCTGTCGGAGTTTGCGGCGTCGGCGGAGGCGCTGGTGGCCTTCCTGCAGCTGGTGAGGGGCCTGGCGCCCAAGGTGGTGACAGTGGTGGACTGCGAGTACGGGCTGGAGGTCGGCGGGGCGGCGCAGCACCTGCTGGAGGCGCTGCGCTTTTACGCCTGCGTGTGCGAGTCGGTGGAGTGCGGGGGCGGCGTCAGGCTGGCCCCAGAGGCCGAGAGGATGGTTCTGGGCCCCAAGATAGAGGCCCTGGTGAGGGCCAAAACTTCATTATCATCATCAAAAGCAGTCCCTATAACTTCATGTTCTCCTTCTACATCTGCTGCTTCTGCATTGGAGTGCCTCCCCCCTTGGCGTCTCCTCCTGCAGAGCACTGGCTTCTCCCCCTCTCCGCTCAGCCACGCCGCCGAGACGCAGGCCGCCTGCCTCCTCAAGCACCCCTCCCACAGCGGCTTCTCCCTCAAAAAACAGCATGGCATCCTCTATCTCCTCTGGCTCAACACCCCTCTCTTAGCTGCCTCCTCATGGGTTTAA

**(B)**


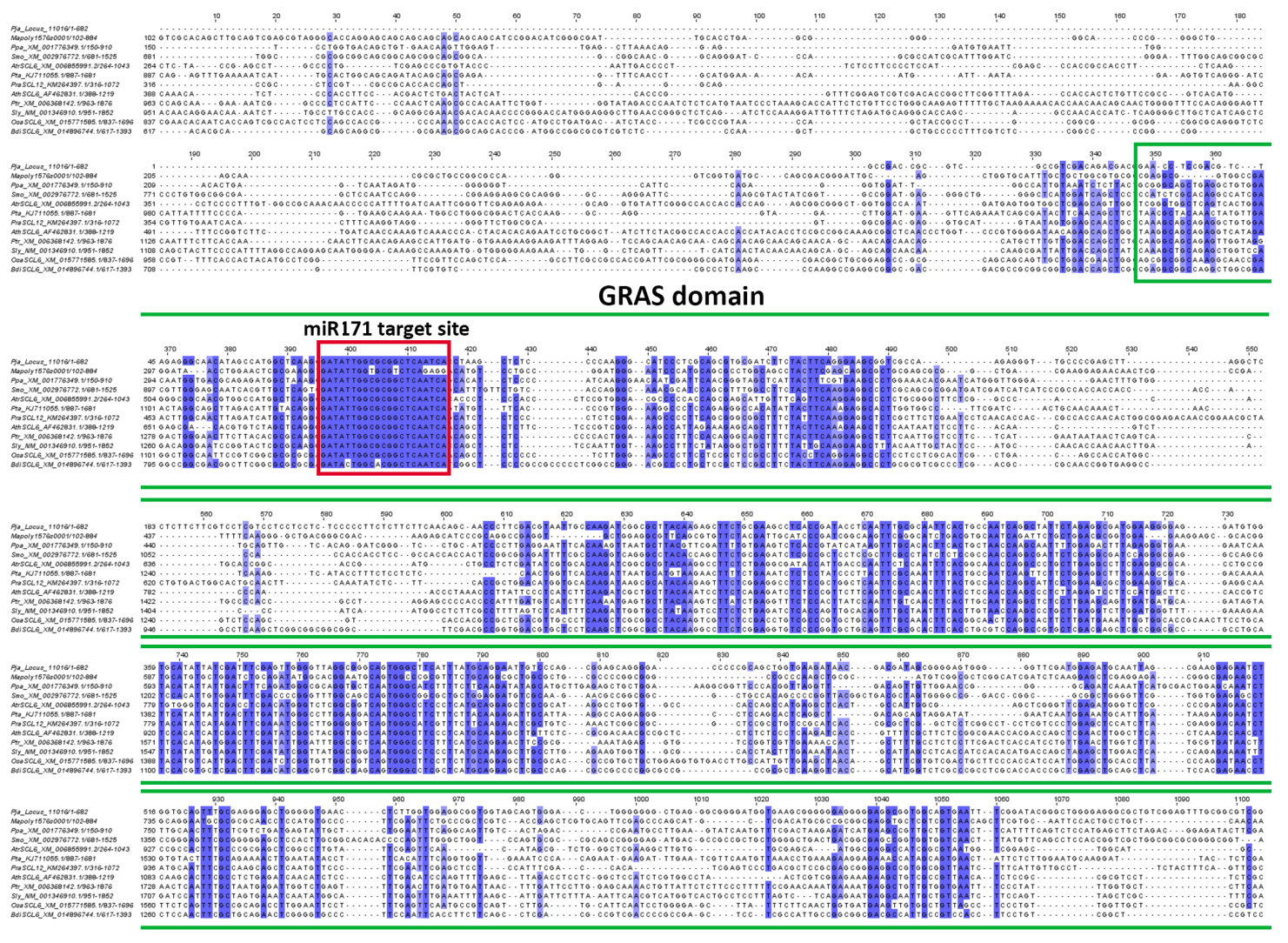


**(C)**

(E)

**pmi-miR171v1** 22 UACACUAUAACCGUGCCGAGUU 1

: : :::::::::.:::::::: 1.5

**Lja-Locus_11016** 68 AAGCGAUAUUGGCGCGGCUCAA 89
