# Supplementary material for: Sequencing of small RNAs of the fern Pleopeltis minima (Polypodiaceae) offers insight into the evolution of the microrna repertoire in land plants
Source: PLoS One. 2017 May 11;12(5):e0177573. doi: 10.1371/journal.pone.0177573 (PMC5426797; doi:10.1371/journal.pone.0177573)
Supplement: S8 Fig — (A) Sequence of a transcript (Locus_9880) encoding a Apetala-2 transcription factor protein from the fern L. japonicum. The region predicted to be targeted by pmi-miR172 is indicated in yellow. The starting ATG and stop codon are highlighted in blue. (B) Alignment of part of the AP2 transcripts from ferns L. japonicum (Lja) and Ceratopteris thalictroides (Cta), the gymnosperms Pinus tabulliformis (Pta) and Ginkgo biloba (Gbi); the basal angiosperm Amborella trichopoda (Atr), the dicot A. thaliana (Ath, and the monocots O. sativa (Osa) and Brachypodium distachyon (Bdi). Residues displaying over 75% identity are highlighted. The region targeted by miR172 is indicated in red, and the AP2 DNA-binding domain in green. Note that the miRNA-targeted region is conserved in all mRNAs and species. (C) Predicted pairing between pmi-miR172 and L. japonicum Locus_9880. The E-complementarity score between miRNA and target RNA as estimated by the psRNATarget program is shown. (DOCX) [file pone.0177573.s008.docx]

**Fig S8. Predicted targeting of a fern AP2 transcription factor mRNA by miR172.**

**(A)** Sequence of a transcript (Locus_9880) encoding a Apetala 2 transcription factor protein from the fern *L. japonicum*. The region predicted to be targeted by pmi-miR172 is indicated in yellow. The starting ATG and stop codon are highlighted in blue. **(B)** Alignment of part of the AP2 transcripts from ferns *L. japonicum* (Lja) and *Ceratopteris thalictroides* (Cta), the gymnosperms *Pinus tabulliformis* (Pta) and *Ginkgo biloba* (Gbi) ; the basal angiosperm *Amborella trichopoda* (Atr), the dicot *A. thaliana* (Ath, and the monocots *O. sativa* (Osa) and *Brachypodium distachyon* (Bdi). Residues displaying over 75% identity are highlighted. The region targeted by miR172 is indicated in red, and the AP2 DNA-binding domain in green. Note that the miRNA-targeted region is conserved in all mRNAs and species. **(C)** Predicted pairing between pmi-miR172 and *L. japonicum* Locus_9880. The E-complementarity score between miRNA and target RNA as estimated by the psRNATarget program is shown.

**(A)**

>Locus_9880_Transcript_6/9_Confidence_0.636_Length_2723

CTCTCTCTCTCTCTCTCTCTCTCTCTCTCTCTCTCTCTCTCTTACGAAGACCTTAACGCCATCCCGGATGTGCCTTCCTCGCCGCAAAGGTCAGATCCTCATTCTCGCGTGCAGGAAGCTGCTGGCCGAGTGCAGAAGGGGCACAAACAAGAACAAGCATTAGATTCGGGGAAACAATCGGCATGCGATGGACGAGGTAGGCGCTGCGGTGATGATGCGCGTGCACCTCATGCTATATCCATGGCGGCCACAAGCTTGTCCTCGTCACGCGAGCCGCCATTGAACCAAGCATCTCTTGGCACTTCTATGTATAATAAGGCCAATCGGCAAAGGAAAATTTCCTCAATCGCTCACGATGATGATCATCGTCATGACAAGGGATCGCCTGCCGGGAGAGAAGACTCGGGGACATCTGTATCCTCCGTGGTAAACGTGGCTTCCACGGATTTTGAAGCATGCAGCCATGGTGGCAGCCGTCCAGGCGTGGCTAGCGATGACGATGATCGCTATCACAGTGTAGCCTGTAGAGCAGCTTATTGTAGGCCGAGCGCAGAACCTCATTTCCTTCTGCCATCAATCCAGGCGGCATCTCTGCCTGCACCGGAGATCACGTCTGCGTCCAACATCAAGAACTTCATGCCCTCTTGCCAGCTCGTGCCATCTCAAGAGTCCGTAATAACAGCCCCTGCGCTCACCCGCAGCTCCACATGCGGTGGTGCGCCGCCTGATTCCGTGAGAGCTCATTATAACTGGGCCGCTGCCGTAAACTTTAACTCCATCTCTTCTCATCAAGGTGATCGAACGTTCCCGGGGCTCGCTTCGATGACTGCAACGGGCGCACCGGCCGAGGCCGCCGACGACGAAAGGGACGAATCTCAGGATCTTCATCTGCCTCATGGACCTTTCAAATTCAATATTGCCAGCCAGCTCTCGCCTATGGAGGAAGCCGCGAATAGCTGTTCTTTGCAAACAAGGCAACTGTTCCCCTTGCTTGATCCTTCTAATTCTCGGCCCAATAATGAGGCGCCACCACCGACACTCCAGGCCTGCTCTAACAATCGTCCCTTTCTTGTGGGTGGCGCATCGTCGACCTCGTCTACAGTTGAGCAACTCATCAGTAGCGCTGGTGGTCCTGGCTCTCTCTTCCATAGCCGGACGCCGCAGTGGCCAAGGCCAAATTTTGGCTGCCTTGAGAACACAAATTTTGCAGCGTCACTGTCGCAGAGCAACCTTGCCTTATTGCAGCAGCCAGAGCAGCTGCAGCTGCAGCCGCAACCAGTCAAGAAGAGTAGGCGCGGTCCTCGATCGAGGAGCTCGCAGTATCGAGGTGTCACCTTCTACCGCCGCACTGGCAGATGGGAATCGCATATTTGGGACTGTGGTAAACAAGTCTATCTTGGTGGCTTCGATACATCTCATGCGGCCGCCAGGGCATATGACAGAGCGGCAATCAAATTCAGAGGTTTGGATGCAGATATCAATTTCAACCTGAGTGACTATGAAGACGAGCTTAAGCAGATGACTGCTCTTACGAAAGAGGAATTTGTTCACATCTTGAGGCGCCAGAGTACAGGCTTTTCACGAGGGAGCTCTCGATATAGGGGTGTAACATTGCATAAATGTGGAAGATGGGAAGCCCGGATGGGCCAATTCCTAGGCAAAAAGTACATATATTTAGGACTGTTTGATACTGAAATTGAAGCTGCAAGGGCATATGACAGAGCAGCAATTCGGTGCAATGGAAGGGAGGCTGTTACGAATTTTGATCCTAGCCTATATGAACATGATTTAGCCATGGAGGCAACCAATGTGGAGGAATGTGAGCAGAGCTTGGAGCTGAGCCTCGGATCAAGGCCATTTAGCACCAGAGAAAGCTCACCAATCGAAACTGCTGCTATTGAGGGATCTCATATGTGGAGTAACTCTCCATTTAAACAGCAGGACTCTCAAAAACCAGCGCCTCTACGGGCTGCAATCAGGCAACTTGAATCCTGGCACTCCAATATGATGCCAGAAACTTTTCTGGCAACCCAAGATGGAGTAAAGGAGCAACGAAAAGGCTCCTATTGTACTGTGGCTCCAACATTGCTAGAACCTCCCAGTCGTTGGACTTGGCAGCAACTCCAGCAACTTGCACCACTGAATGCATCAACCTTGTATGCAACTGCAGCATCATCAGGATTCTCACCCCAACCACCCAAAGGTGGCTTTGGATGCCTGGATTGGCTTCATAAAACAGGACTCAGCTACCTCAATTGTCATCCCATCAGTAGTACGGATCAGTCTTCACACAGTTCAAATTTGCTGCCTGACCATTCTAACGCCAGTGATCAGTCCCTTGTTTTCCAAATGAGCTCCAATAAGAAGCTTAAGGGCCTTGGGTGCTAGTAAAACCCAAGGAAACTTACGTTAAAAATTACTCTCCCAATTATAATTCTACAGGAGAGTGACTTTGTAGTTTGGTTGCTGAGAATTTTGACTTTGTGATTCTCAACACGAGTGTACGGCTAATTATTCCTTGGTTGAGCTAGAGTTGTGATCTTCATCAGAAAACGAGATCACAACTTTTTCATTGTATTTTTTGATGGAGAATAATGGGATGTGCTCTGATTTTGATCGAATGAGAGATGTAGTCCCATTTCTTCTCAAGAGTTTGCCATGTAGAACTAACTTGTCTTACCATCTTAAACATTTCAATTGAAGCGGCGTTGCATGCATGCTTT


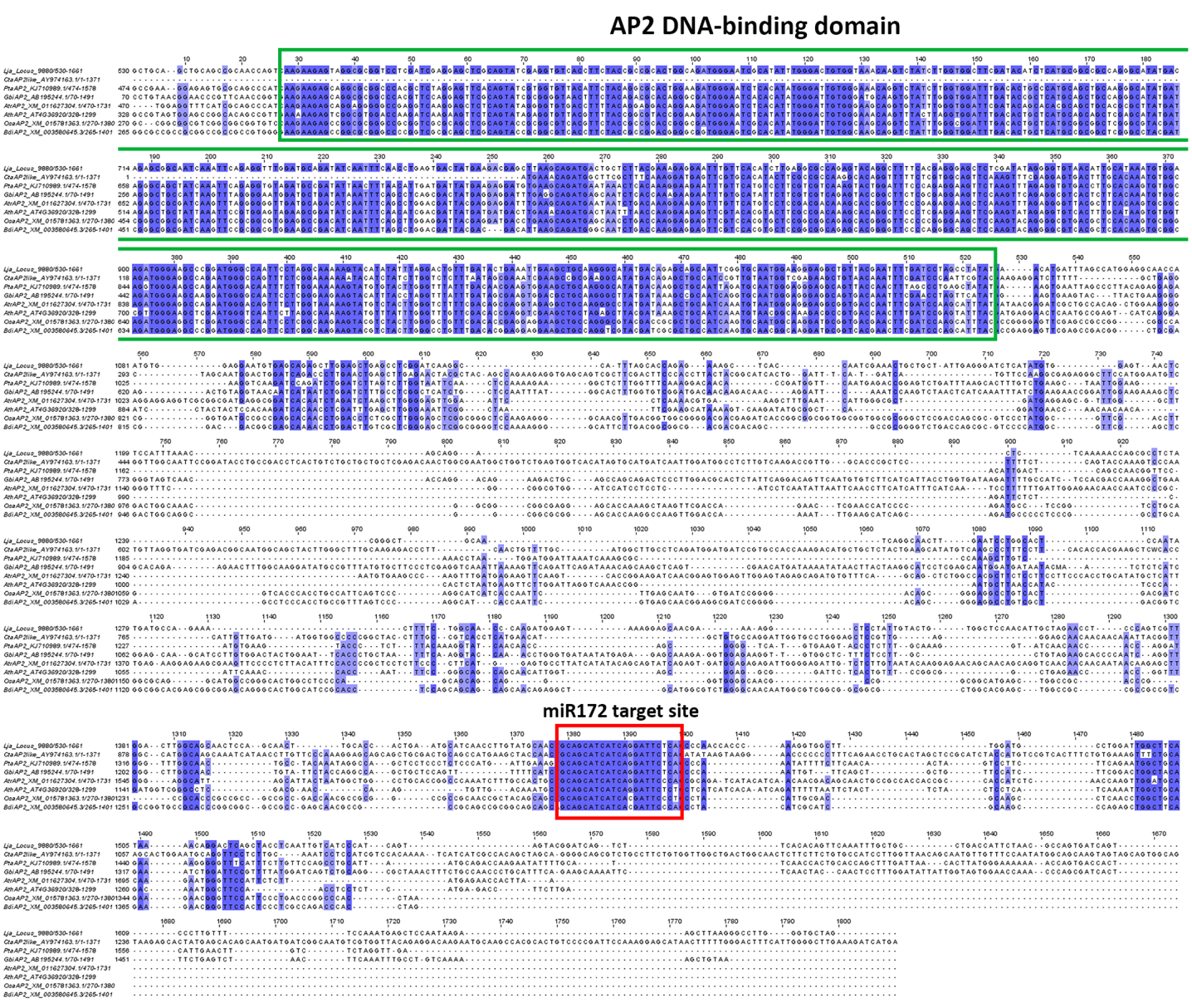
**(B)**

**(C)**

(E)

**pmi-miR172v3** 21 CGUCGUAGUAGUUCUAAGAGU 1

::::::::::::.:::::::: 0.5

**Lja-Locus_9880** 2167 GCAGCAUCAUCAGGAUUCUCA 2187
