# Supplementary material for: Sequencing of small RNAs of the fern Pleopeltis minima (Polypodiaceae) offers insight into the evolution of the microrna repertoire in land plants
Source: PLoS One. 2017 May 11;12(5):e0177573. doi: 10.1371/journal.pone.0177573 (PMC5426797; doi:10.1371/journal.pone.0177573)

**Fig S9. Predicted targeting of fern *TAS3* RNAs by miR390.**

**(A)** *TAS3* RNAs (Locus 20755 and Locus 39179) from *L. japonicum* predicted to be targeted by pmi-miR390. The regions predicted to pair with miR390 are indicated in blue, and the trans-acting small-interfering RNAs (tasi-RNAs) derived from *TAS3* processing are shown in yellow. The tasi-RNAs are predicted to target mRNAs encoding ARF3/4 transcription factors. **(B)** *TAS3* RNAs were aligned with the CLUSTAL Omega program. Regions predicted to be targeted by pmi-miR390 are indicated in blue, with the base pairing with miR390 indicated. Note that the pairing between pmi-miR390 is more extensive at the 5' site than at the 3' site, indicating that RISC-mediated cleavage of the RNAs probably occurs only at the 5' site. The tasiRNAs derived from *TAS3* processing are shown in yellow. **(C)** Alignment of part of the *TAS3* transcripts from *L. japonicum* (Lja) and a *TAS3* from the dicot *A. thaliana* (Ath). Residues displaying 100% identity are highlighted. **(D)** Sequence of a transcript (Isotig23398) encoding a ARF transcription factor protein from the fern *L. japonicum*. The region predicted to be targeted by pmi-tasiR-ARF is indicated in yellow. The starting ATG and stop codon are highlighted in blue. **(E)** Alignment of part of ARF transcripts from fern *L. japonicum* (Lja), the gymnosperm *Pinus pinaster* (Ppi); the basal angiosperm *Amborella trichopoda* (Atr), the dicot *A. thaliana* (Ath) and the monocot *Brachypodium distachyon* (Bdi). Residues displaying 100% identity are highlighted. The region targeted by pmi-tasiR-ARF is indicated in red, and the B3 DNA binding and the Auxin responsive factor domains are also indicated. Note that the tasiR-ARF-targeted region is conserved in all mRNAs and species.

**(A)**

>Locus_20755_Transcript_3/3_Confidence_0.667_Length_762 (ARF4)

AGAGAGAGAGAGGGAGAGAGAGAGAGAGAGAGAGAGAGAGAGTGTCGTGGCGGCTTCGGCGTTCATCTCGACCTTGCTTTCTGGTCTTTCTTCGCCCACATACATATGATGATCTTGCTCGCTGATCATATGGCGGCGTTTCTTTTGCAAGTCGGCAGCTGCAGTACCACAACTGAGATGGAGTCTAGTCAAATCACCAACCGAATCAAGAAGAAGATGCAAAGGAGAACTATAAGCTCTCGTCTCCGTGCCGGACGAGAGCCGTCAATACGTAGCCCATCCTCTCACCACGAGCACCAGCTATCGACATGCAGTATCCCTTCTCTCTCTGGCAAGTTCCCATTGGATCAACAGCTCAACACATTGACCCATATCCTTTACATCATGCAGATCCTAGTTACAACACTACTAATAATCCTGTATCTCCTCATGTTAACCTGCCCTCGGATACTCCTTGGTGCTATCCCTCCTGAGCTTACTCCACAACCCTTCCACATTTGCCAACCCTTAGCCTACCCTTTGCTTGCCTTCTCTTGACCTTGCAAGACCTTAGACAATGCCACTCCGGAACTCTTACTTTTACGTGTTTCCGTCAATCTTAGTACTTCCCAGGAGTGGCATCTCTAATTCAACACCTCACCTATGTATTCTCTATCCCTCTTGAGCTTTTTCGATAAGCCTCAAATATGGCTGAAAACATGCATTCCATATTGATTTGTATGTAAGTTCTTGTAAGGTGGAAGACTACCATGTCAGTCTTCA

>Locus_39179_Transcript_1/1_Confidence_1.000_Length_745

GAGAGATAACGTATTAGTGTGAGAATGTGCATTGTGCTGGTAGCTAAATATATATACATTGGTGCACGCGCACATTGCAGTCCTGGATCCACTACAAAATCAAGAAGAAGCAGATCAGGTCAGCACACAGACACAGAGAGAGAGACAGAGATCAGAGAGAGAGAGAGATGATAGATCTGATCTGAGAGAGACTCTATTCCCAAGGTTGCCTTAACGTGCTTAATTAATTATTTAGTATGTCATGTATCCCTGAGAGTGGAGATGCACCTCCTATTCTGTACTTATATGAGAGATCAACCCATCAATGGCTGCATGACAGATTCGGATACAATCATGCAAAGTACAATGGGAATATATGCCCACAACATGACAGTATGCGTGGTGATGATTACTATACACAAGCAGTTATACTACCACCATGAATATAATTATTGATGAAGCATGGGCGCTATCCTTCCTGAGCTTGAGCCTCGCCATCTCCGAGTCCCATGGCAAGCCCTTCTGCTTCCCTTTTGGTCCTTCTTGACCTTGCAAGACCTTCTCTTGGCCTTGCCAGGCTTCCTAATCAGAGAGCAGCTCAATCCATCTTCTTACCCCTACTCACCATCATATCCTCTTGTCTATCCCTCCTGAGCTTCCCATGTTCCTATCATCGAGTTCTTTAATCCCATCAAAGCAAATAGCTTATGTACATTTAGTTTCCTCTTACAGACAAATTACTTGATAGATGATCGCATGTAACAGC

blue: regions targeted by *P. minima* miR390

yellow: region that targets ARF3/4 mRNAs

**(B)**

Locus_20755 AGAGAGAGAGAGGGAGAGAGAGAGAGAGAGAGAGAGAGAGAGTGTCGTGGCGGCTTCGGC

Locus_39179 -GA------------GAGATAACGTATTAGTGTGAGAATGTGCATTGTGCTGGTAGCTAA

** **** *..*:.: **:*:****.:*:* .* *** ** : * ..

Locus_20755 GTT------C-------ATCTCGA-CCTTGCTTTC-TGGTCTTTCTTCGCCCACATACAT

Locus_39179 ATATATATACATTGGTGCACGCGCACATTGCAGTCCTGGATCCACTACAAAATCAAGAAG

.*: * .:* **. *.****: ** ***: :**:*....:**:..*

Locus_20755 ATGATGATCTTG----CTCGCTGATC------ATATGGCGGCGTTTCTTTTGCAAGTCGG

Locus_39179 AAGCAGATCAGGTCAGCACACAGACACAGAGAGAGAGACAGAGATCAGAGAGAGAG---A

*:*.:****: * *:*.*:** . .:.:*.*.*.*:* . : :*..** .

Locus_20755 CAGCTGCAGTACCACAACTGAGATGGAGTCTAGTCAAATCACCAACCGAATCAAGAAGAA

Locus_39179 GAGATGATAGATCTGATCTGAGAGAGACTCTATTCCCAAGG-TTGCCTTAACGTGCTTAA

**.**.:. * *: *:****** .** **** **..*: . :.** :*:*.:*.: **

Locus_20755 GATGCAAAGGAGAACTATAAGCTCTCGTCTCCGTGCCGGACGAGAGCCGTCAATACGTAG

Locus_39179 TTA------ATTATTTAGTATGTCATGTATCCCTGAGAGTGGAG-------------ATG

:: .: *: ** :* **: **.*** **. .*: *** ::*

Locus_20755 CCCATCCTCT-CACCA--------CGAGCAC----CAGCTA--TCGACATGCAGTATCCC

Locus_39179 CACCTCCTATTCTGTACTTATATGAGAGATCAACCCATCAATGGCTGCATGACAGATTCG

*.*.****.* *: * .***.:* ** *:* * .****... ** *

Locus_20755 TTCTCTCTCTGGCAAGTTCCCATTGGATCAACAGCTCAACACATTGACCCATATCCTTTA

Locus_39179 GATACAATCATGCAAAGTACAATGGGAATATATGCCCACAAC--------------ATGA

: :*:.**: ****. *.*.** ***: *:.:** **..** :* *

Locus_20755 CATCATGCAGATC-CTAGTTACAA--CACTACTAATAATCCTGTATCTCCTCATGTTAAC

Locus_39179 CAGTATGCGTGGTGATGATTACTATACACAAGCAGTTATACT--ACCACC--ATGAATA-

** ****. . .*..****:* ***:* *.*:**.** * *:** ***:::*

**pmi-miR390_v1** 3' CCGCGATAGGGAGGACTCGAA 5'

||.|||||||.||||||||||

Locus_20755 CTGCCCTCGGATACTCCTTGGTGCTATCCCTCCTGAGCTTACTCCACAACCCTTCCA---

Locus_39179 TAATTATTGATGAAGCATGGGCGCTATCCTTCCTGAGCTTGAGCCTCGCCATCTCCGAGT

:. .* *.: *. *.* ** ******* **********.. **:*..*. ***.

**ARF3/4 tasi-RNA**

Locus_20755 -CATTTGCCAACCCTTAGCCTACCCTTTGCTTGCCTTCTCTTGACCTTGCAAGACCTTAG

Locus_39179 CCCATGGCAAGCCCTTCTGCTTCCCTTTT---GGTCCTTCTTGACCTTGCAAGACCTTCT

*.:* **.*.*****. **:****** * ********************.

Locus_20755 ACAATGCCACTCCGGAACTCTTACTTTTACGTGT----TTCCGTCAATCTTAGTACTTCC

Locus_39179 -CTTGGCCTTGCCAGGCTTCC---TAATCAGAGAGCAGCTCAATCCATCTTCTT------

*:: ***: **.*.. ** *::*..*:*: **..**.*****. *

**pmi-miR390_v1** 3' CCGCGATAGGGAGGACT

. |||||||||.|||

Locus_20755 CAGGAGTGGCATCTCTAATTCAACACCTCACCTATGTA-----TTCTCTATCCCTCTTGA

Locus_39179 -------------------ACCCCTACTCACCATCATATCCTCTTGTCTATCCCTCCTGA

:*..*:.******:: .** ** ********** ***

CGAA 5'

||||

Locus_20755 GCTTTTTC-------------------GATAAGCCTCAAATATGGCTGAAAACATGCATT

Locus_39179 GCTTCCCATGTTCCTATCATCGAGTTCTTTAATCCC-A-TCAAAGCAAATAGCTTATGTA

**** . :*** ** * : *:.**:.*:*.*:*. .*:

Locus_20755 CCATATTGATTTGTA------TGTAAGTTCTTGTAAGGTGGAAGACTACCATGTCAGTCT

Locus_39179 -CATTTAGTTTCCTCTTACAGACAAATTACTTGATAGATGATCGCATG---TAACAGC--

***:*:*:** *. : :** *:****::**.**.:.*..*. *.:***

Locus_20755 TCA

Locus_39179 ---


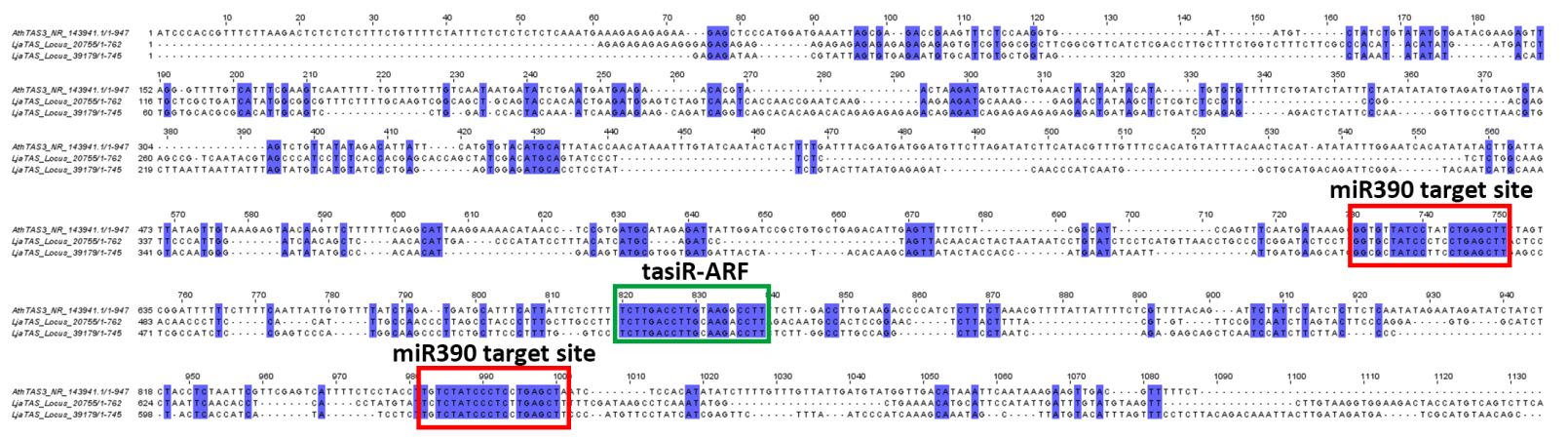
**(C)**

**(D)**

> Isotig23398 (ARF3/4)

AGCAGAGTTCATTATACCTTTGGAACGATTCAAAAAGGCTGTAAGCCAGAATCTTACTGTTGGAGTGAgaTTCAAGATGAAGTTTGAGACCGAAGACTCATCCGAAAGAAGGtatAATGGTACTATCACTGGAGTTGAcgACGTGGATCCAATCAGATGGTCTAACTCGAAGTGGAGGtGTTTGAAGGTagGTTGGGATGAATCTACtATCTATGAAAGGCAAGATAGGGTGTCTCCATGGGAGAtcGAGCTgTGTGTGCCTGCAAcTTCCCCCGCTCCATCCAACAACACAACAtCACTCAGGTATAAaCGTCCTCGTCCTGCTGGTGTgGCTaTcggggCTCCGGCTACACCTGAACGTTCGCCAGCAATGAACCATGGGAGATCAGGCCAGGCAGCCCTAGAATCCTACTTGGCAGCTGCTAGTGCTTGTTTATCGACGGTCTTGCAAGGTCAAGAACCTCGGGCCTCATGTGTGCGCGAGGACaAAGAAAGTGGcCCTAGTTCACATTACCATCATCAAAAtCAGCACCTGCAGCATCACCAtCTTCTACAGCAACAGCATCAcCAACGCCATGTCCAGCAGCAGCAGCAgCAaCAACCACCACCACCACCAcAAGAACAACAACAGaCaGGATGGAGtCCAAGTGCAGGtCCAGAGATGTATTGGCAAATGAAAAATGGGCAACGAAGCCGGGGcGATGGAAGCAGTACAGGAGCCGGTGGAGGAGGAAATaACCCCTTTTCTACTTTCGTTTGCCCTCAGCAGTTCGTGTCTTCCGAGGCCAGTGTCAAAATGGGCGGCCGGCCGCTGCACTGTCCCTCTaCCTCCTTGGGCGCGGCCAATTCGCGGCCCAATTTGAACAGCCAAAACTGGCAGGCCGTAAACATGCACGACACACCAAGTTGGCTCATGAACCCCCTAATGCCCCCTCCACATTCTGCCAAGGCCTCGGGCCTCGCACCCATgaTGGGTGCTCTCGAGAAACCTAACCCAAATACCTCGACTCCTCACGTGGTTGTGAAATGTCAACAGCAGCCATCTCAcAATAATGGGCCCTTTGCTTGTGCTAATCAGAGCTGGGCATTAAAGCAGAATGAcAAGGAATcATGcGAGAcCGCtCCCCCTCCTcCGAcGCCCaCacCCCACTCAGAGAACAGCTGCAAGCTCtTTGGCGtTTCtctAACAGACtTGtCAGCCCCCAGGGTTAGAACgaAAAtAgCGGtaGAAGAGGAGGGCcAACTTGACCCGTCCTCGGCCaTCAAGCAGCAGGGTGCAGCTGGTGCGGTGaTAGCGCaAGAAGCtGCAGAGCACGAAGGTGAGGAGACATCTTGTCGGAATCTCAACATGGTGCCCGAGAATTCGACTATcAGCgCTGAGCAAGACAAGTCTTCTGCACCTCCAGCAAAAGAAGGGCAACTTAAGAGCCACCAAGTCGCAGTCAGAAGCTGTACAAAGGTGGTGATGGAAGGCAGGATAGTCgGGAGAGGCGTAGACTTGTCTAAGTTCGAGGGATATCAGCAGCTGTTTGAGGAGCTTGAGCGCAtGTTTCACATTGAATTgGGGCTTCGAGGGGCAAACCGAGGTTGGCAGGTTGCATACTATGACAACGAAGGCGATATGTTACTAGTGGGCGACGATCCTTGGCAGGAGTTCTGCGAGATGGTGCGCAAAATTCGGATTCTGAGTCCAGACGAAGTAGAAGGGAAGAAGCTATTGGACAGGAGCCATCATGCATCACCAGCAGCGGCTGATCATGCCTCCATACCATTACAAGACACCAAGCTTTGTATAAAGGAAAACAGTACAAAGAGGTATCAACCGGAGGATCATGAAGATACTCCTGTCTGAGTTCAAGAATTAACCCCAGATTTTAGCCGGCTCTTCCAACAGAGTACTCGACGTTAAATTCTGGGGAAGATAACTTACACACTTAGGTGCCGGGgATGACTAAATCCTACCGAAGAATCATAGATGATAGGCCTTTCATGTAAAAATTAATGTCATCCCAATTTTGAAAACCGTGTACTGTGGAGTTGCGATTGTTGGCC

(E)


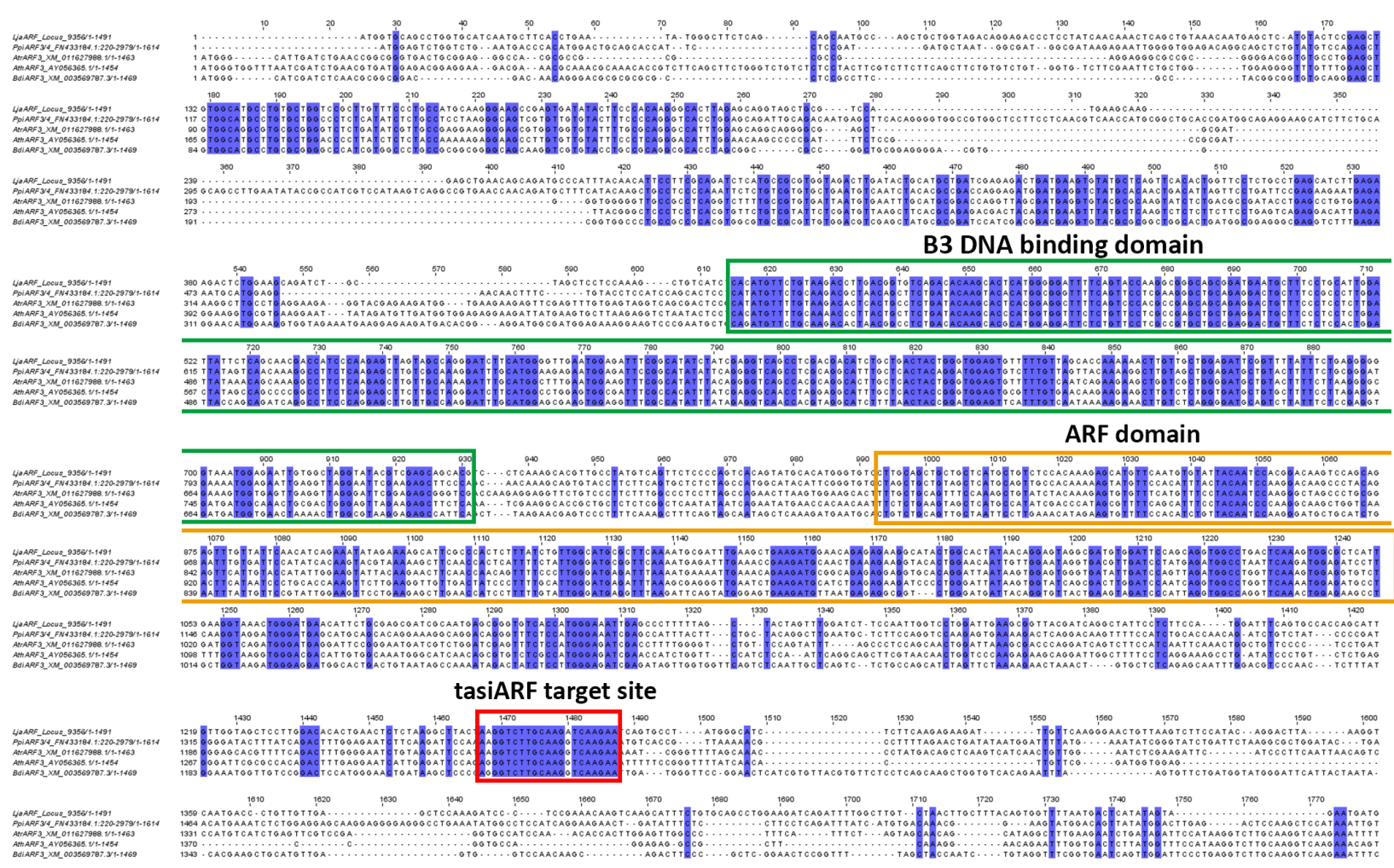

Supplement: S9 Fig — (A) TAS3 RNAs (Locus 20755 and Locus 39179) from L. japonicum predicted to be targeted by pmi-miR390. The regions predicted to pair with miR390 are indicated in blue, and the trans-acting small-interfering RNAs (tasi-RNAs) derived from TAS3 processing are shown in yellow. The tasi-RNAs are predicted to target mRNAs encoding ARF3/4 transcription factors. (B) TAS3 RNAs were aligned with the CLUSTAL Omega program. Regions predicted to be targeted by pmi-miR390 are indicated in blue, with the base pairing with miR390 indicated. Note that the pairing between pmi-miR390 is more extensive at the 5' site than at the 3' site, indicating that RISC-mediated cleavage of the RNAs probably occurs only at the 5' site. The tasiRNAs derived from TAS3 processing are shown in yellow. (C) Alignment of part of the TAS3 transcripts from L. japonicum (Lja) and a TAS3 from the dicot A. thaliana (Ath). Residues displaying 100% identity are highlighted. (D) Sequence of a transcript (Isotig23398) encoding a ARF transcription factor protein from the fern L. japonicum. The region predicted to be targeted by pmi-tasiR-ARF is indicated in yellow. The starting ATG and stop codon are highlighted in blue. (E) Alignment of part of ARF transcripts from fern L. japonicum (Lja), the gymnosperm Pinus pinaster (Ppi); the basal angiosperm Amborella trichopoda (Atr), the dicot A. thaliana (Ath) and the monocot Brachypodium distachyon (Bdi). Residues displaying 100% identity are highlighted. The region targeted by pmi-tasiR-ARF is indicated in red, and the B3 DNA binding and the Auxin responsive factor domains are also indicated. Note that the tasiR-ARF-targeted region is conserved in all mRNAs and species. (DOCX) [file pone.0177573.s009.docx]
