# Supplementary material for: Sequencing of small RNAs of the fern Pleopeltis minima (Polypodiaceae) offers insight into the evolution of the microrna repertoire in land plants
Source: PLoS One. 2017 May 11;12(5):e0177573. doi: 10.1371/journal.pone.0177573 (PMC5426797; doi:10.1371/journal.pone.0177573)
Supplement: S10 Fig — (A) Sequence of a mRNA encoding Polyphenol oxydase (PPO, Locus 5437) from L. japonicum predicted to be targeted by pmi-miR408. The targeted region is located in the 3'-UTR of the mRNA (yellow). The starting ATG and stop codon are highlighted in blue. (B) Predicted pairing between pmi-miR408 and L. japonicum Locus_9880. The E-complementarity score between miRNA and target RNA as estimated by the psRNATarget program is shown. (DOCX) [file pone.0177573.s010.docx]

**Fig S10. Predicted targeting of a fern *PPO* mRNA by miR408.**

**(A)** Sequence of a mRNA encoding Polyphenol oxydase (Locus 5437) from *L. japonicum* predicted to be targeted by pmi-miR408. The targeted region is located in the 3'-UTR of the mRNA (yellow). The starting ATG and stop codon are highlighted in blue. **(B)** Predicted pairing between pmi-miR408 and *L. japonicum* Locus_9880. The E-complementarity score between miRNA and target RNA as estimated by the psRNATarget program is shown.

**(A)**

>Locus_5439_Transcript_1/3_Confidence_0.467_Length_2125

GTTTAACTCTGGTCTCGCGGTTAGTAGGCGGCAGGCCGCTCTCTCGTGTTTGTGGCTAGACATTTGTTGTGTGCACGCTCTTTTTATCGACATCCCTGTTGCAATTTCGTGCGTGTTGTGTAATTTTGTGAGTGTAGGGTAGCCTGCGAGGTAATGAGTTCCCAAAAGCTTTCTGTGCCAGATCTGGGAACAAAAGTTTATAGAAGTGTTAATTTAGTTCCGTTTAGCAAAAATGGATGTCGTCAACACAGTTGTTATTCAGTGGAGGGTGAGAGAGTAGATTCAGCAGCATTCTGCTTAAGCAAACTGCCGTCAATCATACAGTTGGGTTGAGTGAAGCATAAATTGAACTGTCCATTGTCATCTCAAACTAATGGCGATAATGAATGACAGGTTCACAACTCGTGGCTGTTCTTCCCTTGGCACCGGTGGTACCTCTACTTCCACGAGCGCATGCTGGCGAAGCTGCTGGGCGACGACACCTTCGCCTTGCCCTTCTGGAACTGGGATGCGGCGGCGGGGTACACACTGCCGCCCATGTACGCAGACCCGCAAAGCCCCCTGTACAACGCGAACCGCAACCCGGCGCACCGGCCGCCGACGGTGGTGGACCTCAACTACAACTTCACGGACCGCAACCGGACGCCGGCGGCGCAGCTGGCGGCCAACAACAGCATCATGTACCGGCAGCTGGTGAGCAACGCCAAGACGCCGGCGCTCTTCTTCGGGCAGGCGTACCGTCAGGGGGACGAGGCGAGCCCGGGGGGCGGTACGGTAGAGAACGTGCCGCACGGGACGGTGCACTCCTGGACGGGCAACCCGAGCAACCCCAACGACGAGGACATGGGAGCCCTCTACTCAGCCGGGCGCGACCCCATCTTCTTCGGCCACCACAGCAACATCGACCGGCTGTGGGAGGTCTGGAAGGGCCTGGGCGGGCGCCGGACCGATCTGACGGACCCGGACTATCTCAACGCCACCTTCCTCTTCTATGACGAGAACGCGCAGCTGGTCCGAGTCCGGATCGGAGACTCCCTCGACACCCGCAAGCTCGGCTACGTCTATCAGGACGTCCCCAACCCTTGGCTCACTAGACCATCATCATCATCATCGGCTTCTGTTGCAGCAATGTCCAGCTTCCATGGCCCCGCCGGAGGGCCCCCTCCCGCCCTCGCCGGCCACCTCCGCCAGAAGCTCAAAGCGGGCCTCCGTGAGTTCAAAGAACGCGCCTCCAAGACCATCGACAAAATCGAGAGCTCGCTCGTCCGCCGCCCCAAGAAGGTCAAGGCCGATGACTTCGAAGAGGAGATCCTTGTCCTCCAGGGCGTCCAGGTCCCCGTCAATGAGAGGGTCAAGTTCGACGTCTATATCAACCTCCCCGACGCCGACCCCGACGTCGGCTGCGACATCCCTGAGTACGCTGGCTCCTTCTTCAACGTGCCCCACCTGGGCATGATCGACAAGATGGCCATGAAGATGGGTTCGGACGCACCGCGCACCCGGAAGTCCAACTTCAGACTGGGGATCGGTGAAGTCCTCAAAGAGCTCGGCATCGAAGACGACGACAGCTTCAGCATCACCATCGTTCCTCGCTCCAAGACCAGTTTCCCCATCTCCATCGACGGTGTAAAGCTCGAGTACGAGTGATAAGGAAGAAGAATTAAATAAGGATCATGATGCATTGATGATGGTTATTTGGCTAGGGAAGAGACAGTGCCTTCGAATCAGTTGTCTGAACTGCAGTTTACTATAATAAAGAAACCTTTTTAAAGTAAACGTTTCTGCTCGGTTCAAAACTTCAACTTCTAAGTTCTGCTCCTTCGATGTTGGCTTTAGTCAATAGTCTACAGACTACAATTCCTCAAACTTTTGCAATTTTGATGTAGTTGCTAAGTTTCAAAATTTATGCTTAATTGTCAAATTGAGCCCTGTCGCAAAGGCTTCTTATATGCACACACTTATCAAGACAACAAAGTACATTATTATATTGGATACAAAATGATGTGGTTCTATTAGTAATAGTACCAAAATGTCTTAGGATACACTAGTTGGATAAAGGCCAAAAGAAAAGGAGCTTTTCAGGTCAGTTTAGGAGAAAAAGATAGAATGGATTGGATCGAACGTC

**(B)**

(E)

**pmi-miR408v1** 21 UCGGUCCCUUCUCCGUCACGU 1

.::.::::::::: :::::: 2.5

**Lja-Locus_5439** 1698 GGCUAGGGAAGAGACAGUGCC 1718
